# A physicochemical investigation of ionic liquid mixtures†
†Electronic supplementary information (ESI) available. See DOI: 10.1039/c4sc02931c



**DOI:** 10.1039/c4sc02931c

**Published:** 2014-11-05

**Authors:** Matthew T. Clough, Colin R. Crick, John Gräsvik, Patricia A. Hunt, Heiko Niedermeyer, Tom Welton, Oliver P. Whitaker

**Affiliations:** a Department of Chemistry , Imperial College London , London , SW7 2AZ , UK . Email: p.hunt@imperial.ac.uk ; Email: t.welton@imperial.ac.uk; b Tonbridge School , Tonbridge , Kent TN9 1JP , UK

## Abstract

Ionic liquids have earned the reputation of being ‘designer solvents’ due to the wide range of accessible properties and the degree of fine-tuning afforded by varying the constituent ions. Mixtures of ionic liquids offer the opportunity for further fine-tuning of properties. A broad selection of common ionic liquid cations and anions are employed to create a sample of binary and reciprocal binary ionic liquid mixtures, which are analysed and described in this paper. Physical properties such as the conductivity, viscosity, density and phase behaviour (glass transition temperatures) are examined. In addition, thermal stabilities of the mixtures are evaluated. The physical properties examined for these formulations are found to generally adhere remarkably closely to ideal mixing laws, with a few consistent exceptions, allowing for the facile prediction and control of properties of ionic liquid mixtures.

## Abstract 2

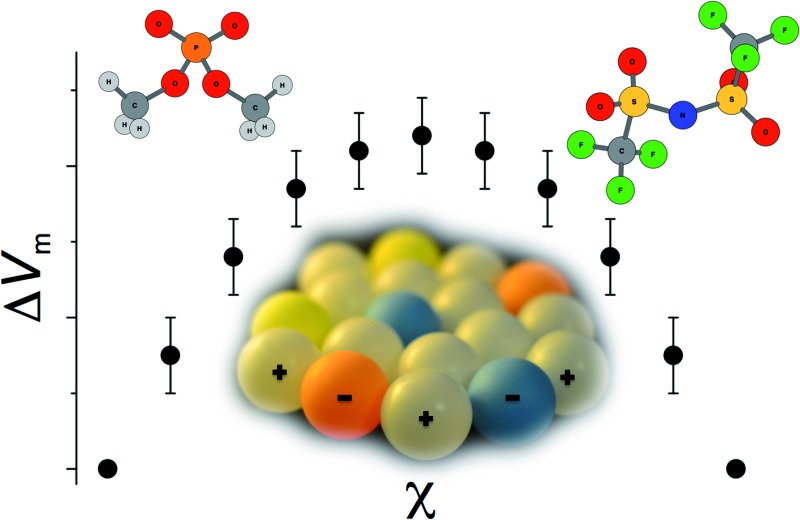
A comprehensive study of ionic liquid mixtures reveals a remarkable adhesion to ideal mixing laws, with some consistent exceptions.

## Introduction

Ionic liquids are, by definition, liquids composed exclusively of ions. Although this definition applies to all molten salts (*e.g.* molten NaCl at >801 °C), the term is commonly used to refer to compounds that are liquid at much lower temperatures, with room temperature ionic liquids (RTILs) being of particular interest.^1^ The broad range of possible cation and anion combinations has led to ionic liquids being labelled ‘designer solvents’,^2^ and RTILs are finding increasing widespread application in synthetic chemistry, in engineering applications,^3,4^ and in dissolution and modification of lignocellulosic biomass.^5^ However, this synthetic flexibility can render it difficult to choose the best ionic liquid for any given application. Further problems arise with the use of such a wide range of compounds outside of academic laboratories, whereby each distinct ionic liquid requires a full analysis under *Registn, Evaluation, Authorisation & restriction of Chemicals*, ‘REACH’ (or similar outside the European Union).^6^ This is a lengthy and costly process.

One viable approach to circumvent these problems is the formulation of ionic liquid mixtures.^7–10^ This offers a number of advantages: (i) the component ionic liquids have well-characterised properties; (ii) syntheses of the simple ionic liquids are well known, and the prepan of mixtures is trivial; (iii) only the pure components of mixtures currently require registn under REACH.^6^

Mixing could potentially lead either to formulations that have properties outside the ranges defined by the pure components, or, if mixing is close to ideal, could allow precise fine-tuning of properties within the boundaries imposed by the simple ionic liquids. Either outcome would carry potential economic and scientific benefits. In this paper we report the results of a study designed to determine which of these situations arises when common ionic liquids are mixed.

A *constituent* of a mixture refers to any chemical species present in the mixture; a *component* is a thermodynamically independent constituent. In an aqueous solution of sodium chloride, water, sodium and chloride are all constituents of the solution, but water and sodium chloride (because the concentn of the two ions is inseparable) are the components of the solution. An ion on its own cannot be a component.

In this contribution, we adopt a nomenclature for naming ionic liquid mixtures based upon the number of components in the product mixture. Hence, [A]_*a*_[B]_*b*_[X] and [A][X]_*x*_[Y]_*y*_ (where *a*, *b*, *x* and *y* are the mole fractions, ‘*χ*’, of [A], [B], [X] and [Y], respectively) are *binary* (two-component) mixtures and [A]_*a*_[B]_*b*_[X]_*x*_[Y]_*y*_ (where *a* = *x* and *b* = *y*) are *reciprocal* binary mixtures.

**Fig. 1:**
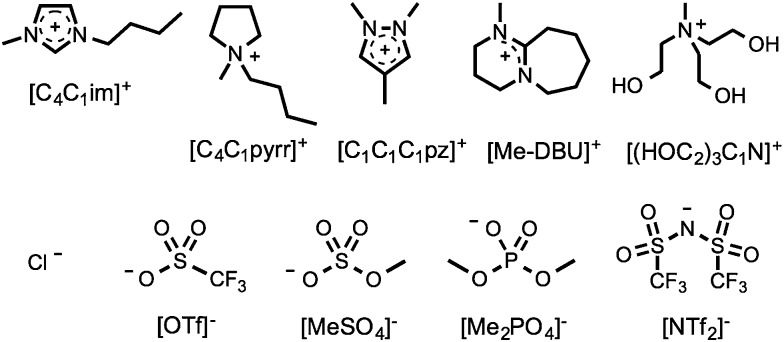
Structures and abbreviations of the ionic liquid cations and anions employed in this investigation.

To understand the influence of mixing on various physical and chemical parameters of ionic liquids, a series of binary and reciprocal binary ionic liquid mixtures were prepared and analysed. The incorporated cations and anions were selected in order to cover a wide range of commonly encountered species, including imidazolium, [C_*n*_C_*m*_im]^+^, pyrrolidinium, [C_*n*_C_*m*_pyrr]^+^, pyrazolium, [C_*n*_C_*m*_C_*p*_pz]^+^, and diazabicycloundecenium-derived, [R-DBU], ionic liquids. The investigated cations and anions are shown in Fig. 1. A mixture series is the set of ionic liquid mixtures created by altering the mole fraction, *χ*, of the components. The mixture series investigated in this contribution are listed in Table 1.

**Table 1:** Ionic liquid binary (**1–9**) and reciprocal binary (**10** and **11**) mixture series investigated in this study

Ionic liquid mixture	Component ILs
**1** [C_4_C_1_im]Cl[OTf]	[C_4_C_1_im]Cl, [C_4_C_1_im][OTf]
**2** [C_4_C_1_im][MeSO_4_][Me_2_PO_4_]	[C_4_C_1_im][MeSO_4_], [C_4_C_1_im][Me_2_PO_4_]
**3** [C_4_C_1_im][OTf][NTf_2_]	[C_4_C_1_im][OTf], [C_4_C_1_im][NTf_2_]
**4** [C_4_C_1_im][(HOC_2_)_3_C_1_N][MeSO_4_]	[C_4_C_1_im][MeSO_4_], [(HOC_2_)_3_C_1_N][MeSO_4_]
**5** [C_4_C_1_im][MeSO_4_][NTf_2_]	[C_4_C_1_im][MeSO_4_], [C_4_C_1_im][NTf_2_]
**6** [C_4_C_1_pyrr][NTf_2_][Me_2_PO_4_]	[C_4_C_1_pyrr][NTf_2_], [C_4_C_1_pyrr][Me_2_PO_4_]
**7** [C_4_C_1_im][NTf_2_][Me_2_PO_4_]	[C_4_C_1_im][NTf_2_], [C_4_C_1_im][Me_2_PO_4_]
**8** [C_4_C_1_im][Me-DBU][MeSO_4_]	[C_4_C_1_im][MeSO_4_], [Me-DBU][MeSO_4_]
**9** [C_4_C_1_im][C_1_C_1_C_1_pz][OTf]	[C_4_C_1_im][OTf], [C_1_C_1_C_1_pz][OTf]
**10** [C_4_C_1_im][Me-DBU][MeSO_4_][NTf_2_]	[C_4_C_1_im][NTf_2_], [Me-DBU][MeSO_4_]
**11** [C_4_C_1_im][C_4_C_1_pyrr][OTf][NTf_2_]	[C_4_C_1_im][OTf], [C_4_C_1_pyrr][NTf_2_]

Several recent publications have focused on attaining an understanding of the physical properties of ionic liquid mixtures. Foremost, the density and excess molar volume has been determined for mixtures of common ionic liquid cations and anions. Canongia Lopes *et al.* conducted an extensive early study into binary mixtures incorporating [C_*n*_C_1_im]^+^, [NTf_2_]^–^, [PF_6_]^–^ and [BF_4_]^–^ ions.^11^ The resultant mixtures were found to broadly obey the ideal mixing law; where exceptions arose, excess molar volumes were small and positive, indicating an absence of new strong interactions in the mixture. Indeed, such a trend is typically observed across the ionic liquid mixtures literature,^11–15^ although specific examples of negative excess molar volumes (*e.g.* binary mixtures of [C_3_(C_1_)^3^py][N(CN)_2_][BF_4_]),^12,13^ and of ordered nanostructures,^16^ are known.

Similarly, conductivity measurements exhibited either expected behaviour upon mixing,^17^ or minor deviation above^14,18^ or below^19,20^ the anticipated values. Occasionally, mixtures were observed to deviate more substantially from the anticipated behaviour, *e.g.*, the binary mixture series [C_2_C_1_im][N(CN)_2_][BF_4_].^14^ However, despite this deviation, conductivity values for the mixtures did fall within the boundaries dictated by the component ionic liquids.

Whilst studies of this type have yielded valuable data regarding the physical properties of ionic liquid mixtures, the investigations have generally focused on individual mixtures, or on a limited array of cations and anions, and each employing only a restricted group of techniques.

In this study, we thoroughly investigate a broad series of ionic liquid binary and reciprocal binary mixtures with respect to physical properties of density, viscosity, phase behaviour and conductivity. The incorporated cations and anions were selected in order to cover a wide range of commonly encountered species, varying in size, polarity and asymmetry. This has allowed us to provide key insights as to which properties of the ions control the extent of deviation from ideal mixing for ionic liquids for the first time.

## Experimental

Syntheses of the simple ionic liquids and the precise mole fractions, (*χ* = 0–1), of the mixtures are described in the ESI.† Prior to each measurement, all samples were dried under vacuum until a water content of <150 ppm (w/w) was established *via* Karl Fischer titn.

Density measurements were performed in an Anton Paar ‘DMA38’ vibrating tube density meter at 25 °C. The error of the instrument given by the manufacturer was ±0.001 g cm^–3^, although reproducibility was one order of magnitude better.

Differential Scanning Calorimetry (DSC) results were obtained using aluminium sample pans of diameter 7 mm. Between 7–9 mg of the ionic liquid was measured into the aluminium sample pan. A small incision was made in the tops of both the sample and reference pans. A drying procedure was then implemented: the sample pan was heated to 100 °C for 20 minutes in the calorimeter, to remove water. The sample pan was then quickly re-weighed, in order to determine the dry weight of the ionic liquid. The sample was measured between –100 and +100 °C, with a heating/cooling rate of 20 °C min^–1^.

Viscosity measurements were performed on a TA instruments ‘AR2000ex’ rheometer fitted with a Peltier plate at 25 °C, using a 40 mm, 2° steel cone. Measurements were performed at an angular velocity between 0.1 and 10 rad s^–1^ under a nitrogen atmosphere. An error of 5% was established from the manufacturer's information and measurements on standard liquids, although reproducibility was usually better.

Conductivity measurements were performed using a home-built conductivity probe with two platinum paddles of size approximately 5 mm × 5 mm. The temperature was maintained at 25 °C using a water bath in a jacketed beaker attached to a recirculator, and the samples were allowed to reach thermal equilibrium for 30 minutes. The measurements were performed under an inert argon atmosphere on a CH Instruments CHI760C apparatus in a frequency range from 1 Hz to 1 × 10^5^ Hz, and at an amplitude of 1 × 10^–4^ V. Prior to each set of measurements the probe was calibrated using three commercially available standards (HANNA instruments) with known conductivities of 84 μS cm^–1^, 1413 μS cm^–1^ and 12 880 μS cm^–1^. The average error was found to be 2%.

Temperature-ramped Thermogravimetric Analysis (TGA) experiments were performed on a PerkinElmer ‘Pyris 1 TGA’ thermogravimetric analyzer, and platinum sample pans of 6 mm diameter. Experiments were carried out in the range 120–600 °C. Between 3 and 36 mg of the ionic liquid was measured into the platinum pan. A ramping rate of 10 °C min^–1^ and a nitrogen flow of 20 ml min^–1^ were employed for all experiments.

## Results and discussion

Mixing of the cations and anions in Fig. 1 yields, in the most part, binary and reciprocal binary ionic liquid mixtures that exhibit ideal or close to ideal behaviour with respect to the measured physical properties.

One notable exception is the binary mixture series [C_4_C_1_im][NTf_2_][Me_2_PO_4_], **7**, which exhibits a small, but consistent deviation from an ideal relationship between the mole fraction of the component ionic liquids, *χ*, and the viscosity, glass transition temperature, conductivity and molar volume. This non-ideal behaviour was also observed when the [C_4_C_1_im]^+^ cation was replaced by [C_4_C_1_pyrr]^+^, in the case of mixture series [C_4_C_1_pyrr][NTf_2_][Me_2_PO_4_], **6**.

The thermal stability behaviour of the investigated ionic liquid mixtures could be best explained in terms of the stabilities of the component ionic liquids; Thermogravimetric Analysis (TGA) experiments of mixtures mostly exhibit two or more distinct thermal decomposition steps, corresponding to these component ionic liquids. Little or no cooperative behaviour could be observed from the temperature-ramped TGA experiments.

Results for separate physical and chemical properties (molar volumes, viscosity, conductivity, phase behaviour and thermal stability) are discussed individually below. Data for selected ionic liquid mixture series are shown in graphical form, chosen in order to represent at least one example of an ideal/linear mixture series and a non-ideal/non-linear mixture series in each circumstance. All other graphical data can be found in the ESI.†

### Density/molar volume

**Fig. 2:**
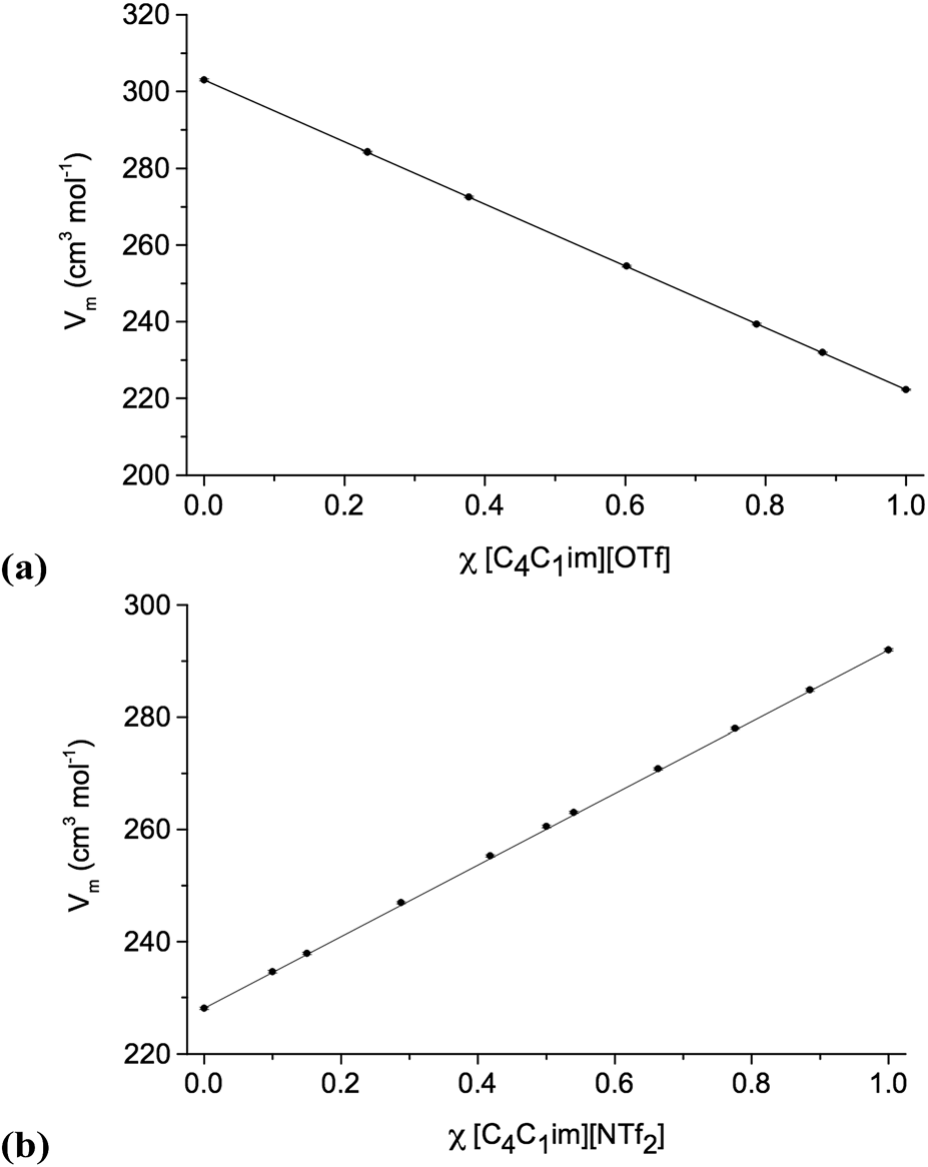
Molar volumes: (a) [C_4_C_1_im][C_4_C_1_pyrr][OTf][NTf_2_], **11**; (b) [C_4_C_1_im][NTf_2_][Me_2_PO_4_], **7**. Straight lines, connecting points *χ* = 0 and *χ* = 1, represent ideal behaviour.

Density measurements were carried out for all ionic liquid mixture series **1–11** (Table 1), with varying mole fraction, *χ*. The densities of [C_4_C_1_im][NTf_2_][Me_2_PO_4_], **7** (exhibiting non-ideal behaviour) and [C_4_C_1_im][C_4_C_1_pyrr][OTf][NTf_2_], **11** (showing ideal behaviour) are shown in Fig. 2. Graphs for the other investigated ionic liquid mixture series, and numerical data, can be found in the ESI (Fig. E2, Table E2†).

Due to the temperature restrictions of the apparatus, it was not possible to obtain density data for mixtures or simple ionic liquids that were solid at room temperature. In these circumstances, the available data were extrapolated and the corresponding values are given in parenthesis in the ESI (Table E2†).

The density results indicate that most of the investigated mixture series show clear linear behaviour within the precision of the instrument (±0.001 g cm^–3^). The molar volume of a mixture is directly related to the chemical potentials of the components of the mixture and so shows linear dependence on the mole fractions of the pure components when ideal mixing occurs. Hence, these can be described as ideal mixtures.

Three mixture series do deviate systematically from linear behaviour, specifically [C_4_C_1_im][MeSO_4_][NTf_2_], **5**, [C_4_C_1_im][NTf_2_][Me_2_PO_4_], **7**, and reciprocal binary mixtures of [C_4_C_1_im][Me-DBU][MeSO_4_][NTf_2_], **10**. As the density of [Me-DBU][MeSO_4_], a room-temperature solid, could not be measured directly, it was determined by linear extrapolation from the data of the mixtures of **5**. The corresponding data should therefore be treated with caution.

To quantify the deviation from ideal behaviour for the above-mentioned ionic liquid mixtures, the excess volume Δ*V*_m_ was calculated:

1 Δ*V*_m_ = *V*_m,exp_ – *V*_m,lin_

**Fig. 3:**
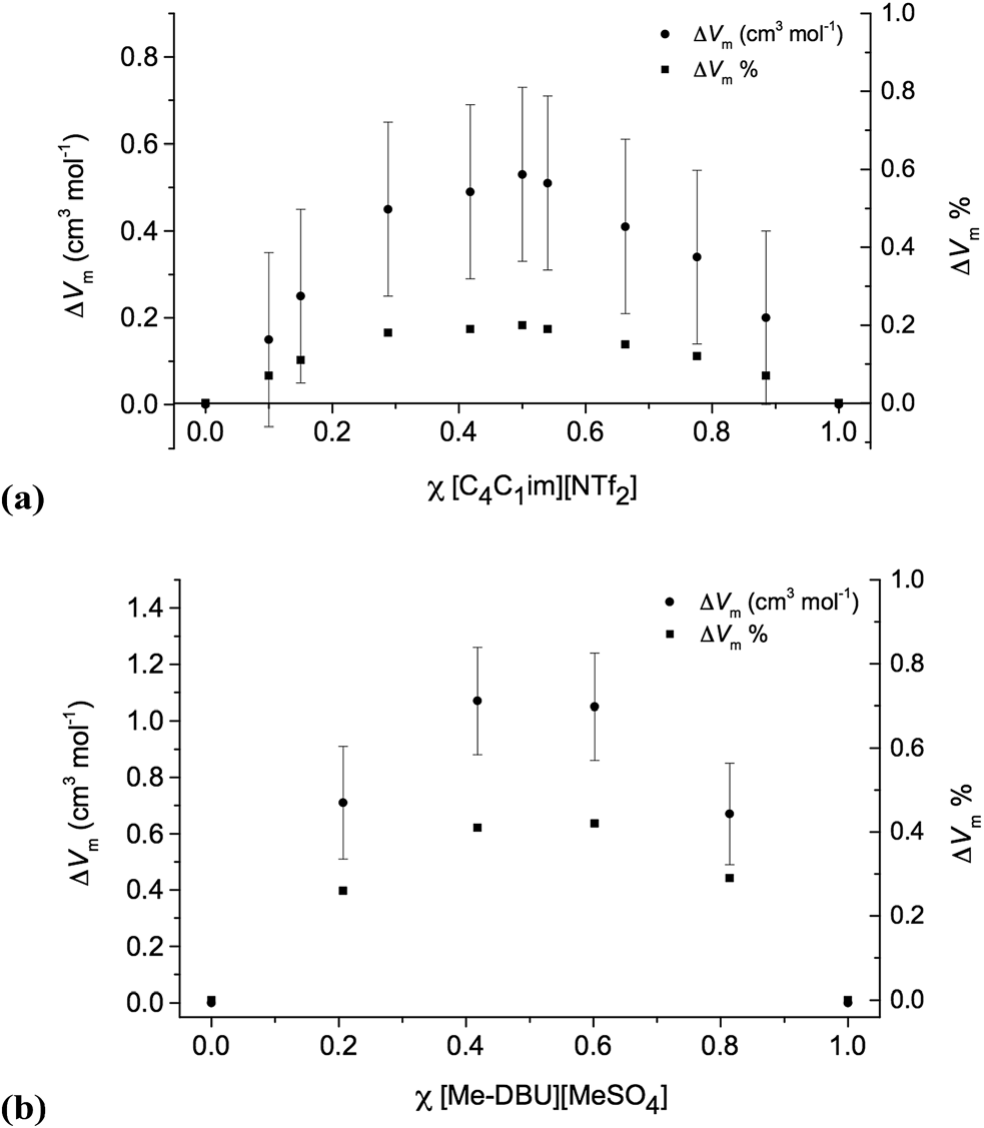
Excess molar volumes, represented as absolute values, cm^3^ mol^–1^, and as % relative to the expected molar volume: (a) [C_4_C_1_im][Me_2_PO_4_][NTf_2_], **7**; (b) [C_4_C_1_im][Me-DBU][MeSO_4_][NTf_2_], **10**.

The corresponding data is shown for ionic liquid mixture series **7** and **10** below (Fig. 3), and for **5** in the ESI (Fig. E3†).

For the mixture series **5**, **7** and **10**, the molar volume is larger than expected (excess molar volume) for ideal (linear) mixing. This suggests that there is more free space in these mixtures and the sum of favourable interactions is fewer than expected. These mixtures incorporate a highly-fluorinated anion, [NTf_2_]^–^, and another anion with no fluorine atoms, [MeSO_4_]^–^ or [Me_2_PO_4_]^–^. Hence, one possible explanation is that a loss of fluorophilic interactions is occurring, causing a decrease in density. However, the similarity of these results to those of Canongia Lopes *et al.* for binary mixtures of [C_*n*_C_1_im][C_*m*_C_1_im][NTf_2_], [C_4_C_1_im][PF_6_][NTf_2_], [C_4_C_1_im][BF_4_][NTf_2_] and [C_4_C_1_im][BF_4_][PF_6_], all of which have only fluorinated anions, suggest that this is unlikely to be the explanation for our results.^11^

It has also previously been suggested that the size differences between ions, and thus packing effects, may be important. Navia *et al.* suggested, on the basis of ionic liquid mixture series [C_4_C_1_im][BF_4_][PF_6_] and [C_4_C_1_im][BF_4_][MeSO_4_], that differences in the amount of free space, driven by differences in the sizes of ions, in the pure components could lead to the *negative* Δ*V*_m_ values that they observed.^12^ Another possible explanation is that disruption of the cation–anion hydrogen bonding in these mixtures could lead to the observed deviation from ideal behaviour. Explanations for the small deviations from ideal behaviour of ionic liquid mixture series **5**, **6**, **7**, **8** and **10** are discussed more fully below.

Overall, the most striking feature of the mixture series studied here, and others that have been reported in the literature^21^ is that, with respect to molar volume, their mixing is remarkably close to ideal. Even for the greatest deviations found, the maximum value of Δ*V*_m_ was <0.5% (relative to the expected molar volume) for the reciprocal binary mixture series **10**, and were smaller still (<0.35%) for the two binary mixture series **5** and **7**.

It is, perhaps, unsurprising that binary mixture series **5** and **7** exhibit notably smaller percentage deviations than the reciprocal binary mixtures of **10**. For the *binary* mixtures incorporating, for example, a common cation, chemically distinct anions are likely to be surrounded by a solvation sphere of the common cation as they are in the simple ionic liquids, and therefore the major changes occur in their second solvation sphere where anion–anion interactions will be both long range and weak. These relatively small structural changes would also lead to the corresponding entropy of mixing, Δ*S*_mix_ to be small. In the case of *reciprocal* binary mixtures, the first solvation spheres of all of the constituent ions liquid are disrupted in comparison to the simple ionic liquids by the introduction of new cations *and* anions. As well as giving rise to the possibility of larger values of Δ*H*_mix_, Δ*S*_mix_ will also be more substantial. The other reciprocal binary mixture series investigated in this study, **11**, involves the pairing of far more chemically similar ions than **10**, which will partially offset this effect. Precise properties of the ions that influence non-ideality are examined more thoroughly below.

### Phase behaviour

It is well established that many ionic liquids are glass-transforming materials,^22^ and therefore do not exhibit a clear freezing point. Introducing additional ionic liquid species generates a further likelihood that no crystalline phase will be observed.^23^ For this reason, full phase diagrams are sometimes difficult to obtain. Inorganic salt mixtures often exhibit eutectic behaviour.^24,25^

A recent example of a binary ionic liquid mixture series in the literature, [C_2_C_1_im][C_4_C_1_pyrr][B(CN)_4_], demonstrated that the glass transition temperature (*T*_g_) of the mixture may behave as a weighted average of the simple ionic liquid components.^26^ Every *et al.* observed for mixtures of [C_2_C_1_im][OTf] and [C_2_C_1_im][NTf_2_] that the value of *T*_g_ was a weighted average of the two simple ionic liquids, although *T*_g_ values for the simple ionic liquids were only 5 °C apart.^18^

**Fig. 4:**
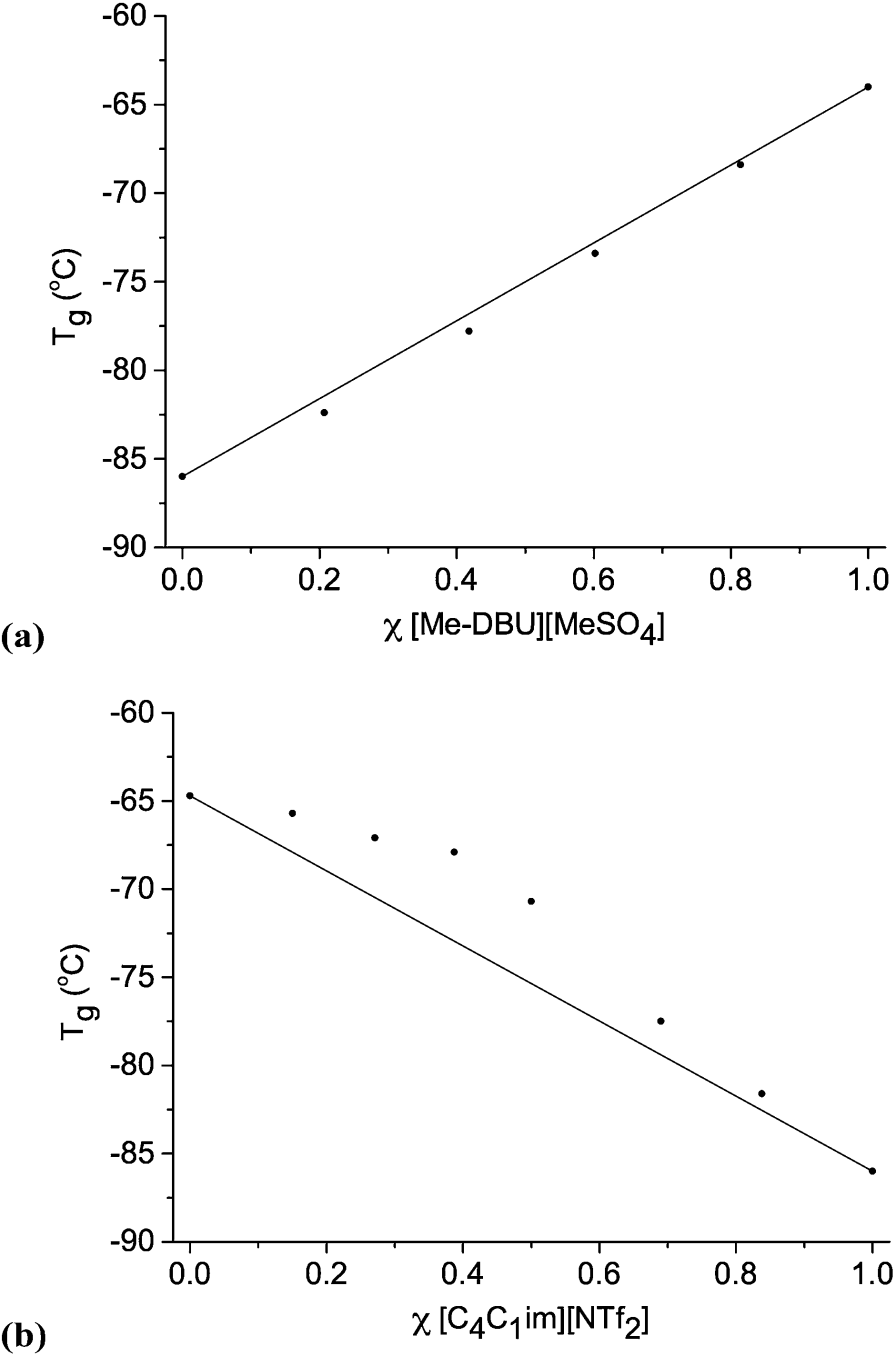
Glass transition temperatures (*T*_g_) for ionic liquid mixture series: (a) [C_4_C_1_im][Me-DBU][MeSO_4_][NTf_2_], **10**; (b) [C_4_C_1_im][NTf_2_][Me_2_PO_4_], **7**.

We employed Differential Scanning Calorimetry (DSC) to elucidate the phase behaviour of ionic liquid mixture series, **1–11**. A value of *T*_g_ was measured for each ionic liquid mixture. The DSC conditions are described fully in the experimental section and in the ESI.† Graphs demonstrating the change in *T*_g_ with changing mole fraction, *χ*, for selected ionic liquid binary and reciprocal binary mixture series are displayed in Fig. 4. Graphs for the other mixture series, tables of numerical data, and one example of a DSC trace are shown in the ESI (Fig. E4, Table E4†).

For the majority of ionic liquid mixture series in this study, DSC data reveals that the change in *T*_g_ with changing mole fraction is close to linear. This is most apparent for those mixture series where the *T*_g_ values of the simple ionic liquids are far apart, for example mixtures of [C_4_C_1_im][Me-DBU][MeSO_4_][NTf_2_], **10** (Fig. 4a).

Two binary ionic liquid mixture series exhibit clear non-linear behaviour with respect to *T*_g_, [C_4_C_1_pyrr][NTf_2_][Me_2_PO_4_], **6**, and [C_4_C_1_im][NTf_2_][Me_2_PO_4_], **7**. The *T*_g_ values are larger than expected, but deviate in a reasonably smooth fashion, with no discontinuities. For mixture series **7**, the largest outlying point (*χ* [NTf_2_]^–^ = 0.387) deviates from the expected value of *T*_g_ by ∼5 °C.

Several of the simple ionic liquids, and the majority of the ionic liquid mixtures in this investigation, did not exhibit a clear melting point in the accessible temperature range (–100 to +100 °C) of the DSC apparatus. Therefore, the DSC experiments were unable to determine whether the melting points (*T*_m_) for the ionic liquid mixtures behave in an ideal or non-ideal manner. Nevertheless, the DSC experiments demonstrate the linear or close to linear relationship between *T*_g_ and *χ* for the majority of the investigated ionic liquid mixture series, **1–11**. The greatest deviation from linear behaviour was for the two binary ionic liquid mixture series that contain both the [NTf_2_]^–^ and [Me_2_PO_4_]^–^ anions, **6** and **7**. Series **7** also showed substantial deviation from ideal mixing in the molar volume data.

### Viscosity

**Fig. 5:**
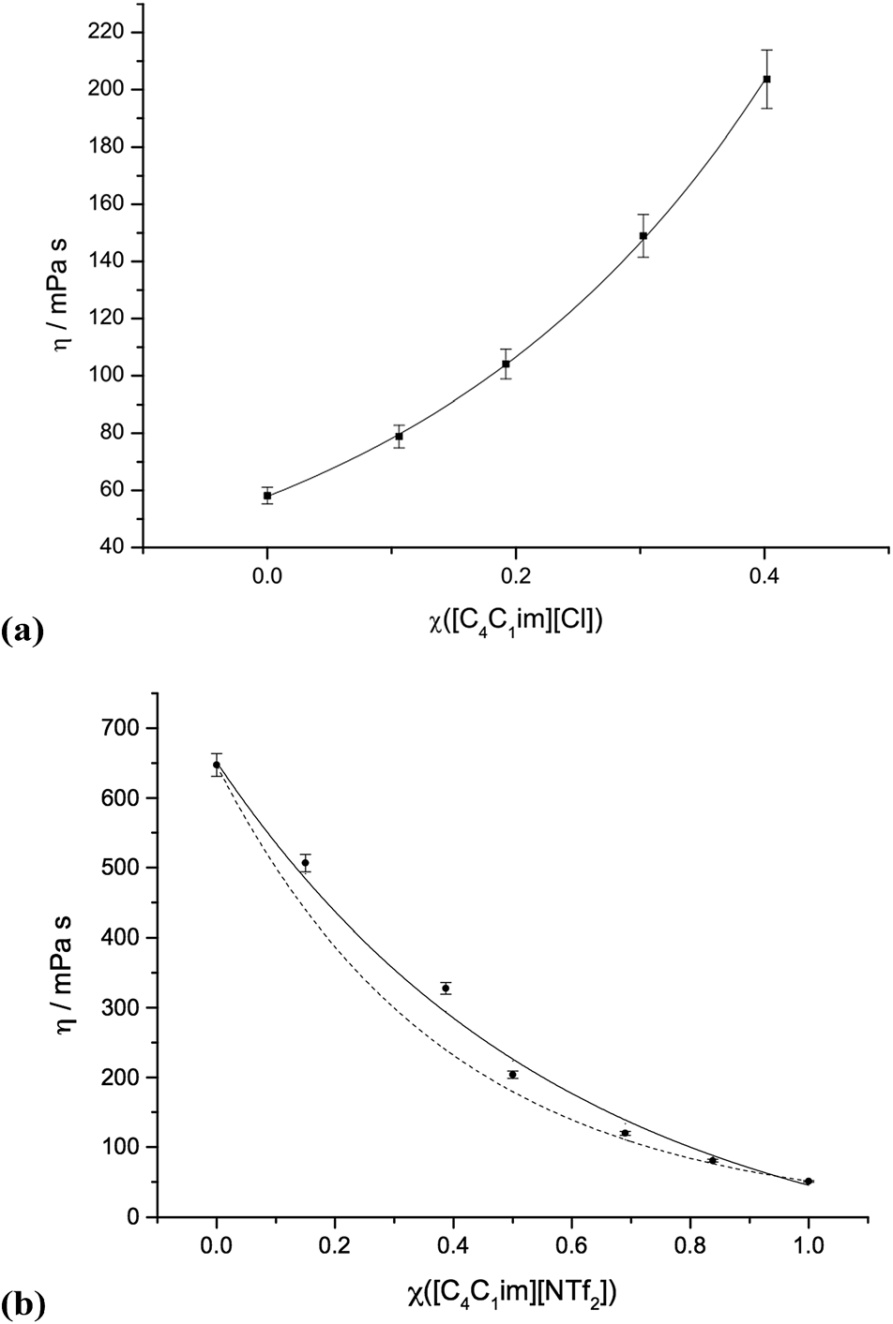
Viscosities of mixture series: (a) [C_4_C_1_im]Cl[OTf], **1**; (b) [C_4_C_1_im][NTf_2_][Me_2_PO_4_], **7**. The dashed line in (b) represents the ideal Katti and Chaudhri mixing law (eqn (2)), continuous lines include the additional ‘*f*’ non-ideality parameter of Grunberg and Nissan (eqn (3)).

Graphs of the viscosity data for selected ionic liquid binary mixture series are presented in Fig. 5. Graphs for the other mixture series are shown in the ESI (Fig. E5†). The corresponding numerical data, including non-ideality parameters ‘*f*’, is listed in the ESI (Table E5†).

Unlike densities, viscosities do not necessarily vary linearly with the mole fraction of the components of a mixture, even when ideal mixing is occurring. Instead, two plausible correlation functions are shown below. The first is the ideal Katti and Chaudhri mixing law under the assumption of perfectly linear mixing with *χ* (shown as a dashed line in Fig. 5 and E5†):

2 log *ηV*_m_ = *χ* log *η*_1_*V*_m,1_ + (1 – *χ*)log *η*_2_*V*_m,2_

The viscosity data shows good agreement with the Katti and Chaudhri law^27^ for certain mixture series; [C_4_C_1_im][OTf][NTf_2_], **3**, [C_4_C_1_im][C_1_C_1_C_1_pz][OTf], **9**, and [C_4_C_1_im][C_4_C_1_pyrr][OTf][NTf_2_], **11**. These represent mixtures where overall changes in viscosity are small, thus errors in viscosity measurements are comparatively high, giving the impression of close adherence to ideal mixing law. For all other mixtures, some deviation towards either higher or lower viscosities was observed.

To quantify the non-ideality of the mixture series, a variation of the Grunberg and Nissan mixing law was employed,^28^ which includes an additional parameter, *f*, to account for non-ideality:

3 log *η* = *χ* log *η*_1_ + (1 – *χ*)log *η*_2_ + *χ*(1– *χ*)(*f*/*RT*)

Eqn (3) was optimised for all mixture series, and is shown as a continuous line in the corresponding graphs (Fig. 5 and E5†).

The additional ‘*f*’ parameter (ESI Table E5†) allows near quantitative fitting for seven of the eight mixture series that were not well described by the Katti and Chaudhri law (**1**, **2**, **4**, **5**, **6**, **8** and **10**). However, binary ionic liquid mixtures series [C_4_C_1_im][NTf_2_][Me_2_PO_4_], **7**, still displays noticeable deviation despite the inclusion of *f*.

Therefore, 10 of the 11 studied mixture series can be described by either Katti and Chaudhri or Grunberg and Nissan mixing laws. Those only described by Grunberg and Nissan mixing law cannot be regarded as adhering to ideality, because of the inclusion of the *f* parameter.

[C_4_C_1_im][NTf_2_][Me_2_PO_4_], **7**, is a notable exception. Thus, the molar volume, *V*_m_, glass transition temperature, *T*_g_, and viscosity, *η*, measurements for this series have all shown non-linear behaviour. It appears, therefore, that the structural and physical properties of each of the simple ionic liquids, [C_4_C_1_im][NTf_2_] and [C_4_C_1_im][Me_2_PO_4_] are disrupted upon the inclusion of ions from the other component. However, similarly to the density and glass transition temperature experiments, the absolute deviations from ideal viscosity are very small. Thus, within a very reasonable error, it remains possible to predict and obtain the desired property (within the boundaries set by the simple ionic liquids).

### Conductivity

The conductivity, measured by impedance spectroscopy, is referred to as *Λ*_m,Imp_. The conductivity was also calculated from the self-diffusion constants using the Nernst–Einstein equation, and is presented as *Λ*_m,NMR_. The ‘Haven ’, *R*_H_, is defined as the between the measured conductivity (*Λ*_m,Imp_) and the conductivity calculated from diffusion coefficients (*Λ*_m,NMR_), and should be equal to one in a system of fully dissociated and independent ions (eqn (4)).

4 *R*_H_ = *Λ*_m,Imp_/*Λ*_m,NMR_

The conductivity is not directly related to Raoult's law, and therefore does not necessarily scale linearly with the mole fraction, *χ*. Instead, a logarithmic relationship between the conductivity and *χ* has previously been identified as a likely outcome for ionic liquids.^18^ Therefore, each of the two conductivity curves (*Λ*_m,Imp_ and *Λ*_m,NMR_) were fitted by a logarithmic eqn (5):

5 log *Λ* = *χ* log *Λ*_1_ + (1 – *χ*)log *Λ*_2_ + *χ*(1 – *χ*)(*f*/*RT*)

**Fig. 6:**
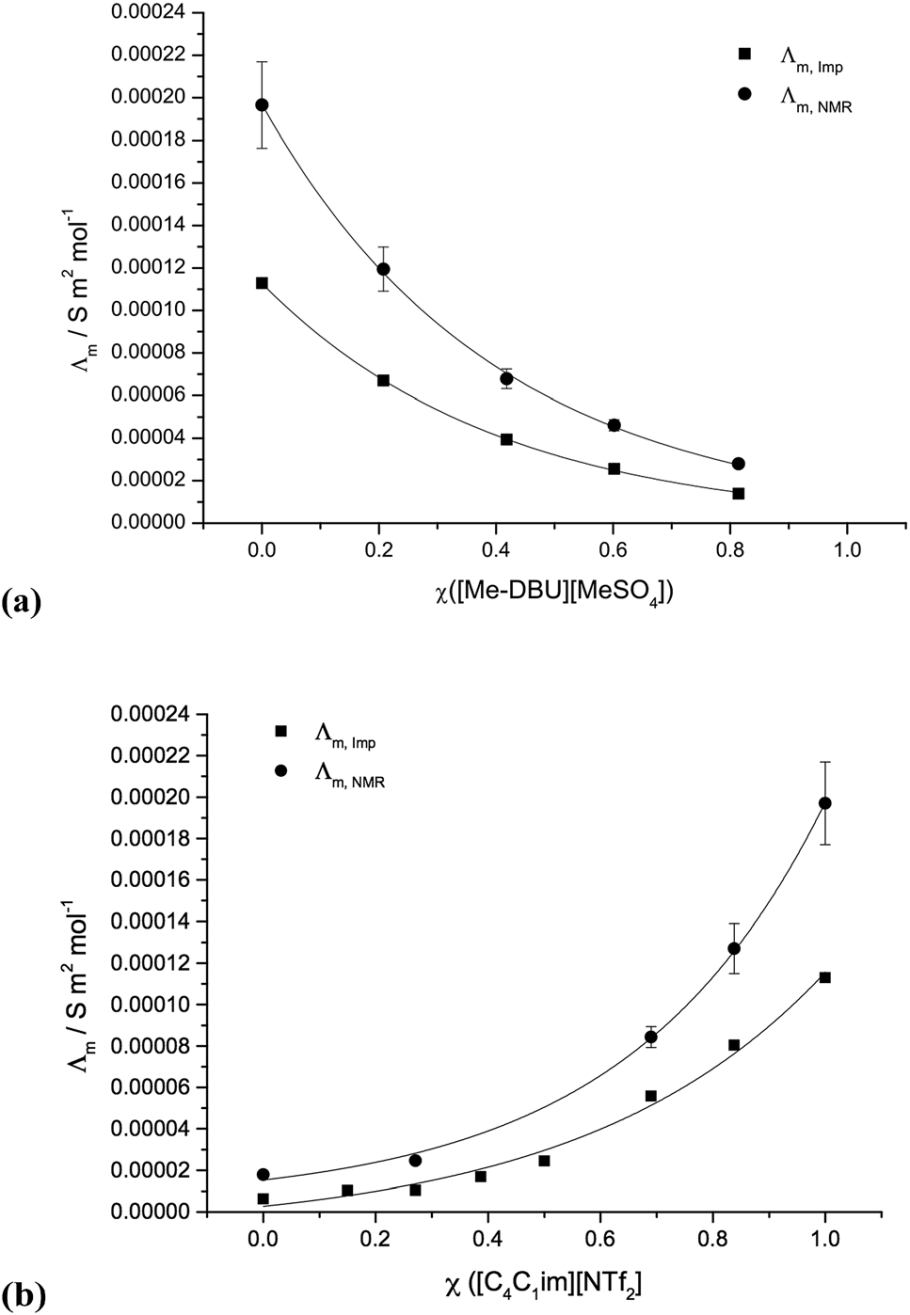
Conductivities of binary and reciprocal binary ionic liquid mixture series, both from impedance spectroscopy, *Λ*_m,Imp_, and from the self-diffusion coefficients, *Λ*_m,NMR_: (a) [C_4_C_1_im][Me-DBU][MeSO_4_][NTf_2_], **10**; (b) [C_4_C_1_im][NTf_2_][Me_2_PO_4_], **7**.

Conductivity graphs for [C_4_C_1_im][NTf_2_][Me_2_PO_4_], **7** and [C_4_C_1_im][Me-DBU][MeSO_4_][NTf_2_], **10**, are displayed in Fig. 6. Graphs for other studied mixture series and the corresponding numerical data, including the non-ideality parameter ‘*f*’, is shown in the ESI (Fig. E6, Table E5†).

With the inclusion of the *f* parameter, the conductivity data can be well fitted for the majority of ionic liquid mixture series investigated; all values fall within or close to within the error. The reciprocal binary mixture [C_4_C_1_im][C_4_C_1_pyrr][OTf][NTf_2_], **11**, exhibits the worst fit for the conductivity measured from diffusion coefficients, *Λ*_m,Imp_ (Fig. E6b†). However, the differences in measured conductivities of the two simple ionic liquids, [C_4_C_1_im][OTf] and [C_4_C_1_pyrr][NTf_2_], are relatively small (Δ*Λ*_m,Imp_ = 2.0 × 10^–5^ S m^2^ mol^–1^, Δ*Λ*_m,NMR_ = 1.0 × 10^–5^ S m^2^ mol^–1^). Therefore, even proportionally large deviations from a logarithmic curve amount to very small deviations from the expected conductivity for any ionic liquid mixture.

In contrast to the molar volume, glass transition temperature and viscosity studies, the two binary mixture series incorporating both the [NTf_2_]^–^ and [Me_2_PO_4_]^–^ anions, **6** and **7**, demonstrate a reasonable logarithmic correlation between the conductivity and mole fraction.

**Fig. 7:**
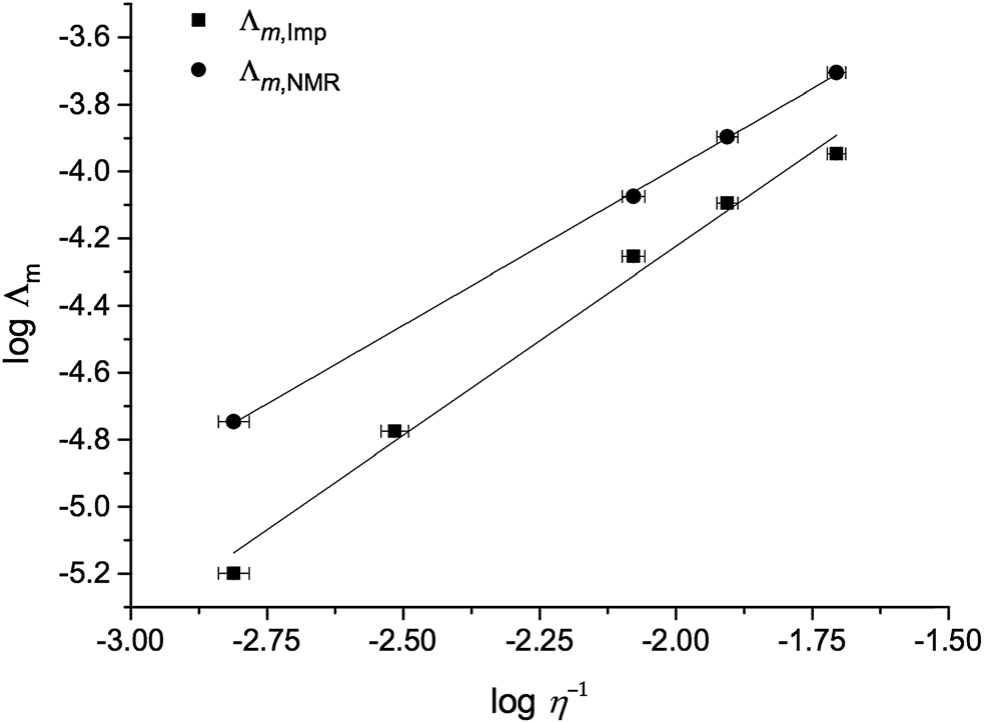
A Walden plot for the binary ionic liquid mixture series [C_4_C_1_im][NTf_2_][Me_2_PO_4_], **7**.

A Walden plot representing the relationship between the log of viscosity and log of conductivity for [C_4_C_1_im][NTf_2_][Me_2_PO_4_], **7**, is shown in Fig. 7 and in the ESI for all other mixture series (Fig. E7†).^29^

Considering the Stokes–Einstein and Nernst–Einstein equations, one would expect that a lower viscosity causes a higher conductivity, and *vice versa*. Therefore the non-ideality parameters, ‘*f*’, for the two conductivities are expected to always be of opposite sign relative to the corresponding calculated viscosity parameter. However as demonstrated by the tabulated *f* values in the ESI (Table E5†), this is not the case. There appears to be a general tendency for the bulk conductivities from impedance spectroscopy, *Λ*_m,Imp_, to be higher than ideal, while those calculated from self-diffusion constants, *Λ*_m,NMR_, tend to be lower. However, no linear or other correlation between *f*_visc_ and *f*_Imp_ or *f*_NMR_ is apparent. It must, therefore, remain unclear whether a relationship exists.

The overall deviations from ideal behaviour for both viscosity and conductivity are relatively small and the anticipated linear relationship between log *Λ*_m_ and log *η* ^–1^ holds well, with the exception of the more strongly deviating mixture series, most notably [C_4_C_1_im][NTf_2_][Me_2_PO_4_], **7** (Fig. 7).

Overall, the majority of mixture series investigated, **1–11**, exhibit either ideal or close to ideal conductivity with respect to both the measured and the calculated values (*Λ*_m,Imp_ and *Λ*_m,NMR_, respectively). Moreover, a linear or fairly close to linear relationship between log *Λ*_m_ and log *η* ^–1^ is established for all the investigated ionic liquid mixture series. Even for the mixture series that exhibited the largest deviation from expected behaviour, notably **11**, absolute differences in *Λ*_m,Imp_ and *Λ*_m,NMR_ are minor. Therefore, ionic liquid mixtures can be expected to behave systematically with respect to conductivity; data points are fitted well by a logarithmic curve.

### Thermal stability

Thermogravimetric Analysis (TGA) has frequently been used to investigate the thermal stability of simple ionic liquids.^30–36^ The *T*_onset_ parameter, acquired by employing a step-tangent method, is often used to quantify thermal stabilities of ionic liquid compounds.^36–38^ This method suffers from an overestimation of long-term thermal stabilities,^36,39,40^ however, the *T*_onset_ parameter is beneficial when *comparing* thermal stabilities of like compounds. This assumes that care is taken to keep experimental conditions uniform.^41^ However, the thermal decomposition behaviour of ionic liquid mixtures has only been partially studied.^42^

Thermal decomposition is a chemical property and not a physical property. Thermal stability experiments can demonstrate whether individual components in a mixture decompose separately, with similar *T*_onset_ values to those of the neat ionic liquids. Alternatively, cooperative thermal decomposition behaviour may occur.

**Fig. 8:**
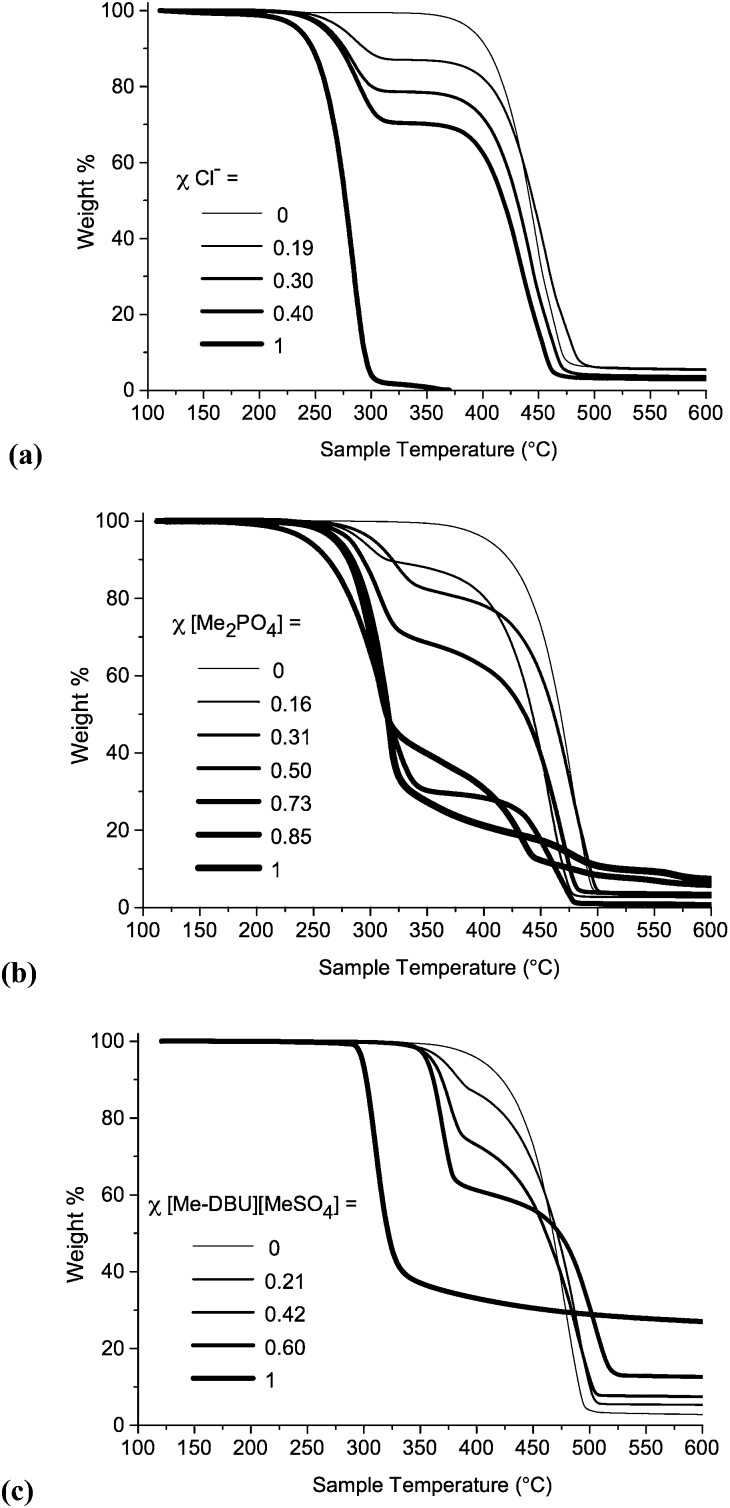
Temperature-ramped TGA thermographs for ionic liquid mixtures: (a) [C_4_C_1_im]Cl[OTf], **1**; (b) [C_4_C_1_im][NTf_2_][Me_2_PO_4_], **7**; (c) [C_4_C_1_im][Me-DBU][MeSO_4_][NTf_2_], **10**.

TGA experiments were performed for all ionic liquid mixtures in this investigation. TGA thermographs for ionic liquid mixture series **1**, **7** and **10** are displayed below in Fig. 8. TGA thermographs for the other investigated mixture series are shown in the ESI (Fig. E8†). *T*_onset_ decomposition temperatures for investigated ionic liquids and mixtures are listed in the ESI (Table E6†).

For the binary ionic liquid mixture series in this study the temperature-ramped TGA experiments reveal that no significant stabilizing or de-stabilizing effect is conferred on the constituent ions by the incorpon of additional constituents into a mixture. Instead, TGA thermographs for the mixtures display two distinct steps. This phenomenon is particularly pronounced for the mixtures where the *T*_onset_ temperatures of the component ionic liquids are substantially different, for example in the case of [C_4_C_1_im]Cl[OTf], **1** (Δ*T*_onset_ = 155 °C), shown in Fig. 8a.

It has been noted that for ionic liquids incorporating the [NTf_2_]^–^ anion, vaporisation of the intact ionic liquid is likely to make a significant contribution to mass loss.^43–50^

Only two reciprocal binary ionic liquid mixture series were studied, [C_4_C_1_im][Me-DBU][MeSO_4_][NTf_2_], **10** (Fig. 8c), and [C_4_C_1_im][C_4_C_1_pyrr][OTf][NTf_2_], **11**. It is plausible that for particular reciprocal binary mixtures, the *T*_onset_ value of the mixture could be lower than that of either component ionic liquid; decomposition between the cation of component 1 and the anion of component 2, or *vice versa*, could occur at a lower temperature.

The TGA thermograph for [Me-DBU][MeSO_4_] (Fig. 8c) does not reach a 0% weight, and a large quantity of residue is still present in the platinum pan at 600 °C. The quantity of residue in mixtures of **10** is proportional to the mole fraction of [Me-DBU][MeSO_4_]. Moreover, the stability of [Me-DBU][MeSO_4_] appears to be substantially improved when incorporated into a mixture with [C_4_C_1_im][NTf_2_], even when the mole fraction of [Me-DBU][MeSO_4_] is high. This is demonstrated by the large difference in *T*_onset_ values when *χ*[Me-DBU][MeSO_4_] = 1 (*T*_onset_ = 297 °C) and *χ*[Me-DBU][MeSO_4_] = 0.60 (*T*_onset_ = 355 °C). A similar, but less pronounced, effect was observed for the binary mixtures of [C_4_C_1_im][Me-DBU][MeSO_4_], **8**. Although these are isolated and atypical examples, it nevertheless demonstrates that it might be possible to significantly raise the thermal stability of an ionic liquid while retaining beneficial properties for an intended application. The precise decomposition behaviour of an ionic liquid mixture is difficult to evaluate when the *T*_onset_ temperatures of the component ionic liquids are very similar, for example the mixture series incorporating both [OTf]^–^ and [NTf_2_]^–^ anions, **3** and **11**.

Therefore in summary, the temperature-ramped TGA experiments demonstrate that, in the majority of cases, the thermal stability of ionic liquid mixtures can be understood in terms of separate decomposition of the individual constituent ions. This, in agreement with previous literature, manifests as multiple weight loss steps.^42^ There were no observed examples where the thermal stability of a mixture was substantially *lower* (>30 °C) than that of the less stable component ionic liquid. This is likely to prove beneficial when selecting ionic liquids for high-temperature applications.

### nalisation of ionic liquid mixing behaviour

For each of the physical and chemical parameters investigated, the majority of the studied ionic liquid mixture series behaved in an ideal manner. Upon changing *χ* of the component ionic liquids, the molar volume, *V*_m_, glass transition temperature, *T*_g_, viscosity, *η*, and conductivity, *Λ*_m,Imp_ and *Λ*_m,NMR_, each exhibited the expected linear or logarithmic pattern, for the overwhelming majority of mixture series. Deviations from ideality are typically very small. This is in accord with the available literature.^11–15,17–20^

Ideality arises when Δ*H*_mix_ is equal to zero and mixing is entirely entropy driven. Navia *et al.* have shown Δ*H*_mix_ for [C_4_C_1_im][BF_4_][PF_6_] is small.^12^ This does not mean that all interactions in the mixture are the same as the interactions in the pure components, but rather that the sum of the interactions in the mixture are the same as the sum of the interactions in the pure components. We have previously shown on the basis of uv-vis spectroscopy of 1-ethyl-4-(methoxycarbonyl)pyridinium iodide in a variety of ionic liquids that this can be the case.^10^ This is, at least partly, due to the high degree of charge screening that is found in ionic liquids.^51–54^

In addition to this, we should recognise that the choice to study the mixing of *room temperature* ionic liquids has an important effect. Many room temperature ionic liquids have much in common, *e.g.*, containing ions having similar size, charge delocalisation and asymmetry. Hence, we have selected systems in which it is relatively likely that the condition, that the *sum* of the interactions in the pure components is close to the *sum* of the interactions in the mixtures, is met, although the strengths of individual interactions are likely to differ. Hence, the close to ideal mixing that has been observed for many ionic liquid mixtures is partly an expression of the similarities of many of the ions commonly used in ionic liquids.

Nevertheless, perfect ideality is rare and while similar in many ways, different ionic liquid ions are not identical. In our examples, the greatest behavioural range of adherence to ideal mixing occurred when mixtures containing different anions, rather than different cations, were formulated. Two binary ionic liquid mixture series with the [NTf_2_]^–^ and the [Me_2_PO_4_]^–^ anions (**6** and **7**) repeatedly demonstrated small, but systematic, deviations from the anticipated ideal behaviour. Ionic liquid mixture series with the [NTf_2_]^–^ and the [MeSO_4_]^–^ anions (**5** and **10**) showed somewhat lesser deviations, while the ionic liquid mixture series with [OTf]^–^ and Cl^–^ (**1**), [OTf]^–^ and [NTf_2_]^–^ (**3** and **11**) or [Me_2_PO_4_]^–^ and [MeSO_4_]^–^ anions (**2**) exhibited almost no deviation from ideal behaviour. Similar results have also been observed by others.^20^

In the following section we attempt to determine which properties are most significant in leading to non-ideal behaviour. Selected experimental and computational physical parameters, relating either to the component ionic liquids or the constituent cations and anions, have been tabulated (Tables 2–5, below). Data was acquired from the ionic liquid literature, or measured where required. All computational calculations were performed using DFT at the B3LYP/6-311+G(d,p) level of theory. Complete procedures are described in the ESI.†

**Table 2:** Differences in the molar volumes of the constituent cations, Δ*V*cationm, and anions, Δ*V*anionm, for ionic liquid mixture series **1–11** (in units of cm^3^ mol^–1^)

Ionic liquid mixture series	Δ*V*cationm	Δ*V*anionm
**1** [C_4_C_1_im]Cl[OTf]	—	38 ± 9
**2** [C_4_C_1_im][MeSO_4_][Me_2_PO_4_]	—	19 ± 6
**3** [C_4_C_1_im][OTf][NTf_2_]	—	59 ± 9
**4** [C_4_C_1_im][(HOC_2_)_3_C_1_N][MeSO_4_]	12 ± 6	—
**5** [C_4_C_1_im][MeSO_4_][NTf_2_]	—	61 ± 8
**6** [C_4_C_1_pyrr][NTf_2_][Me_2_PO_4_]	—	41 ± 8
**7** [C_4_C_1_im][NTf_2_][Me_2_PO_4_]	—	41 ± 8
**8** [C_4_C_1_im][Me-DBU][MeSO_4_]	24 ± 11	—
**9** [C_4_C_1_im][C_1_C_1_C_1_pz][OTf]	24 ± 7	—
**10** [C_4_C_1_im][Me-DBU][MeSO_4_][NTf_2_]	24 ± 11	61 ± 8
**11** [C_4_C_1_im][C_4_C_1_pyrr][OTf][NTf_2_]	13 ± 7	59 ± 9

**Table 3:** Differences in the hydrogen-bond acidity, Δ*α*, and the hydrogen-bond basicity, Δ*β*, for constituent cations and anions of ionic liquid mixture series **1–11**. Values were determined either experimentally (‘exp’), or from the arithmetic mean of a series of computational literature values (‘ave’)^58,61–63^

Ionic liquid mixture series	Δ*α*_ave_	Δ*α*_ave_	Δ*β*_exp_	Δ*β*_exp_
**1** [C_4_C_1_im]Cl[OTf]	—	—	0.40	0.40
**2** [C_4_C_1_im][MeSO_4_][Me_2_PO_4_]	—	—	0.36	0.50
**3** [C_4_C_1_im][OTf][NTf_2_]	—	—	0.21	0.22
**4** [C_4_C_1_im][(HOC_2_)_3_C_1_N][MeSO_4_]	0.24	0.47	—	—
**5** [C_4_C_1_im][MeSO_4_][NTf_2_]	—	—	0.40	0.40
**6** [C_4_C_1_pyrr][NTf_2_][Me_2_PO_4_]	—	—	0.76	0.90
**7** [C_4_C_1_im][NTf_2_][Me_2_PO_4_]	—	—	0.76	0.90
**8** [C_4_C_1_im][Me-DBU][MeSO_4_]	0.37	0.39	—	—
**9** [C_4_C_1_im][C_1_C_1_C_1_pz][OTf]	0.10	0.10	—	—
**10** [C_4_C_1_im][Me-DBU][MeSO_4_][NTf_2_]	0.37	0.39	0.40	0.40
**11** [C_4_C_1_im][C_4_C_1_pyrr][OTf][NTf_2_]	0.16	0.18	0.21	0.22

**Table 4:** Differences in the dipole moments of the constituent cations, Δ*p* ^cation^, and anions, Δ*p* ^anion^, of the ionic liquid mixture series **1–11** (units debye, D). Values in parentheses refer to Δ*p* values when a less predominant or higher energy ion conformation is employed

Ionic liquid mixture series	Δ*p* ^cation^	Δ*p* ^anion^
**1** [C_4_C_1_im]Cl[OTf]	—	4.11
**2** [C_4_C_1_im][MeSO_4_][Me_2_PO_4_]	—	1.36 (0.89) (1.16)
**3** [C_4_C_1_im][OTf][NTf_2_]	—	3.91 (0.21)
**4** [C_4_C_1_im][(HOC_2_)_3_C_1_N][MeSO_4_]	4.21 (1.07)	—
**5** [C_4_C_1_im][MeSO_4_][NTf_2_]	—	0.25 (3.87)
**6** [C_4_C_1_pyrr][NTf_2_][Me_2_PO_4_]	—	1.11 (5.23) (0.64)
**7** [C_4_C_1_im][NTf_2_][Me_2_PO_4_]	—	1.11 (5.23) (0.64)
**8** [C_4_C_1_im][Me-DBU][MeSO_4_]	3.64	—
**9** [C_4_C_1_im][C_1_C_1_C_1_pz][OTf]	3.26	—
**10** [C_4_C_1_im][Me-DBU][MeSO_4_][NTf_2_]	3.64	0.25 (3.87)
**11** [C_4_C_1_im][C_4_C_1_pyrr][OTf][NTf_2_]	2.61	3.91 (0.21)

**Table 5:** Differences in the ‘charge arm’ of constituent cations, Δ*R*cationca, and anions, Δ*R*anionca, for mixture series **1–11** (units angstroms, Å), calculated from NBO charges (units e). Values in parentheses refer to Δ*R*_ca_ values when a less predominant or higher energy ion conformation is employed

Ionic liquid mixture series	Δ*R*cationca	Δ*R*anionca
**1** [C_4_C_1_im]Cl[OTf]	—	0.52
**2** [C_4_C_1_im][MeSO_4_][Me_2_PO_4_]	—	0.38 (0.55)
**3** [C_4_C_1_im][OTf][NTf_2_]	—	0.49 (0.29)
**4** [C_4_C_1_im][(HOC_2_)_3_C_1_N][MeSO_4_]	0.70 (0.36)	—
**5** [C_4_C_1_im][MeSO_4_][NTf_2_]	—	0.23 (1.01)
**6** [C_4_C_1_pyrr][NTf_2_][Me_2_PO_4_]	—	0.61 (1.39) (0.32)
**7** [C_4_C_1_im][NTf_2_][Me_2_PO_4_]	—	0.61 (1.39) (0.32)
**8** [C_4_C_1_im][Me-DBU][MeSO_4_]	0.76	—
**9** [C_4_C_1_im][C_1_C_1_C_1_pz][OTf]	0.69	—
**10** [C_4_C_1_im][Me-DBU][MeSO_4_][NTf_2_]	0.76	0.23 (1.01)
**11** [C_4_C_1_im][C_4_C_1_pyrr][OTf][NTf_2_]	0.57	0.49 (0.29)

One possible explanation for the deviations from ideal behaviour is that they are due to differences in the sizes of ions. Navia *et al.* suggested, on the basis of ionic liquid mixture series [C_4_C_1_im][BF_4_][PF_6_] and [C_4_C_1_im][BF_4_][MeSO_4_], that differences in the sizes of the ions of the component ionic liquids could lead to differences in the availability of free space, which in turn could lead to the negative Δ*V*_m_ values upon mixing.^12^ To explore this possibility, the differences in the molar volumes, Δ*V*_m_, of the constituent cations and anions were obtained from DFT volume calculations for all the mixture series **1–11** (Table 2). Where multiple stable conformations of an ion occur, *V*_m_ for the different conformations were found to be similar (±2 cm^3^ mol^–1^), and the lowest in energy or demonstrably most prevailing conformer (see later discussion of [NTf_2_]^–^) was used to construct the data in Table 2. Single ion *V*_m_ are reported in the ESI (Table E7†).

The Δ*V*_m_ values indicate no clear correlation between the size difference of the ions and the extent of deviation from expected behaviour. Typically, the size differences between the anions were of greater magnitude than size differences between the investigated cations. The largest molar volume differences were encountered for mixtures of [NTf_2_]^–^ with either [OTf]^–^ or [MeSO_4_]^–^; mixture series **3**, **5**, **10** and **11** (Δ*V*anionm = 59–61 cm^3^ mol^–1^). Mixtures of **5** and **10** exhibited positive deviation with respect to molar volume (Fig 3b and E3†), yet in contrast series **3** and **11** displayed almost perfect linear behaviour. Furthermore, the two mixture series that exhibited consistent deviation from the anticipated behaviour, **6** and **7**, incorporating the [NTf_2_]^–^ and [Me_2_PO_4_]^–^ anions, have comparatively small values of Δ*V*anionm, 41 ± 8 cm^3^ mol^–1^. This value for **6** and **7** was similar to that of mixtures of [C_4_C_1_im]Cl[OTf], **1**, a mixture series which gave close to ideal behaviour across the spectrum of investigated physical properties. Therefore, explanation for non-ideality on the basis of size differences between the ions seems implausible.

An alternative explanation may lie in the difference in *polarity* or *hydrogen bond* character of the ionic liquid ions that are incorporated into the mixture.

The multi-parameter Kamlet–Taft empirical polarity scale has been employed extensively for the characterization of ionic liquids.^55^ Of particular interest to the ionic liquid mixtures discussed in this investigation are ‘hydrogen bond acidity’, *α*,^56^ dictated largely by the cation, and the ‘hydrogen bond basicity’, *β*,^57^ which depends heavily on the nature of the anion.

Previously, we have demonstrated that the values of *α* and *β* can be predicted effectively by using a variety of computational descriptors.^58^ Experimental (from the neat component) and predicted values of *α* and *β* for the individual ions are tabulated for all cations and anions employed in this investigation, denoted ‘exp’ for experimental values and ‘ave’ for the average of all computational descriptors. Employing these previously determined values, differences in hydrogen bond acidity and basicity, Δ*α* and Δ*β*, for all constituents in the ionic liquid mixture series **1–11** are listed in Table 3.

Strikingly, the largest values of Δ*β* correspond to the ionic liquid mixture series **6** and **7** (Δ*β*_exp_ = 0.90, Δ*β*_ave_ = 0.76), the two series that gave consistently non-ideal behaviour in the molar volume, glass transition, viscosity and conductivity experiments. The large values of Δ*β* arise from the atypically high hydrogen bond basicity of the [Me_2_PO_4_]^–^ anion and the very low hydrogen bond basicity of the [NTf_2_]^–^ anion.

The binary mixture series [C_4_C_1_im][MeSO_4_][NTf_2_], **5**, and the reciprocal binary ionic liquid mixture series [C_4_C_1_im][Me-DBU][MeSO_4_][NTf_2_], **10**, also exhibited a smooth, parabolic excess molar volume curve (indicative of non-ideality). Values of Δ*α* and Δ*β* for these mixture series are also moderately high (∼0.4). However, these values are comparable to other mixture series which did exhibit ideal mixing behavior, *e.g.*, **1** and **2**.

Thus, it appears that substantial differences in hydrogen bonding behaviours might contribute to the observed deviations from ideality for the investigated ionic liquid mixtures. For example, incorporating the poor hydrogen bond acceptor [NTf_2_]^–^ (*β*_ave_ = 0.25) anion into a [Me_2_PO_4_]^–^ (*β*_ave_ = 1.01) containing liquid would place the [NTf_2_]^–^ anion into a cavity formerly occupied by a [Me_2_PO_4_]^–^ anion. The inability of the [NTf_2_]^–^ anion to maintain the network of hydrogen bonds could lead to a change in the structure and ordering of the liquid in which there is a net reduction in favourable interactions and an increase in molar volume. However, this could not be the only explanation and other factors do appear to be critical as well.

The anisotropy of ionic liquid ions and the directional nature of bonding interactions contribute to the structural and dynamic properties of the bulk liquid.^59,60^ The dipole moment, *p*, of a chemical species is a measure of its polarity and anisotropy, given as the magnitude of two equal, but opposite, charges multiplied by the distance between these (in units of debye, D).

Strictly, the term ‘dipole moment’ can refer only to species with no overall charge; ions therefore cannot have a *dipole* moment, instead possessing a *monopole* moment.^64^ However, a value of ‘dipole moment’ evolves from DFT calculations. This value is the distance between the centre of mass of the ion and its centre of charge.

**Fig. 9:**
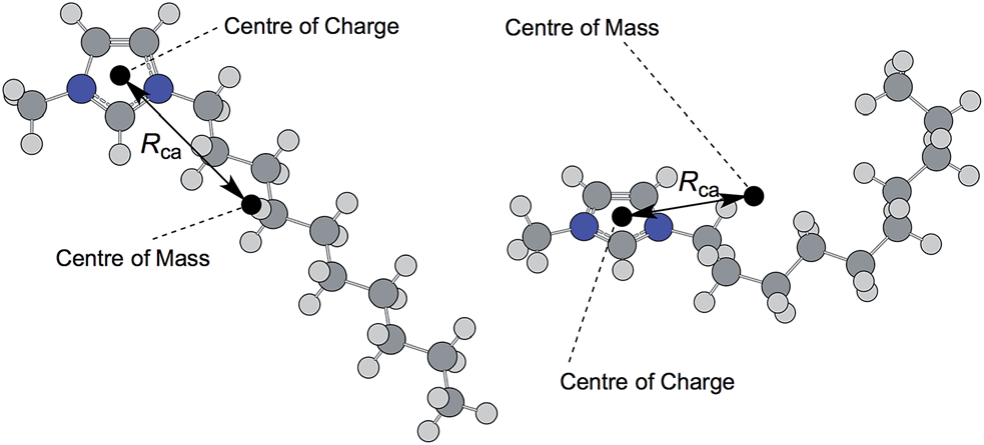
Schematic diagram representing the dramatic influence of ion geometry on the ‘charge arm’, *R*_ca_, using the example of the 1-decyl-3-methylimidazolium cation, [C_10_C_1_im]^+^, in both an ‘extended’ all-*anti* conformer (left), *R*_ca_ = 4.49 Å, and ‘coiled’, part-*gauche* conformer (right), *R*_ca_ = 3.64 Å.

Kobrak and co-workers developed the concept of the ‘charge arm’, as a description of the anisotropy of ionic liquid ions, and related these values to the viscosity of ionic liquids.^65,66^ The charge arm, *R*_ca_, is defined as the distance between the center of mass and the center of charge (Fig. 9), using the ‘Natural Bond Orbital’ (NBO) model.^67^ The charge arm is able to describe multipoles in ions (with overall charge), unlike the true dipole moment, *p*, which describes neutral species. For simplicity, and to differentiate from the ‘charge arm’ parameter which is also a measure of distance between the centres of charge and mass of the ions, we will refer to DFT-calculated *p* values as the ‘dipole moment’. In practice, our calculated ‘dipole moments’ differ from the ‘charge arm’ because of the model by which atomic charges are calculated; the DFT ‘dipole moments’ are determined using the CHelpG model,^68^ our ‘charge arm’ calculations employ the NBO model,^67,69^ a method acknowledged for being relatively insensitive to the basis set.^70^ Hence, performing ‘charge arm’ calculations with the ChelpG model instead of the NBO model reproduces values in close agreement with the DFT ‘dipole moment’ values.

Differences in the dipole moment for the cations and anions (Δ*p* ^cation^ and Δ*p* ^anion^, respectively) were calculated for all ionic liquid mixture series, **1–11** (Table 4). The principle values of Δ*p* are listed first, followed by values in parentheses referring to when an ion conformer of higher energy or demonstrably less prevalent in solution is employed. Particular ions, notably [Me_2_PO_4_]^–^, [NTf_2_]^–^ and [(HOC_2_)_3_C_1_N]^+^, show a significant dipole moment dependence on ion conformation.

The most significant Δ*p* values correspond to series **1** and **4**; in these instances, large differences in the *cation* dipole moment, Δ*p* ^cation^, provides the explanation (although comprehensive conformational analysis of the alkyl chain-containing [C_4_C_1_im]^+^ cation would be necessary, to fully confirm this). These mixture series adhere closely to ideal law, with little deviation. Thus, differences in ion dipole moments do not appear to provide a convincing nale for deviation from ideality when it does occur.

Previously, we evaluated charge arm lengths, *R*_ca_, for the [C_4_C_1_im]^+^ cation, and a novel siloxane-incorporating imidazolium cation, [(SiOSi)C_1_C_1_im]^+^.^71^ Using the same methodology, the charge arm lengths were calculated for all cations and anions in this study, using the ‘Natural Bond Orbital’ (NBO) charge model, and are tabulated in the ESI (Table E7†). Subsequently, charge arm differences (Δ*R*cationca and Δ*R*anionca) were determined for all mixture series **1–11**, and are listed in Table 5. Given the high degree of flexibility and large number of conformations possible for several of the ions, the principle Δ*R*_ca_ values in Table 5 were determined using the lowest energy (most prevalent) conformer of each ion, as for the Δ*p* values above (Table 4). The Δ*R*_ca_ values in parentheses refer to calculations based on a less prevalent or higher energy conformer for one or several ions.

The results indicate a possible relationship between the *magnitude* of Δ*R*_ca_ values and the extent of deviation from ideality for ionic liquid mixture series (Table 5). Mixtures of **6** and **7**, which consistently deviated from ideal mixing law, exhibited the largest primary Δ*R*anionca value for mixtures containing different anions (0.61 Å), caused by the long charge arm of low-energy [Me_2_PO_4_]^–^ (1.42 Å) and the comparatively short charge arm of *cis* [NTf_2_]^–^ (0.81 Å). Larger values of Δ*R*_ca_ were exhibited by mixture series **4**, **8**, **9** and **10**, which contain different cations series. However, the mixture series **5** and **10**, which gave the highest excess molar volumes, exhibited low primary Δ*R*anionca values of 0.23 Å. Therefore, on initial inspection, the charge arm differences, Δ*R*_ca_, do not account well for the deviations from ideal behaviour.

However, *R*_ca_ values vary dramatically upon changing the ion *conformation*. This is in contrast to calculated molar volumes, *V*_m_, which were almost identical for differing conformations of the same ion. One individual conformer of [(HOC_2_)_3_C_1_N]^+^ exhibited a very high *R*_ca_ value of 1.56 Å, in contrast to another conformer with a charge arm length of just 0.50 Å. The impact of the ion geometry on the charge arm, *R*_ca_, is highlighted in Fig. 9, with reference to the 1-decyl-3-methylimidazolium cation, [C_10_C_1_im]^+^.

The disparity in *R*_ca_ between different conformers was exemplified by [NTf_2_]^–^; the *cis* conformer (C1 symmetry) has a charge arm of 0.81 Å, whereas the *trans* conformer (C2 symmetry) has a charge arm length of just 0.03 Å suggesting that the centers of mass and of charge almost coincide. Critically, the uncorrected energy difference between these two conformers (<3.5 kJ mol^–1^) is smaller than the error in the calculation, consistent with previous investigations.^72,73^ Hence, the *cis* and *trans* conformers can be regarded as degenerate in energy and therefore both potentially present in the mixtures.

Previous literature studies have highlighted examples of both the *cis* conformer predominating, in crystalline [C_1_C_1_im][NTf_2_],^74^ and the *trans* conformer in crystalline [C_2_C_2_C_2_^2^im][NTf_2_]^74^ and liquid-phase [C_1_C_1_im][NTf_2_].^75^ Furthermore, Raman spectroscopy has indicated that for the ionic liquids [C_2_C_1_im][NTf_2_] and [C_4_(C_1_)_3_N][NTf_2_], the cooling rate has a profound impact on the dominant conformation of the [NTf_2_]^–^ anion.^72,76^

In order to assess prevailing geometries of the [NTf_2_]^–^ anion in mixture series **1–11**, [C_4_C_1_im][NTf_2_] was studied using Raman spectroscopy, alongside 1 : 1 mixtures [C_4_C_1_im][A]_0.5_[NTf_2_]_0.5_, where A = [OTf]^–^ (**3**), [MeSO_4_]^–^ (**5**) and [Me_2_PO_4_]^–^ (**7**). Each mixture was carefully dried prior to Raman experiments, to <500 ppm H_2_O, as determined from Karl Fischer titn. Subsequently, Raman frequencies were calculating using DFT. Similar to the method employed by Faria *et al.*,^76^ our DFT-calculated wavenumbers were scaled by a factor of 1.047, based on the disparity between the intense peak at ∼736 cm^–1^ in the experimental spectra and the calculated value, 702.9 cm^–1^, for the same peak. Full experimental and computational procedures are detailed in the ESI.† Furthermore, experimental and calculated Raman spectra are displayed in the ESI (Fig. E10†).

The experimental Raman spectra, for each of the four above-described liquids, exhibited fairly broad peaks that were non-trivial to assign. To differentiate between the predominant *trans* or *cis* geometries of the [NTf_2_]^–^ anion, specific regions of the Raman spectra were focused upon.

Initially, the region of 220–480 cm^–1^ was investigated (Fig. E10b†). The simple ionic liquid [C_4_C_1_im][NTf_2_], and its mixtures with polar anions, [MeSO_4_]^–^ and [Me_2_PO_4_]^–^, displayed intense, broad peaks centred at approximately 277 and 322 cm^–1^. These are in close agreement with our calculated values, 277 and 321 cm^–1^, for the *cis* geometry of [NTf_2_]^–^. The DFT calculations predicted no equivalent peaks at these locations for the *trans* isomer.

By contrast, the mixture incorporating the low charge arm trifluoromethanesulfonate anion, [C_4_C_1_im][OTf]_0.5_[NTf_2_]_0.5_, exhibited a substantial shift in position of the latter peak, to ∼313 cm^–1^, close to the value observed by Faria and co-workers for the *trans* [NTf_2_]^–^ conformer in cold-crystallised [C_4_(C_1_)_3_N][NTf_2_] (315 cm^–1^),^76^ and in reasonable agreement with our scaled calculated Raman band at 306 cm^–1^ for *trans* [NTf_2_]^–^ (Fig. E10h†).

The Raman spectra from 595–675 cm^–1^ were also examined (Fig. E10c–f†). With reference to mixtures of salt Li[NTf_2_] with diglyme, it has been demonstrated that peaks in this low-intensity region may be used in order to distinguish between *trans* (629 cm^–1^) and *cis* (653 cm^–1^) ion conformers.^77,78^ Each of the Raman spectra in this investigation exhibited both of these two peaks indicating some appreciable contribution from each of the *trans* and *cis* ion conformers of [NTf_2_]^–^.

Therefore, the obtained Raman spectra, and previous investigations, highlight a contribution from both the *cis* and *trans* geometries of [NTf_2_]^–^. However, on the basis of the above-described experiments, it is sensible to obtain primary Δ*R*_ca_ and Δ*p* values for mixture series **3** and **11** using the calculated *R*_ca_ and Δ*p* values of *trans* [NTf_2_]^–^, instead using *cis* [NTf_2_]^–^ for series **5**, **6**, **7** and **10** (Tables 4 and 5). Nevertheless, we acknowledge that these values represent only a rudimentary approximation.

We further considered the potential influence of ion geometry on the charge arm. *R*_ca_, and the SCF energy, Δ*E* (kJ mol^–1^), with detailed investigation into the [NTf_2_]^–^, [Me_2_PO_4_]^–^ and [MeSO_4_]^–^ anions; conformational analysis was performed about an *S*–*N*–*S*–*C*F_3_, *O*–*P*–*O*–*C*H_3_ or *O*–*S*–*O*–*C*H_3_ dihedral angle, respectively (shown in graphical form in Fig. E11†). These anions were selected because their mixtures gave the most significant (but still small) deviations from ideal behaviour in the measured physical properties. The other two anions in this investigation, Cl^–^ and [OTf]^–^, have little or no conformational flexibility, and therefore the charge arm value is relatively fixed (or zero for chloride, by definition).

The results reveal significant differences between the charge arm ‘flexibility’ of the anions. The charge arm of [NTf_2_]^–^ changes substantially with the ion conformation, with an *R*_ca_ range of >1.1 Å and the energy barrier for rotation is fairly low (<25 kJ mol^–1^). Therefore, [NTf_2_]^–^ may be regarded as a relatively *flexible* ion, able to adapt its charge arm length with ease. Similarly, the [Me_2_PO_4_]^–^ anion has a difference of ∼1 Å between its largest and smallest charge arm values (although the dihedral scan of one methoxy group, with free rotation of the other, does not reveal all the stable ion geometries). By contrast, the *R*_ca_ range of [MeSO_4_]^–^ is smaller (<0.1 Å); therefore, this ion may be considered *inflexible*.

One might expect that ions with flexible charge arms would reorient themselves to minimize the difference in charge arm of the ions in the mixture. To an extent, this appears to be the case, based upon the Raman spectra for [C_4_C_1_im][NTf_2_], and its equimolar mixtures with the [OTf]^–^, [MeSO_4_]^–^ and [Me_2_PO_4_]^–^ anions; when the [NTf_2_]^–^ anion is accompanied by fairly high charge arm ions (*R*_ca_ > 1, [MeSO_4_]^–^, [Me_2_PO_4_]^–^ or simply [C_4_C_1_im]^+^), it adopts a predominant *cis* (high *R*_ca_) geometry. By contrast, the incorpon of low charge arm [OTf]^–^ seems to change the prevailing composition of [NTf_2_]^–^ towards low charge arm *trans* geometry. However, when the highly ‘flexible’ [NTf_2_]^–^ anion is paired with the fairly flexible [Me_2_PO_4_]^–^ anion, the deviation from ideal mixing law is similar, or greater, than when incorporated with the highly inflexible [MeSO_4_]^–^ anion, depending on the investigated physical property. Therefore, it is apparent that ion flexibility does not play a substantial role in minimising deviation from ideal mixing.

We did not measure ionic liquid mixtures containing cations of different alkyl chain lengths *via* experimental methods. However, we can apply the charge arm analysis to a series of [C_*n*_C_1_im]^+^ (*n* = 1–10) cations, previously investigated by Canongia Lopes *et al.*,^11^ and furthermore for cation series [C_*n*_(C_1_)_3_N]^+^ (*n* = 1–14), and the anion series [C_*n*_CO_2_]^–^ and [C_*n*_SO_4_]^–^ (*n* = 1–14). For simplicity, all cations and anions were optimised in the ‘all *trans*/*anti*’ conformation, with maximal extension of the alkyl chain. *R*_ca_ values for the cations are tabulated and shown in graphical form in the ESI (Table E8 and Fig. E12†).

Unsurprisingly, the results demonstrate a sequential increase in *R*_ca_ as the alkyl chain ‘*n*’ is extended from one carbon (*R*_ca_ = 0.16 Å for [C_1_C_1_im]^+^) to 10 (*R*_ca_ = 4.49 Å for [C_10_C_1_im]^+^). For the example of dialkylimidazolium cations, additional CH_2_ units in the homologous series shift the centre of mass away from the imidazolium ring, whilst the positive charge remains primarily on the ring.

Applying these charge arm calculations retroactively to these prior results, they are consistent with the observation that [C_2_C_1_im]_0.5_[C_10_C_1_im]_0.5_[NTf_2_] exhibits a greater excess molar volume than ionic liquid mixture [C_4_C_1_im]_0.47_[C_8_C_1_im]_0.53_[NTf_2_].^11^ Therefore, large values of Δ*R*_ca_ for [C_*n*_C_1_im]^+^ ions may be a contributing factor to deviation from ideality in this circumstance, although conformation of the ions is an important considen.

The substitution of long alkyl chains onto the cations and anions introduces a further complication, namely, the potential for the formation of hydrophobic/hydrophilic regions. The presence of such ordered ‘domains’ is likely to represent an additional source of non-ideality for ionic liquid mixtures. Although we have herein chosen to omit ions with large aliphatic groups, a study into amphiphilic ions is nevertheless worthy of further study and is an area of our ongoing research.

Of the investigated experimental and computational parameters, it is clear that no single property is able to account for, or predict, the extent of deviation from ideality for ionic liquid mixtures on its own. Our results indicate that differences in hydrogen-bond acidity/basicity of ions, and/or in the length and flexibility of the charge arm (or dipole moment) each contribute partially to the mixing behaviours of the ionic liquids studied. It is simple to imagine that inserting, for example, an anion of high charge arm into a site in the structure of an ionic liquid composed of anions of low charge arm will be more disrupting to that structure than replacing the low charge arm ion with another of similarly low charge arm. Equally, mixing a low hydrogen-bond basicity ion into the cavity formerly occupied by a high basicity ion may result in the net number of favourable interactions being diminished.

In all the discussion of the factors contributing to non-ideality, it must be recognised and remembered that the absolute deviations are extremely small. Practically, then, stoichiometric mixing of well-defined simple ionic liquids provides mixtures with predictable properties. Therefore, the fact that deviation is very minimal, and also that the number and variety of contributing factors to non-ideality is large, contributes to the difficulty in their elucidation.

## Conclusions

For each physical property investigated, both here and in other literature,^11–15,17–20^ the majority of ionic liquid mixture series were found to behave in a close-to-ideal manner, arising *via* a combination of the ionic liquid ions being similar in many respects and the high degree of charge screening found in ionic liquids.^51–54^ Deviations from ideal mixing behaviors are small (values for the studied mixtures were always confined within the boundaries defined by the pure ionic liquids), and arise from differences between the constituent ions, but are not driven by one individual controlling property. It is clear that what little deviation is seen is related to *both* the differences in short-range interactions, *e.g.* hydrogen bonding, and steric factors, particularly differences in the asymmetry of ions. In our continuing studies, we will be seeking to further elucidate these factors.

Considering the whole body of information available on ionic liquid mixtures, it is striking to what extent the effects of mixing can be correlated with ideal mixing laws, in the absence of chemical reactions leading to new constituent ions.^79–82^ For the academic or industrial chemist, understanding that formulations of ionic liquids consistently adhere closely to ideal mixing laws allows for a desired physical property to be obtained by careful stoichiometric mixing of two or more well-defined simple ionic liquids. Substantial economic and scientific benefits are implied by these physical insights, and this additional handle for fine-tuning of ionic liquids further strengthens their reputation as ‘designer solvents’.
