# Supplementary material for: A physicochemical investigation of ionic liquid mixtures
Source: Chem Sci. 2014 Nov 5;6(2):1101–14. doi: 10.1039/c4sc02931c (PMC5811077; doi:10.1039/c4sc02931c)
Supplement: Supplementary file 1 [file SC-006-C4SC02931C-s001.pdf]

Electronic Supplementary Material (ESI) for Chemical Science.
This journal is © The Royal Society of Chemistry 2014

Chemical Science

A physicochemical investigation of ionic liquid mixtures

Matthew T. Clough,[a] Colin R. Crick,[a] John Gräsvik,[a] Patricia A. Hunt,*[a] Heiko Niedermeyer,[a] Tom Welton*[a] and Oliver P. Whitaker[b]

[a] *Department of Chemistry, Imperial College London, London, SW7 2AZ, UK*
[b] *Tonbridge School, Tonbridge, Kent, TN9 1JP, UK*

* *Corresponding Authors: p.hunt@imperial.ac.uk*
t.welton@imperial.ac.uk

Electronic Supplementary Information

1. **Laboratory procedures** 2
2. **Synthesis of ionic liquids** 3
3. **Molar volumes of ionic liquid mixtures** 7
4. **Phase behaviour / glass transition temperatures of ionic liquid mixtures** 10
5. **Viscosities of ionic liquid mixtures** 13
6. **Conductivities of ionic liquid mixtures** 14
7. **Walden relationship of viscosity and conductivity for ionic liquid mixtures** 15
8. **Thermal stabilities of ionic liquid mixtures** 18
9. **Computational procedures** 20
10. **Molar volume calculations, charge arm calculations and Raman spectroscopy** 20

1. Laboratory procedures

General Procedures

Prior to measurements, all samples were dried under vacuum until a water content of < 150 ppm (w/w) was established *via* Karl-Fischer titration.

Density Measurements

Density measurements were performed in an Anton Paar 'DMA38' vibrating tube density meter at 25 °C. The error of the instrument given by the manufacturer was ± 0.001 g cm^{-3}, although the reproducibility was one order of magnitude better.

Differential Scanning Calorimetry (DSC) Measurements

Differential Scanning Calorimetry (DSC) results were obtained using aluminium sample pans of diameter 7 mm. Between 7 - 9 mg of the ionic liquid was measured into the aluminium sample pan. A small incision was made in the tops of both the sample and reference pans. A drying procedure was then implemented: the sample pan was heated to 100 °C for 20 minutes in the calorimeter, to remove water. The sample pan was then quickly re-weighed, in order to determine the dry weight of the ionic liquid. The sample was measured between -100 and +100 °C, with a heating / cooling rate of 20 °C min^{-1}.

Viscosity Measurements

Viscosity measurements were performed on a TA instruments 'AR2000ex' rheometer fitted with a peltier plate at 25 °C, using a 40 mm, 2° steel cone. Measurements were performed at an angular velocity between 0.1 and 10 rad s^{-1} under a nitrogen atmosphere. An error of 5% was established from the manufacturer's information and measurements on standard liquids, though reproducibility was usually better.

Conductivity Measurements

The conductivity measurements were performed using a home-built conductivity probe with two platinum paddles of size approximately 5 mm × 5 mm. The temperature was kept at 25 °C using a water bath in a jacketed beaker attached to a recirculator and the samples were allowed to reach thermal equilibrium for 30 minutes. The measurements were performed under argon atmosphere on a CH Instruments CHI760C in a frequency range from 1 Hz to 1 x 10^5 Hz at an amplitude of 1 x 10^{-4} V. Prior to each set of measurement the probe was calibrated using three commercially available standards (HANNA instruments) with known conductivities of 84 μS cm^{-1}, 1413 μS cm^{-1} and 12880 μS cm^{-1}. The average error was found to be 2%.

Thermogravimetric Analysis (TGA) Measurements

Temperature-ramped Thermogravimetric Analysis (TGA) experiments were performed on a PerkinElmer 'Pyris 1 TGA' thermogravimetric analyzer, using platinum sample pans of 6 mm diameter. TGA experiments were carried out in the range of 120 - 600 °C. Between 3 - 36 mg of the ionic liquid was measured into the platinum pan. A ramping rate of 10 °C min^{-1} and a nitrogen flow of 20 ml min^{-1} were used for temperature-ramped experiments. A drying procedure was employed, whereby the ionic liquid sample was maintained at 120 °C for a period of up to six hours, as appropriate. This drying procedure was justified since the onset decomposition temperature of each compound is higher by at least 150 °C than the 120 °C drying temperature, and the drying period is short. Therefore, actual decomposition of each ionic liquid during the drying period will be negligible.

Raman Spectroscopy Measurements

Raman measurements were performed using a custom built Raman microscope described previously,[1,2] using a laser power of 8.1 mW focused with a 40x objective (Olympus UAPO40X3/340, 40x, 0.6 NA). The exposure time for each spectrum was 2 seconds.

2. Synthesis of ionic liquids

All reagents used were obtained from VWR or Fisher Scientific at highest purity, and were dried using suitable drying agents. The cations and anions incorporated into the investigated ionic liquid mixtures are displayed below (Fig. E1). The synthesis of ionic liquids is described below.

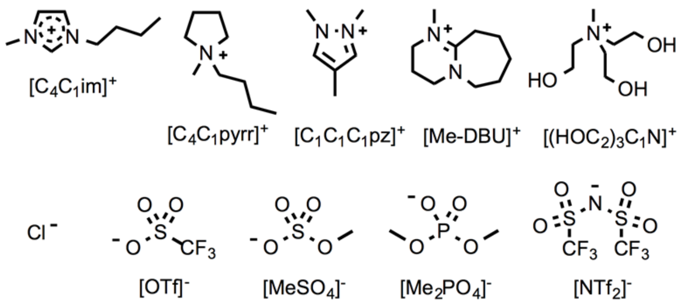

Fig. E1: Ionic liquids cations and anions incorporated into this investigation; 1-butyl-3-methylimidazolium ($[C_4C_1im]^+$), 1-methyl-1-butylpyrrolidinium ($[C_4C_1pyrr]^+$), 1,2,4-trimethylpyrazolium ($[C_1C_1C_1pz]^+$), 1-methyl-1,8-diazabicyclo[5.4.0]undec-7-enium ($[Me\text{-}DBU]^+$) and triethanolmethylammonium ($[(HOC_2)_3C_1N]^+$) cations, chloride (Cl^-), trifluormethanesulfonate ($[OTf]^-$), methyl sulfate ($[MeSO_4]^-$), dimethyl phosphate ($[Me_2PO_4]^-$) and *bis*(trifluoromethanesulfonyl)imide ($[NTf_2]^-$) anions.

Table E1: Ionic liquid binary and reciprocal binary mixtures investigated in this contribution, **1**-**11**. For each mixture, the molar quantities, χ, of the two constituent ionic liquids were varied.

Ionic Liquid Mixture		Constituent ILs
**1**	$[C_4C_1im]Cl[OTf]$	$[C_4C_1im]Cl$<br>$[C_4C_1im][OTf]$
**2**	$[C_4C_1im][MeSO_4][Me_2PO_4]$	$[C_4C_1im][MeSO_4]$<br>$[C_4C_1im][Me_2PO_4]$
**3**	$[C_4C_1im][OTf][NTf_2]$	$[C_4C_1im][OTf]$<br>$[C_4C_1im][NTf_2]$
**4**	$[C_4C_1im][(HOC_2)_3C_1N][MeSO_4]$	$[C_4C_1im][MeSO_4]$<br>$[(HOC_2)_3C_1N][MeSO_4]$
**5**	$[C_4C_1im][MeSO_4][NTf_2]$	$[C_4C_1im][MeSO_4]$<br>$[C_4C_1im][NTf_2]$
**6**	$[C_4C_1pyrr][NTf_2][Me_2PO_4]$	$[C_4C_1pyrr][NTf_2]$<br>$[C_4C_1pyrr][Me_2PO_4]$
**7**	$[C_4C_1im][NTf_2][Me_2PO_4]$	$[C_4C_1im][NTf_2]$<br>$[C_4C_1im][Me_2PO_4]$
**8**	$[C_4C_1im][Me\text{-}DBU][MeSO_4]$	$[C_4C_1im][MeSO_4]$<br>$[Me\text{-}DBU][MeSO_4]$
**9**	$[C_4C_1im][C_1C_1C_1pz][OTf]$	$[C_4C_1im][OTf]$<br>$[C_1C_1C_1pz][OTf]$
**10**	$[C_4C_1im][Me\text{-}DBU][MeSO_4][NTf_2]$	$[C_4C_1im][NTf_2]$<br>$[Me\text{-}DBU][MeSO_4]$
**11**	$[C_4C_1im][C_4C_1pyrr][OTf][NTf_2]$	$[C_4C_1im][OTf]$<br>$[C_4C_1pyrr][NTf_2]$

1-Butyl-3-methylimidazolium chloride. A solution of 1-methylimidazole (210.3 ml, 2.65 mol) in EtOAc (180 ml) was cooled in an ice bath, and 1-chlorobutane (375.0 ml, 3588 mmol) was added dropwise. The resulting liquid was stirred at 45 °C for 14 days. During this time the single-phase solution separated in two layers. After two days at -20 °C white crystals had formed, which were washed repeatedly with EtOAc (3 × 180 ml) and were subsequently dried under vacuum, to yield 1-butyl-3-methylimidazolium chloride (224.47 g, 48.5%). δ_H (ppm) (400 MHz, $CDCl_3$) 10.58 (s, 1H, NC*H*N), 7.60 (s, 1H, NCHC*H*N), 7.43 (s, 1H, NC*H*CHN), 4.23 (t, J = 7 Hz, 2H, $NC\mathit{H}_2CH_2$), 4.03 (s, 3H, $NC\mathit{H}_3$), 1.90 - 1.73 (m, 2H, $CH_2C\mathit{H}_2CH_2$), 1.35 - 1.18 (m, 2H, $CH_2C\mathit{H}_2CH_3$), 0.86 (t, J = 7 Hz, 3H, $CH_2C\mathit{H}_3$). δ_C (ppm) (101 MHz, $CDCl_3$) 137.83 (s, N*C*HN), 123.67 (s, NCH*C*HN), 121.98 (s, N*C*HCHN), 49.69 (s, $N\mathit{C}H_2CH_2$), 36.47 (s, $N\mathit{C}H_3$), 32.11 (s, $CH_2\mathit{C}H_2CH_2$), 19.38 (s, $CH_2\mathit{C}H_2CH_3$), 13.37 (s, $CH_2\mathit{C}H_3$). *m/z* ($LSIMS^+$): 139 ($[C_4C_1im]^+$, 100%), 313 ($\{[C_4C_1im]_2Cl\}^+$, 10%). *m/z* ($LSIMS^-$): 35 ($^{35}Cl^-$, 80%), 209 ($\{[C_4C_1im]Cl_2\}^-$, 65%).

1-Butyl-3-methylimidazolium trifluoromethanesulfonate. A solution of 1-butyl-3-methylimidazolium chloride (145.59 g, 833 mmol) in CH_2Cl_2 (500 ml) was added to lithium trifluoromethanesulfonate (134.12 g, 860 mmol), and the resulting slurry was stirred for 60 hours at room temperature. The white precipitate was filtered off, and the remaining liquid was diluted with CH_2Cl_2 to a total volume of ~ 850 ml. The diluted solution was subsequently washed with water (34 × 3 ml) until halide free by $AgNO_3$ test. The solvent was evaporated under vacuum, to yield 1-butyl-3-methylimidazolium trifluoromethanesulfonate as a colourless, free-flowing liquid (193.18 g, 80.4%). δ_H (ppm) (400 MHz, $CDCl_3$) 8.77 (s, 1H, NC*H*N), 7.30 - 7.24 (m, 2H, NC*H*C*H*N), 3.98 (t, J = 7 Hz, 2H, $NC\mathit{H}_2CH_2$), 3.74 (s, 3H, $NC\mathit{H}_3$), 1.70 - 1.56 (m, 2H, $CH_2C\mathit{H}_2CH_2$), 1.19 - 1.04 (m, 2H, $CH_2C\mathit{H}_2CH_3$), 0.70 (t, J = 6 Hz, 3H, $CH_2C\mathit{H}_3$). δ_C (ppm) (101 MHz, $CDCl_3$) 135.94 (s, N*C*HN), 123.47 (s, NCH*C*HN), 122.21 (s, N*C*HCHN), 120.38 (q, J = 320 Hz, $\mathit{C}F_3SO_3$), 49.35 (s, $N\mathit{C}H_2CH_2$), 35.82 (s, $N\mathit{C}H_3$), 31.55 (s, $CH_2\mathit{C}H_2CH_2$), 18.91 (s, $CH_2\mathit{C}H_2CH_3$), 12.87 (s, $CH_2\mathit{C}H_3$). *m/z* (ESI^+): 139 ($[C_4C_1im]^+$, 100%), 427 ($\{[C_4C_1im]_2[OTf]\}^+$, 40%). *m/z* (ESI^-): 149 ($[OTf]^-$, 85%), 437 ($\{[C_4C_1im][OTf]_2\}^-$, 100%), 725 ($\{[C_4C_1im]_2[OTf]_3\}^-$, 20%). Elemental analysis (expected): C 37.35% (37.50%), H 5.24% (5.24%), N 9.60% (9.72%). ν/cm^{-1} (neat): 3114 (aromatic C−H stretch, m), 2965 (aliphatic C−H stretch, m), 1575 (aromatic ring def, m), 1467 (aliphatic C−H bend, w), 1256 (asym. S−O stretch, s), 1225 (C−S stretch, s), 1156 (asymm. C−F stretch, s), 1030 (symm. S−O and C−F str., s).

1-Butyl-3-methylimidazolium *bis*(trifluoromethanesulfonyl)imide. A solution of 1-butyl-3-methylimidazolium chloride (67.80 g, 388 mmol) in CH_2Cl_2 (200 ml) was added to lithium *bis*(trifluoromethanesulfonyl)imide (117.00 g, 408 mmol), and the resulting slurry was stirred for 60 hours at room temperature. The white precipitate was filtered off and the remaining liquid was diluted with CH_2Cl_2 to a total volume of ~ 750 ml. The diluted solution was subsequently washed with water (8 × 100 ml) until halide free by $AgNO_3$ test. The solvent was evaporated under vacuum to yield 1-butyl-3-methylimidazolium *bis*(trifluoromethanesulfonyl)imide as a colourless, free-flowing liquid (144.25 g, 89%). δ_H (ppm) (400 MHz, $CDCl_3$) 8.46 (s, 1H, NC*H*N), 7.37 (t, J = 2 Hz, 1H, NCHC*H*N), 7.29 (t, J = 2 Hz, 1H, NC*H*CHN), 4.07 (t, J = 7 Hz, 2H, $NC\mathit{H}_2CH_2$), 3.80 (s, 3H, $NC\mathit{H}_3$), 1.82 - 1.68 (m, 2H, $CH_2C\mathit{H}_2CH_2$), 1.30 - 1.15 (m, 2H, $CH_2C\mathit{H}_2CH_3$), 0.79 (t, J = 7 Hz, 3H, $CH_2C\mathit{H}_3$). δ_C (ppm) (101 MHz, $CDCl_3$) 135.94 (s, N*C*HN), 123.40 (s, NCH*C*HN), 122.15 (s, N*C*HCHN), 119.81 (q, J = 321 Hz, $(\mathit{C}F_3O_2S)_2N$), 49.36 (s, $N\mathit{C}H_2CH_2$), 35.47 (s, $N\mathit{C}H_3$), 31.51 (s, $CH_2\mathit{C}H_2CH_2$), 18.85 (s, $CH_2\mathit{C}H_2CH_3$), 12.36 (s, $CH_2\mathit{C}H_3$). *m/z* (ESI^+): 139 ($[C_4C_1im]^+$, 100%), 558 ($\{[C_4C_1im]_2[NTf_2]\}^+$, 55%). *m/z* ($ESI^-$): 280 ($[NTf_2]^-$, 100%). Elemental analysis (expected): C 28.75% (28.64%), H 3.66% (3.61%), N 9.89% (10.02%). ν/cm^{-1} (neat): 3157 (aromatic C−H stretch, m), 2967 (aliphatic C−H stretch, m), 1569 (aromatic ring def., m), 1466 (aliphatic C−H bend, w), 1348 1330 (asym. S−O stretch, s), 1181 1136 (asym. C−F str., s), 1054 (asym. S−N str., s).

1-Butyl-3-methylimidazolium methyl sulfate. To an ice-cooled solution of 1-butylimidazole (207.18 g, 1.67 mol) in toluene (300 mL), dimethyl sulfate (209.80 g, 1.66 mol) was slowly added dropwise. The resulting mixture was stirred for 72 hours, during which time the solution had separated into two phases. The toluene phase was separated off and the ionic liquid phase was washed with toluene (3 × 300 ml). The remaining solvent was evaporated under vacuum, to yield 1-butyl-3-methylimidazolium methyl sulfate as a light yellow, free-flowing liquid (351.86 g, 84%). The liquid was stirred with activated charcoal for three days and filtered through a pad of neutral alumina to obtain a colorless liquid. δ_H (ppm) (400 MHz, $CDCl_3$) 9.13 (s, 1H, NC*H*N), 7.37 (t, J = 2 Hz, 1H, NCHC*H*N), 7.32 (t, J = 2 Hz, 1H, NC*H*CHN), 4.00 (t, J = 7 Hz, 2H, $NC\mathit{H}_2CH_2$), 3.76 (s, 3H, $NC\mathit{H}_3$), 3.44 (s, 3H, $C\mathit{H}_3SO_4$), 1.67 - 1.55 (m, 2H, $CH_2C\mathit{H}_2CH_2$), 1.16 - 1.03 (m, 2H, $CH_2C\mathit{H}_2CH_3$), 0.68 (t,

J = 7 Hz, 3H, CH_2CH_3). δ_C (ppm) (101 MHz, $CDCl_3$) 136.64 (s, N*C*HN), 123.61 (s, NCH*C*HN), 122.31 (s, N*C*HCHN), 53.00 (s, CH_3SO_4), 48.51 (s, NCH_2CH_2), 35.67 (s, NCH_3), 31.41 (s, $CH_2CH_2CH_2$), 18.79 (s, $CH_2CH_2CH_3$), 13.24 (s, CH_2CH_3). *m/z* (ESI^+): 139 ($[C_4C_1im]^+$, 100%), 389 ($\{[C_4C_1im]_2[MeSO_4]\}^+$, 20%). *m/z* ($ESI^-$): 111 ($[MeSO_4]^-$, 100%), 361 ($\{[C_4C_1im][MeSO_4]_2\}^-$, 40%), 611 ($\{[C_4C_1im]_2[MeSO_4]_3\}^-$, 55%). Elemental analysis (expected): C 43.30% (43.18%), H 7.42% (7.25%), N 11.10% (11.19%). ν/cm^{-1} (neat): 3103 (aromatic C−H str., w), 2961 (aliphatic C−H str., w), 1573 (ring def., m), 1466 (aliphatic C−H bend, m), 1221 (asym. S−O str., s), 1169 (asym. S−O str., m), 1059 (O−C str., m), 1007 (sym. O−S, s), 731 (S−O str., s).

1-Butyl-3-methylimidazolium dimethyl phosphate. To a solution of 1-butylimidazole (105.27 g, 848 mmol) in toluene (100 ml), trimethyl phosphate (113.19 g, 808 mmol) was added dropwise. The resulting mixture was stirred at 80°C for 22 hours, and subsequently was cooled in the freezer at -23 °C for 2.5 hours. The clear, colourless liquid remained as one phase, and was therefore stirred at 90 °C for a further 64 hours. The yellow liquid was cooled in the freezer at -23 °C for 2.5 hours. As no phase separation occurred, the liquid was diluted with toluene (400 ml), upon which phase separation did occur. The toluene phase was separated off, and the ionic liquid phase was washed with toluene (3 × 300 ml). The remaining solvent was evaporated under vacuum, to yield 1-butyl-3-methylimidazolium dimethyl phosphate as a light yellow, free-flowing liquid (210.72 g, 94%). δ_H (ppm) (400 MHz, DMSO-d6) 9.64 (s, 1H, NC*H*N), 7.88 (t, J = 2 Hz, 1H, NCHC*H*N), 7.80 (t, J = 2 Hz, 1H, NC*H*CHN), 4.18 (t, J = 7 Hz, 2H, NCH_2CH_2), 3.87 (s, 3H, NCH_3), 3.27 (d, 6H, $(CH_3)_2PO_4$), 1.81 - 1.71 (m, 2H, $CH_2CH_2CH_2$), 1.31 - 1.18 (m, 2H, $CH_2CH_2CH_3$), 0.88 (t, J = 7 Hz, 3H, CH_2CH_3). δ_C (ppm) (101 MHz, DMSO-d6) 137.27 (s, N*C*HN), 123.61 (s, NCH*C*HN), 122.32 (s, N*C*HCHN), 51.24 (d, J = 6 Hz, $(CH_3)_2PO_4$), 48.36 (s, NCH_2CH_2), 35.56 (s, NCH_3), 31.43 (s, $CH_2CH_2CH_2$), 18.77 (s, $CH_2CH_2CH_3$), 13.25 (s, CH_2CH_3). δ_P (ppm) (162 MHz, DMSO-d6) 1.51 (s, $(CH_3)_2PO_4$). *m/z* (ESI^+): 139 ($[C_4C_1im]^+$, 100%), 403 ($\{[C_4C_1im]_2[Me_2PO_4]\}^+$, 40%). *m/z* ($ESI^-$): 125 ($[Me_2PO_4]^-$, 100%). Elemental analysis (expected): C 28.75% (28.64%), H 3.66% (3.61%), N 9.89% (10.02%). ν/cm^{-1} (neat): 3049 (aromatic C−H str., wb), 2959 2939 2875 2835 (aliph. C−H str., m), 1569 (aromatic ring def., m), 1465n(aliphatic C−H bend, m), 1243 (asym. P−O str., s), 1178 (sym. O−C str., m), 1092 (asym. O−C str., m), 1044 (sym. P−O str., s), 770 (asym. P−O str., s), 730 (sym P−O str., m).

1-Butyl-1-methylpyrrolidinium *bis*(trifluoromethanesulfonyl)imide. A solution of 1-butyl-1-methylpyrrolidinium chloride (38 g, 214 mmol) in CH_2Cl_2 (150 ml) was added to lithium *bis*(trifluoromethanesulfonyl)imide (70 g, 244 mmol), and the resulting slurry was stirred for 24 hours at room temperature. The white precipitate was filtered off, and the remaining liquid was diluted with CH_2Cl_2 to a total volume of ∼ 750 ml. The diluted solution was subsequently washed with water (7 × 100 ml) until halide free by $AgNO_3$ test. The solvent was removed under vacuum, to yield 1-butyl-1-methylpyrrolidinium *bis*(trifluoromethanesulfonyl)imide as a colorless, free-flowing liquid (72.20 g, 80%). δ_H (ppm) (500 MHz, DMSO-d6 capillary) 3.01 - 2.87 (m, 4H, NCH_2CH_2), 2.79 - 2.69 (m, 2H, NCH_2), 2.44 (s, 3H, NCH_3), 1.69 - 1.58 (m, 4H, $N(CH_2)_2(CH_2)_2$), 1.24 - 1.14 (m, 2H, $NCH_2CH_2CH_2$), 0.88 - 0.77 (m, 2H, $NCH_2CH_2CH_2CH_3$), 0.40 (t, J = 7 Hz, 3H, $NCH_2CH_2CH_2CH_3$). δ_C (ppm) (126 MHz, DMSO-d6 capillary) 118.86 (q, J = 321 Hz, $(CF_3O_2S)_2N$)), 63.07 (s, $N(CH_2)_2$), 63.02 (s, $N(CH_2)_2(CH_2)_2$), 46.69 (s, NCH_3), 24.18 (s, $NCH_2CH_2CH_2CH_3$), 20.04 (s, $NCH_2CH_2CH_2CH_3$), 18.14 (s, $NCH_2CH_2CH_2CH_3$), 11.56 (s, $NCH_2CH_2CH_2CH_3$). *m/z* (ESI^+): 142 ($[C_4C_1pyrr]^+$, 45%), 564 ($\{[C_4C_1pyrr]_2[NTf_2]\}^+$, 100%). *m/z* ($ESI^-$): 280 ($[NTf_2]^-$, 100%), 702 ($\{[C_4C_1pyrr][NTf_2]_2\}^-$, 10%). Elemental analysis (expected): C 31.38% (31.28%), H 4.69% (4.77%), N 6.55% (6.63%). ν/cm^{-1} (neat): 2970 (ring C−H str., w), 2882 (aliphatic C−H str., w), 1467 (C−H bend, m), 1348 1330 (asym. S−O str., s), 1180 1135 (asym. C−F str., s), 1053 (asym S−N str., s), 928 (ring breathing vibration, w).

1-Butyl-1-methylpyrrolidinium dimethyl phosphate. To a solution of 1-butylpyrrolidine (90.95 g, 715 mmol) in toluene (100 ml), trimethyl phosphate (72.0 g, 616 mmol) was added dropwise. The resulting mixture was stirred at 80 °C for 24 hours. The resulting liquid was diluted with toluene (400 ml), upon which phase separation occurred. The toluene phase was separated off, and the ionic liquid phase was washed with toluene (3 × 300 ml). The remaining solvent was evaporated under vacuum, to yield 1-butyl-1-methylpyrrolidinium dimethyl phosphate as a pale yellow solid (140.66 g, 74%). δ_H (ppm) (400 MHz, DMSO-d6) 3.58 - 3.43 (m, 4H, $N(CH_2)_2(CH_2)_2$), 3.41 - 3.32 (m, 2H, NCH_2), 3.24 (d, J = 10 Hz, 6H, $(CH_3)_2PO_4$), 3.01 (s, 3H, NCH_3), 2.12 - 1.99 (m, 4H, $N(CH_2)_2(CH_2)_2$), 1.72 - 1.61 (m, 2H, $NCH_2CH_2CH_2$), 1.36 - 1.24 (m, 2H, $NCH_2CH_2CH_2CH_3$), 0.91 (t, J = 7 Hz, 3H, $NCH_2CH_2CH_2CH_3$). δ_C (ppm) (101 MHz, DMSO-d6) 63.09 (s, $N(CH_2)_2(CH_2)_2$), 62.55 (s, $N(CH_2)_2(CH_2)_2$), 51.16 (d, J = 6 Hz, $(CH_3)_2PO_4$), 47.13 (s, NCH_3), 24.97 (s,

N$CH_2CH_2CH_2CH_3$), 20.98 (s, $NCH_2CH_2CH_2CH_3$), 19.30 (s, $NCH_2CH_2CH_2CH_3$), 13.49 (s, $NCH_2CH_2CH_2CH_3$). δ_P (ppm) (162 MHz, DMSO-d6) 1.39 (s, $(CH_3)_2PO_4$). *m/z* (ESI$^+$): 142 ($[C_4C_1pyrr]^+$, 100%), 409 ($\{[C_4C_1pyrr]_2[Me_2PO_4]\}^+$, 10%). *m/z* (ESI$^-$): 125 ($[Me_2PO_4]^-$, 20%), 392 ($\{[C_4C_1pyrr][Me_2PO_4]_2\}^-$, 10%). Elemental analysis (expected): C 49.29% (49.43%), H 11.34% (9.80%), N 4.61% (5.24%). ν/cm^{-1} (neat): 2963 2941 (C−H str., mb), 1479 (C−H bend., m), 1248 (asym. P−O str., s), 1179 (sym. O−C str, w), 1093 (asym. O−C str., m), 1044 (sym P−O str., s), 937 (ring breathing, w), 768 (asym. p−O str., s).

1-Methyl-1,8-diazabicyclo[5.4.0]undec-7-enium methyl sulfate. To an ice-cooled solution of 1,8-diazabicyclo[5.4.0]undec-7-ene (DBU) (98.34 g, 646 mmol) in toluene (120 ml), dimethyl sulfate (78.77 g, 625 mmol) was slowly added dropwise. The resulting mixture was stirred for 17 hours, during which time the solution had separated into two phases. The toluene phase was separated off, and the ionic liquid phase was washed with toluene (3 × 150 ml). The remaining solvent was removed under vacuum, to yield 1-methyl-1,8-diazabicyclo[5.4.0]undec-7-enium methyl sulfate as a pale yellow, free-flowing liquid. This liquid solidified upon repeated extraction with toluene, and a white solid was obtained (171.14 g, 98%). δ_H (ppm) (400 MHz, DMSO-d6) 3.67 - 3.59 (m, 2H, $NCH_2C_4H_8N$), 3.47 (t, J = 6 Hz, 2H, $NCH_2C_2H_4N$), 3.42 (t, J = 6 Hz, 2H, $NCH_2C_2H_4N$), 3.37 (s, 3H, CH_3SO_4), 3.20 (s, 3H, NCH_3), 2.88 - 2.80 (m, 2H, $C_qCH_2C_4H_8N$), 2.01 - 1.92 (m, 2H, $NCH_2CH_2CH_2N$), 1.72 - 1.56 (m, 6H, $CH_2C_3H_6CH_2N$). δ_C (ppm) (101 MHz, DMSO-d6) 166.03 (s, C_q), 53.84 (s, $NCH_2C_4H_8$), 52.81 (s, CH_3SO_4), 48.13 (s, $NCH_2C_2H_4N$), 48.08 (s, $NCH_2C_2H_4N$), 40.55 (s, NCH_3), 27.83 (s, $CH_2C_3H_6CH_2$), 27.46 (s, $C_qCH_2C_4H_8N$), 25.65 (s, $CH_2C_3H_6CH_2$), 21.73 (s, $CH_2C_3H_6CH_2$), 19.43 (s, $NCH_2CH_2CH_2N$). *m/z* (ESI$^+$): 167 ($[Me{-}DBU]^+$, 100%), 445 ($\{[Me{-}DBU]_2[MeSO4]\}^+$, 10%). *m/z* (ESI$^-$): 111 ($[MeSO4]^-$, 100%). Elemental analysis (expected): C 47.63% (47.46%), H 7.86% (7.97%), N 9.99% (10.06%). ν/cm^{-1} (neat): 2938 (C−H str., m), 1625 (asym. N−C−N str., s), 1220 (asym. S−O str., s), 1196 (asym. S−O str., m), 1059 (O−C str., m), 1009 (sym. O−S, s), 723 (S−O str., s).

1,2,4-Trimethylpyrazolium trifluoromethanesulfonate. Methyl trifluoromethanesulfonate (50.00 g, 305 mmol) was distilled directly into an ice-cooled flask of 1,3-dimethylpyrazole (100.54 g, 1.05 mol), with vigorous stirring. The resulting liquid was stirred for an hour, and was extracted with toluene, initially in a separation funnel (6 × 100 ml), and subsequently for 24 hours in a continuous extractor. Remaining solvent was removed under vacuum and the product was filtered through a layer of basic alumina, to yield 1,2,4-trimethylpyrazolium trifluoromethanesulfonate. δ_H (ppm) (400 MHz, DMSO-d6) 8.23 (s, 2H, C*H*), 4.05 (s, 6H, NCH_3), 2.08 (s, 3H, CCH_3). δ_C (ppm) (101 MHz, DMSO-d6) 136.38 (s, *C*H), 120.75 (q, J = 323 Hz, CF_3SO_3), 116.62 (s, CCH_3), 36.24 (s, NCH_3), 8.23 (s, CCH_3). *m/z* (ESI$^+$): 111 ($[C_1C_1C_1pz]^+$, 50%), 371 ($\{[C_1C_1C_1pz]_2[OTf]\}^+$, 100%). *m/z* (ESI$^-$): 149 ($[OTf]^-$, 100%), 409 ($\{[C_1C_1C_1pz][OTf]_2\}^-$, 30%). Elemental analysis (expected): C 32.28% (32.31%), H 4.29% (4.26%), N 10.84% (10.76%). ν/cm^{-1} (neat): 3129 (arom. C−h str, w), 3038 2961 (aliphatic C−H str., w), 1451 (C−H bend., m), 1404 1369 (ring str., m), 1254 (asym. S−O stretch, s), 1224 (C−S stretch, s), 1152 (asymm. C−F stretch, s), 1029 (symm. S−O and C−F str., s), 850 (ring C−H bend, m).

Triethanolmethylammonium methyl sulfate. To an ice-cooled biphasic mixture of triethanolamine (74.2 g, 497 mmol) and EtOAc (210 ml), dimethyl sulfate (63.8 g, 506 mmol) was added dropwise with vigorous stirring. The liquid was stirred for a further two hours. The organic phase was separated off, and the ionic liquid phase was extracted with toluene (3 x 50ml). The remaining solvent was removed under vacuum. Residual triethanolamine was removed from the product by continuous extraction with $CHCl_3$ until no starting material could be found. Remaining solvent was removed under vacuum, to yield triethanolmethylammonium methyl sulfate as a pale orange, free-flowing liquid (97.1 g, 71%). δ_H (ppm) (400 MHz, DMSO-d6) 5.20 (s, 3H, O*H*), 3.83 (s, 6H, CH_2OH), 3.56 - 3.47 (m, 6H, CH_2CH_2OH), 3.37 (s, 3H, CH_3SO_4), 3.13 (s, 3H, NCH_3). δ_C (ppm) (101 MHz, DMSO-d6) 64.24 (s, NCH_2CH_2OH), 55.01 (s, CH_2OH), 53.15 (s, CH_3SO_4), 49.73 (s, NCH_3). *m/z* (LSIMS$^+$): 164 ($[(C_2OH)_3C_1N]^+$, 100%). *m/z* (LSIMS$^-$): 111 ($[MeSO_4]^-$, 100%), 386 ($\{[(C_2OH)_3C_1N][MeSO_4]_2\}^-$, 10%). Elemental analysis (expected): C 35.05% (34.90%), H 7.79% (7.69%), N 5.09% (5.09%). ν/cm^{-1} (neat): 3384 (O−H str., mb), 2952 (C−H str, w), 1464 (C−H bend., m), 1204 (asym. S−O str., s), 1056 (O−C str., s), 999 (sym. O−S, s), 757 (S−O str., s).

3. Molar volumes of ionic liquid mixtures

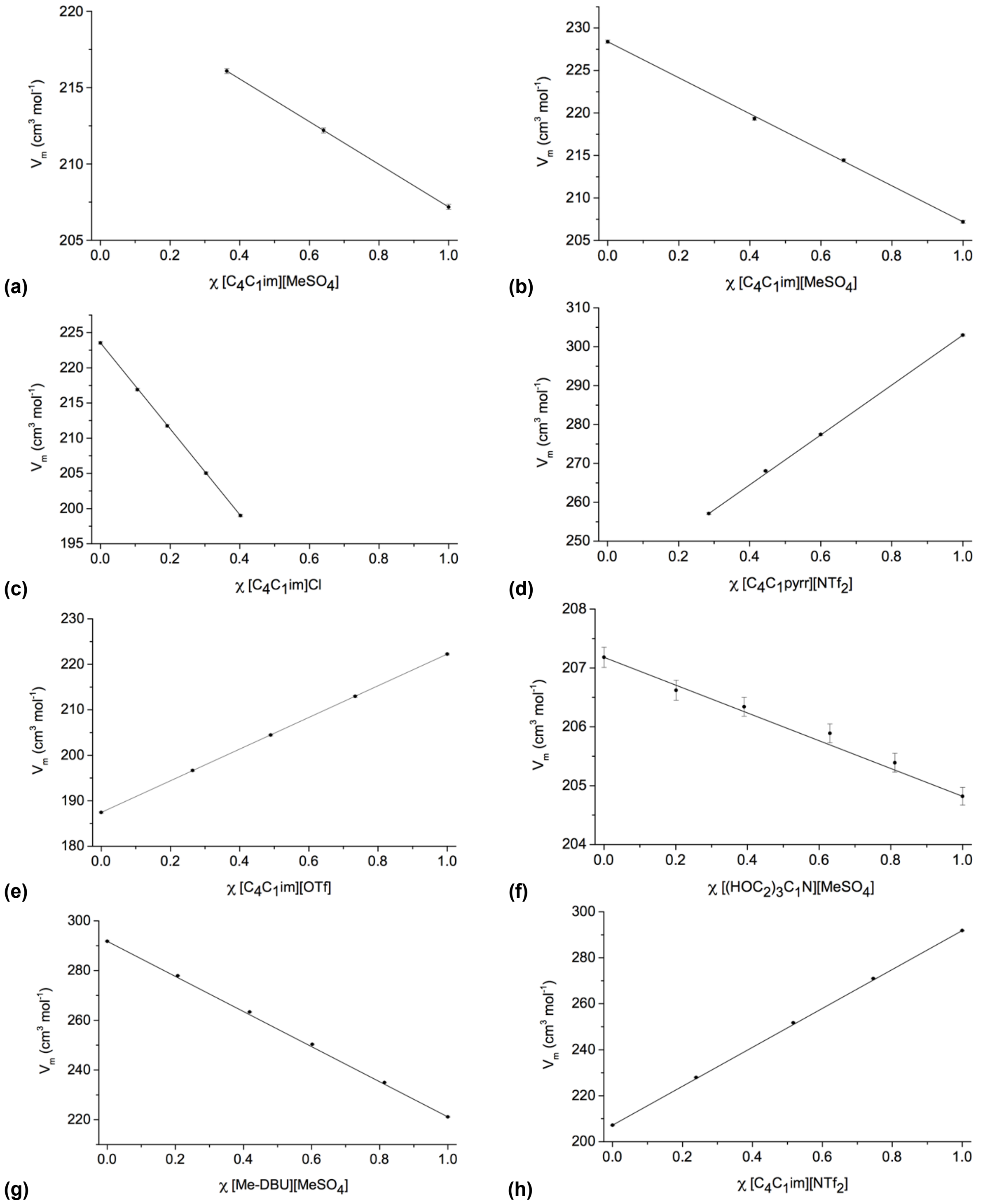

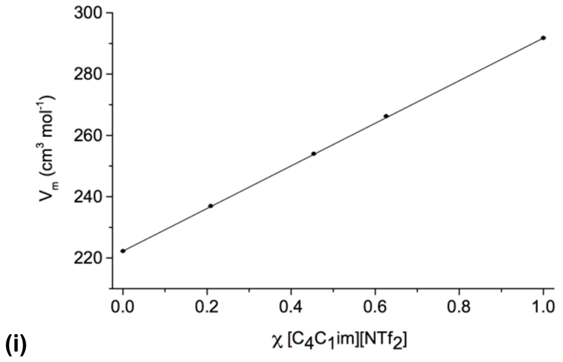

Fig. E2 Molar volume graphs for binary and reciprocal binary ionic liquid mixtures:

(a) $[C_4C_1im][Me\text{-}DBU][MeSO_4]$, **8**.
(b) $[C_4C_1im][MeSO_4][Me_2PO_4]$, **2**.
(c) $[C_4C_1im]Cl[OTf]$, **1**.
(d) $[C_4C_1pyrr][NTf_2][Me_2PO_4]$, **6**.
(e) $[C_4C_1im][C_1C_1C_1pz][OTf]$, **9**.
(f) $[C_4C_1im][(HOC_2)_3C_1N][MeSO_4]$, **4**.
(g) $[C_4C_1im][Me\text{-}DBU][MeSO_4][NTf_2]$, **10**.
(h) $[C_4C_1im][MeSO_4][NTf_2]$, **5**.
(i) $[C_4C_1im][OTf][NTf_2]$, **3**.

Table E2 Molar volume (density) data for binary and reciprocal binary ionic liquid mixtures.

$[C_4C_1im]Cl[OTf]$, **1**.
(χ Cl^-)

χ	M g mol^{-1}	ρ g cm^{-3}	V_m cm^3 mol^{-1}
0	288.29	1.2896	223.55
0.106	276.25	1.2736	216.90
0.192	266.48	1.2585	211.74
0.303	253.92	1.2384	205.03
0.402	242.61	1.2192	199.00

$[C_4C_1im][MeSO_4][Me_2PO_4]$, **2**.
(χ $[MeSO_4]^-$)

χ	M g mol^{-1}	ρ g cm^{-3}	V_m cm^3 mol^{-1}
0	264.26	1.1570	228.40
0.413	258.50	1.1786	219.33
0.664	255.00	1.1892	214.43
1	250.32	1.2082	207.18

$[C_4C_1im][OTf][NTf_2]$, **3**.
(χ $[NTf_2]^-$)

χ	M g mol^{-1}	ρ g cm^{-3}	V_m cm^3 mol^{-1}
0	288.29	1.2972	222.24
0.209	315.68	1.3323	236.95
0.454	347.80	1.3694	253.98
0.626	370.34	1.3910	266.24
1	419.36	1.4371	291.81

$[C_4C_1im][(HOC_2)_3C_1N][MeSO_4]$, **4**.
(χ $[(HOC_2)_3C_1N]^+$)

χ	M g mol^{-1}	ρ g cm^{-3}	V_m cm^3 mol^{-1}
0	250.32	1.2082	207.18
0.201	255.35	1.2358	206.62
0.391	260.10	1.2605	206.34
0.630	266.07	1.2923	205.89
0.811	270.60	1.3175	205.39
1	275.32	1.3442	204.82

$[C_4C_1im][MeSO_4][NTf_2]$, **5**.
(χ $[NTf_2]^-$)

χ	M g mol^{-1}	ρ g cm^{-3}	V_m cm^3 mol^{-1}
0	250.32	1.2082	207.18
0.239	290.72	1.2753	227.96
0.517	337.71	1.3416	251.72
0.746	376.42	1.3892	270.96
1	419.36	1.4371	291.81

$[C_4C_1pyrr][NTf_2][Me_2PO_4]$, **6**.
(χ $[NTf_2]^-$)

χ	M g mol^{-1}	ρ g cm^{-3}	V_m cm^3 mol^{-1}
0	267.30	(1.1176)	(239.17)
0.285	311.51	1.2117	257.08
0.445	336.32	1.2547	268.05
0.600	360.37	1.2990	277.42
1	422.41	1.3941	303.00

$[C_4C_1im][NTf_2][Me_2PO_4]$, **7**.
(χ $[NTf_2]^-$)

χ	M g mol^{-1}	ρ g cm^{-3}	V_m cm^3 mol^{-1}
0	264.26	1.1588	228.05
0.100	279.79	1.1926	234.61
0.150	287.51	1.2086	237.89
0.288	308.95	1.2512	246.92
0.418	329.09	1.2892	255.27
0.500	341.82	1.3119	260.55
0.540	347.95	1.3227	263.06
0.663	367.03	1.3552	270.83
0.776	384.58	1.3834	277.99
0.885	401.52	1.4096	284.84
1	419.36	1.4362	291.99

$[C_4C_1im][Me\text{-}DBU][MeSO_4]$, **8**.
(χ $[C_4C_1im]^+$)

χ	M g mol^{-1}	ρ g cm^{-3}	V_m cm^3 mol^{-1}
0	278.37	(1.2587)	(221.16)
0.363	268.19	1.2411	216.09
0.641	260.39	1.2271	212.20
1	250.32	1.2082	207.18

$[C_4C_1im][C_1C_1C_1pz][OTf]$, **9**.
(χ $[C_4C_1im]^+$)

χ	M g mol^{-1}	ρ g cm^{-3}	V_m cm^3 mol^{-1}
0	260.23	1.3884	187.43
0.264	267.65	1.3610	196.66
0.490	273.98	1.3402	204.44
0.734	280.83	1.3188	212.94
1	288.29	1.2972	222.24

$[C_4C_1im][Me\text{-}DBU][MeSO_4][NTf_2]$, **10**.
(χ $[Me\text{-}DBU][MeSO_4]$)

χ	M g mol^{-1}	ρ g cm^{-3}	V_m cm^3 mol^{-1}
0	419.36	1.4371	291.81
0.207	390.11	1.4038	277.90
0.418	360.42	1.3686	263.35
0.602	334.52	1.3363	250.33
0.814	304.59	1.2963	234.97
1	278.37	(1.2587)	(221.16)

$[C_4C_1im][C_4C_1pyrr][OTf][NTf_2]$, **11**.
(χ $[C_4C_1im][OTf]$)

χ	M g mol^{-1}	ρ g cm^{-3}	V_m cm^3 mol^{-1}
0	422.41	1.3941	303.00
0.233	391.16	1.3761	284.25
0.377	371.85	1.3643	272.55
0.602	341.67	1.3425	254.50
0.787	316.86	1.3237	239.37
0.881	304.25	1.3118	231.93
1	288.29	1.2972	222.24

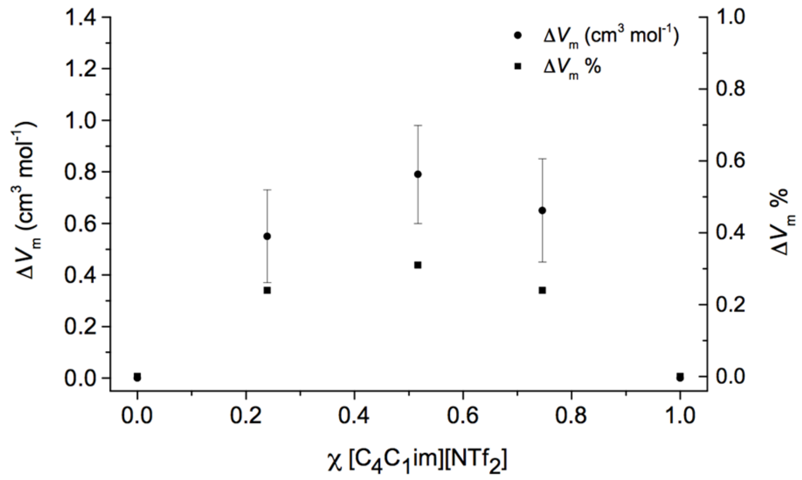

Fig. E3 Excess molar volume graph for $[C_4C_1im][MeSO_4][NTf_2]$, **5**.

Table E3 Excess molar volume data for $[C_4C_1im][MeSO_4][NTf_2]$, **5,** $[C_4C_1im][NTf_2][Me_2PO_4]$, **7**, and $[C_4C_1im]$[Me-DBU]-$[MeSO_4][NTf_2]$, **10**.

$[C_4C_1im][MeSO_4][NTf_2]$, **5**

χ $[NTf_2]^-$	V_m ($cm^3\ mol^{-1}$)	ΔV_m ($cm^3\ mol^{-1}$)	Error ($cm^3\ mol^{-1}$)	ΔV_m %
0	207.18	0	0	0
0.239	227.96	0.55	0.18	0.24
0.517	251.72	0.79	0.19	0.31
0.746	270.96	0.65	0.20	0.24
1	291.81	0	0	0

$[C_4C_1im][NTf_2][Me_2PO_4]$, **7**

χ $[NTf_2]^-$	V_m ($cm^3\ mol^{-1}$)	ΔV_m ($cm^3\ mol^{-1}$)	Error ($cm^3\ mol^{-1}$)	ΔV_m %
0	228.05	0	0	0
0.100	234.61	0.15	0.20	0.07
0.150	237.89	0.25	0.20	0.11
0.288	246.92	0.45	0.20	0.18
0.418	255.27	0.49	0.20	0.19
0.500	260.55	0.53	0.20	0.20
0.540	263.06	0.51	0.20	0.19
0.663	270.83	0.41	0.20	0.15
0.776	277.99	0.34	0.20	0.12
0.885	284.84	0.20	0.20	0.07
1	291.99	0	0	0

$[C_4C_1im]$[Me-DBU]$[MeSO_4][NTf_2]$, **10**

χ [Me-DBU]$[MeSO_4]$	V_m ($cm^3\ mol^{-1}$)	ΔV_m ($cm^3\ mol^{-1}$)	Error ($cm^3\ mol^{-1}$)	ΔV_m %
0	291.81	0	0	0
0.207	277.90	0.71	0.20	0.26
0.418	263.35	1.07	0.19	0.41
0.602	250.33	1.05	0.19	0.42
0.814	234.97	0.67	0.18	0.29
1	221.16	0	0	0

4. Phase behaviour / glass transition temperatures of ionic liquid mixtures

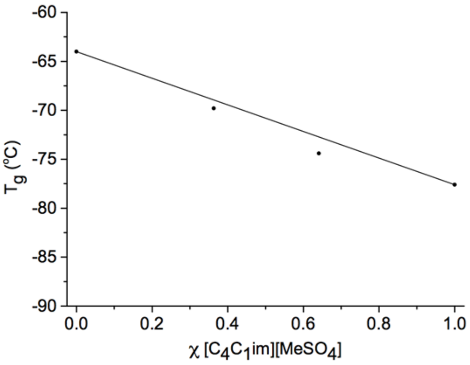

(a)

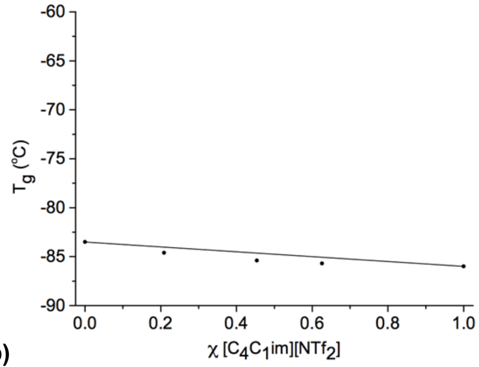

(b)

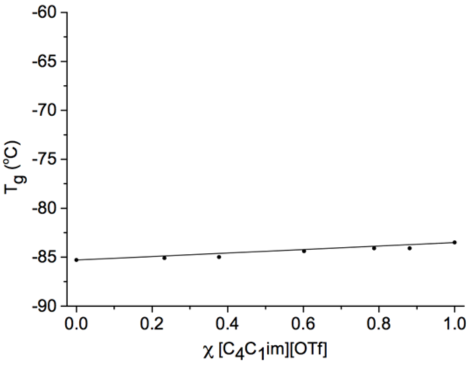

(c)

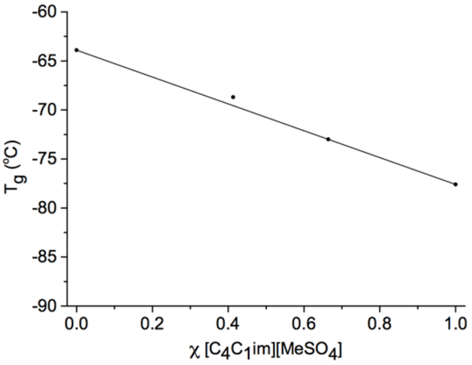

(d)

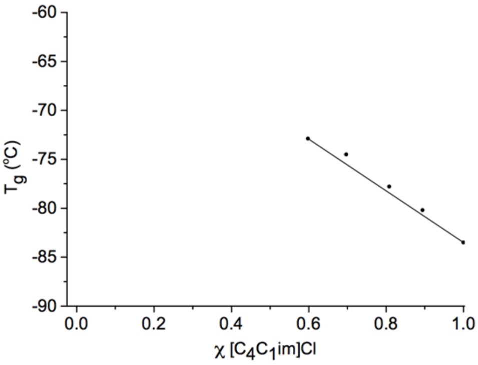

(e)

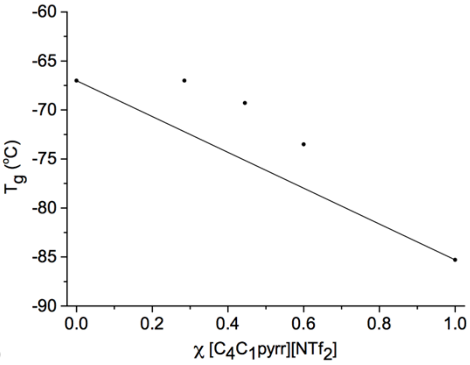

(f)

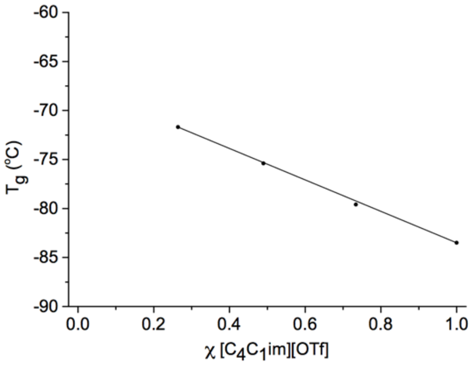

(g)

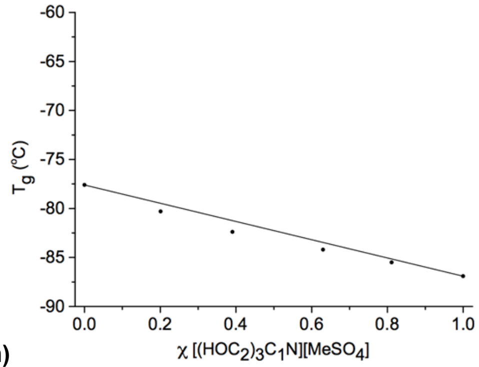

(h)

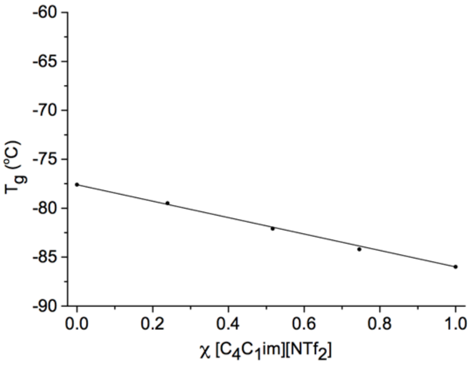

(i)

Fig. E4 Glass transition temperatures (T_g) for binary and reciprocal binary ionic liquid mixtures:

(a) $[C_4C_1im][Me\text{-}DBU][MeSO_4]$, **8**.
(b) $[C_4C_1im][OTf][NTf_2]$, **3**.
(c) $[C_4C_1im][C_4C_1pyrr][OTf][NTf_2]$, **11**.
(d) $[C_4C_1im][MeSO_4][Me_2PO_4]$, **2**.
(e) $[C_4C_1im]Cl[OTf]$, **1**.
(f) $[C_4C_1pyrr][NTf_2][Me_2PO_4]$, **6**.
(g) $[C_4C_1im][C_1C_1C_1pz][OTf]$, **9**.
(h) $[C_4C_1im][(HOC_2)_3C_1N][MeSO_4]$, **4**.
(i) $[C_4C_1im][MeSO_4][NTf_2]$, **5**.

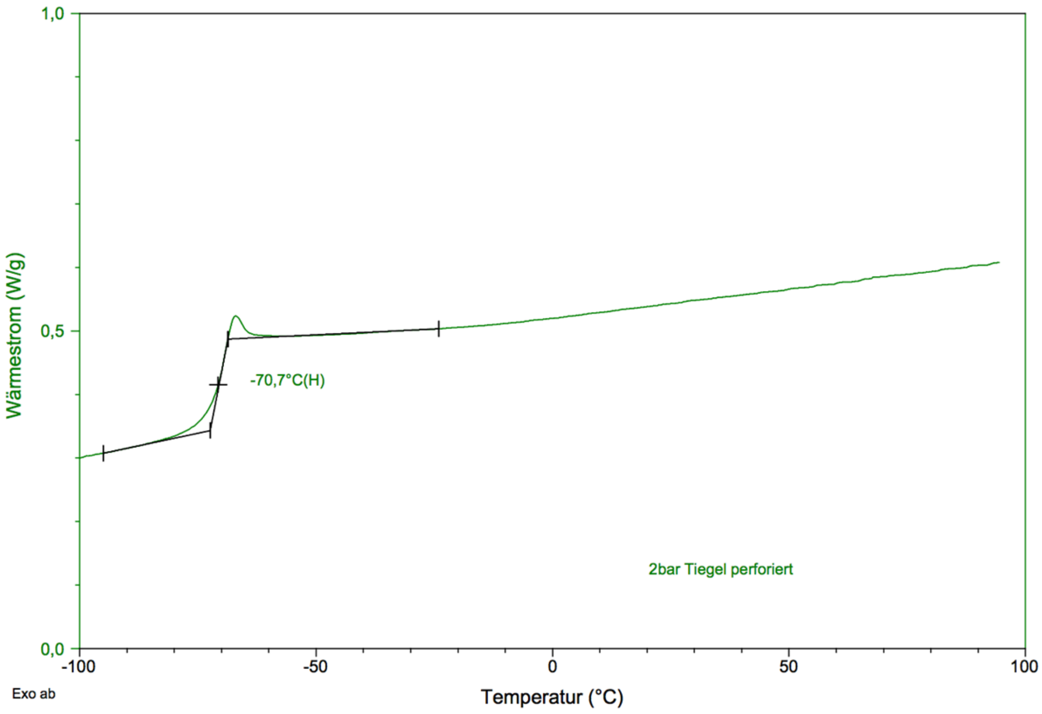

(j)

Fig. E4 continued Example DSC trace for the ionic liquid mixture $[C_4C_1im][NTf_2]_{0.5}[Me_2PO_4]_{0.5}$, **7**. The glass transition temperature values, T_g, are calculated by manual peak analysis, therefore precise errors are difficult to assertain. An error value of ± 0.5 °C is tentatively assigned for all T_g values.

Table E4 Glass transition temperature (T_g) data for binary and reciprocal binary ionic liquid mixtures.

$[C_4C_1im]Cl[OTf]$, **1**

χ Cl^-	T_g (°C)
0	-83.5
0.106	-80.2
0.192	-77.8
0.303	-74.5
0.402	-72.9

$[C_4C_1im][MeSO_4][Me_2PO_4]$, **2**

χ $[Me_2PO_4]^-$	T_g (°C)
0	-77.6
0.336	-73.0
0.587	-68.7
1	-63.9

$[C_4C_1im][OTf][NTf_2]$, **3**

χ $[NTf_2]^-$	T_g (°C)
0	-83.5
0.209	-84.6
0.454	-85.4
0.626	-85.7
1	-86.0

$[C_4C_1im][(HOC_2)_3C_1N][MeSO_4]$, **4**

χ $[(HOC_2)_3C_1N]^+$	T_g (°C)
0	-77.6
0.201	-80.3
0.391	-82.4
0.630	-84.2
0.811	-85.5
1	-86.9

$[C_4C_1im][MeSO_4][NTf_2]$, **5**

χ $[NTf_2]^-$	T_g (°C)
0	-77.6
0.239	-79.5
0.517	-82.1
0.746	-84.2
1	-86.0

$[C_4C_1pyrr][NTf_2][Me_2PO_4]$, **6**

χ $[NTf_2]^-$	T_g (°C)
0	-67.0
0.285	-67.0
0.445	-69.3
0.600	-73.5
1	-85.3

$[C_4C_1im][NTf_2][Me_2PO_4]$, **7**

χ $[NTf_2]^-$	T_g (°C)
0	-64.7
0.150	-65.7
0.271	-67.1
0.387	-67.9
0.500	-70.7
0.690	-77.5
0.838	-81.6
1	-86.0

$[C_4C_1im][Me\text{-}DBU][MeSO_4]$, **8**

χ $[Me\text{-}DBU]^+$	T_g (°C)
0	-77.6
0.359	-74.4
0.637	-69.8
1	-64.0

$[C_4C_1im][C_1C_1C_1pz][OTf]$, **9**

χ $[C_1C_1C_1pz]^+$	T_g (°C)
0	-83.5
0.266	-79.6
0.510	-75.4
0.736	-71.7
1	/

$[C_4C_1im][Me\text{-}DBU][MeSO_4][NTf_2]$, **10**

χ [Me-DBU][MeSO4]	T_g (°C)
0	-86.0
0.207	-82.4
0.418	-77.8
0.602	-73.4
0.814	-68.4
1	-64.0

$[C_4C_1im][C_4C_1pyrr][OTf][NTf_2]$, **11**

χ $[C_4C_1pyrr][NTf_2]$	T_g (°C)
0	-83.5
0.119	-84.1
0.213	-84.1
0.398	-84.4
0.623	-85.0
0.767	-85.1
1	-85.3

5. Viscosities of ionic liquid mixtures

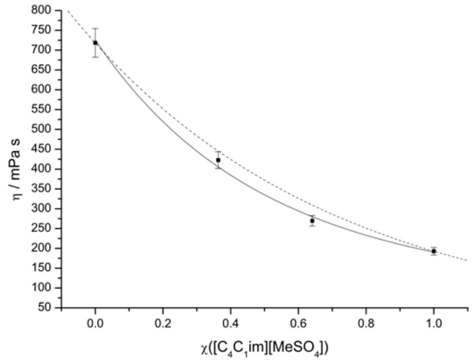

(a)

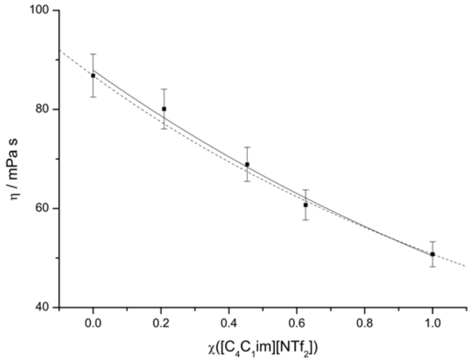

(b)

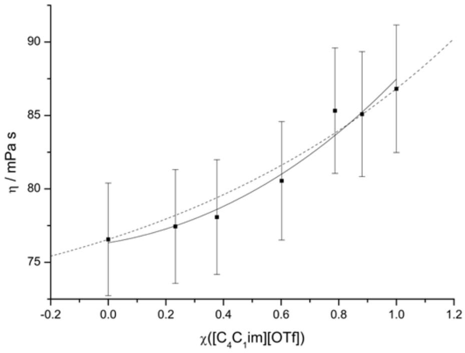

(c)

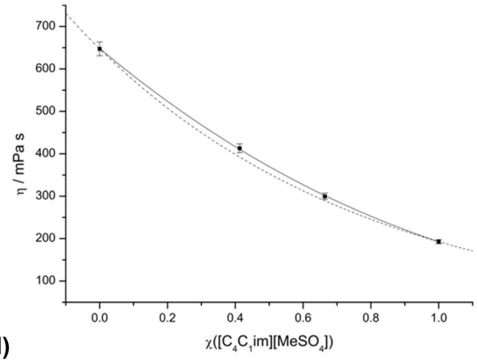

(d)

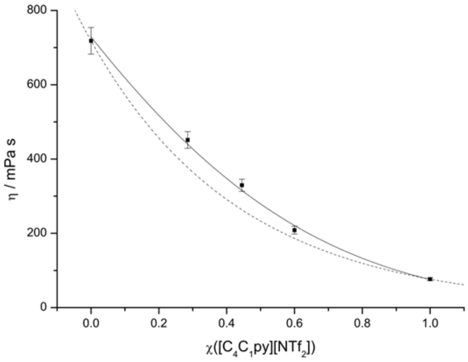

(e)

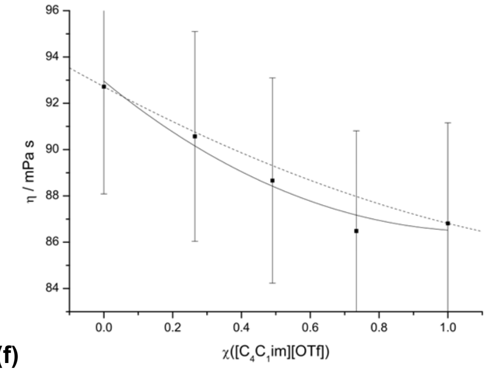

(f)

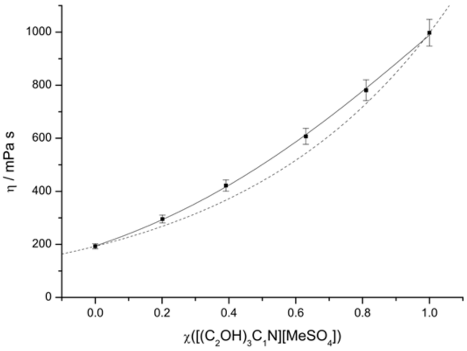

(g)

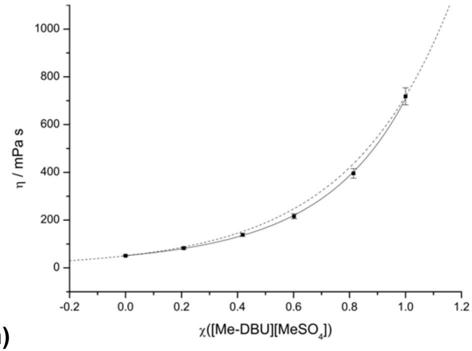

(h)

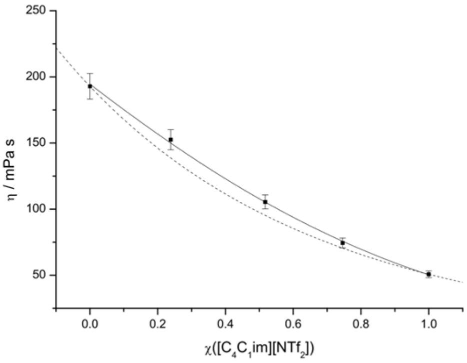

(i)

Fig. E5 Viscosities for binary and reciprocal binary ionic liquid mixtures:

(a) $[C_4C_1im][Me\text{-}DBU][MeSO_4]$, **8**.
(b) $[C_4C_1im][OTf][NTf_2]$, **3**.
(c) $[C_4C_1im][C_4C_1pyrr][OTf][NTf_2]$, **11**.
(d) $[C_4C_1im][MeSO_4][Me_2PO_4]$, **2**.
(e) $[C_4C_1pyrr][NTf_2][Me_2PO_4]$, **6**.
(f) $[C_4C_1im][C_1C_1C_1pz][OTf]$, **9**.
(g) $[C_4C_1im][(HOC_2)_3C_1N][MeSO_4]$, **4**.
(h) $[C_4C_1im][Me\text{-}DBU][MeSO_4][NTf_2]$, **10**.
(i) $[C_4C_1im][MeSO_4][NTf_2]$, **5**.

Graphs were constructed using a logarithmic fitting, in the Origin Pro v. 8.5 package of programmes.

6. Conductivities of ionic liquid mixtures

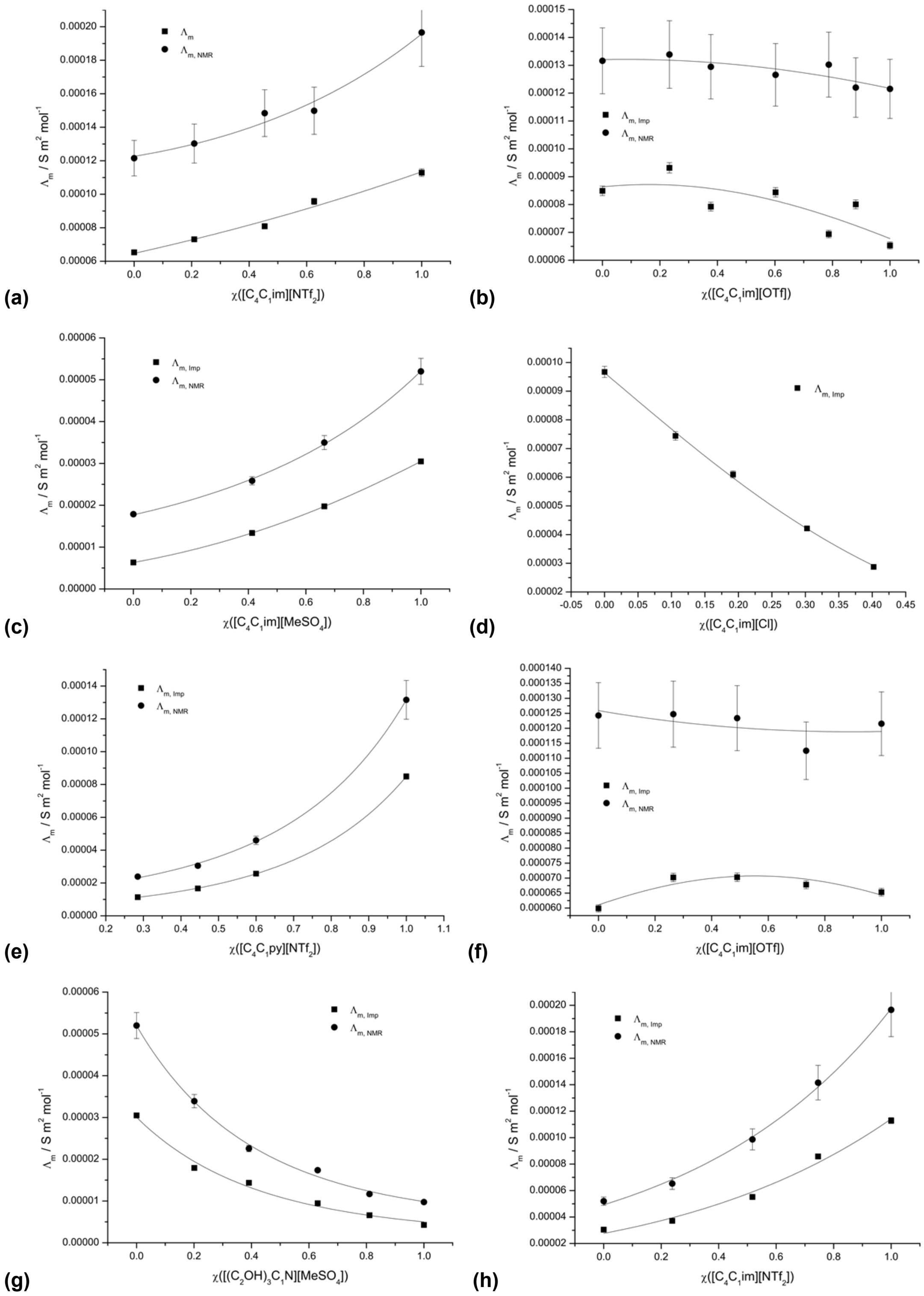

Fig. E6 Conductivities for binary and reciprocal binary ionic liquid mixtures:

(a) $[C_4C_1im][OTf][NTf_2]$, **3**.
(b) $[C_4C_1im][C_4C_1pyrr][OTf][NTf_2]$, **11**.
(c) $[C_4C_1im][MeSO_4][Me_2PO_4]$, **2**.
(d) $[C_4C_1im]Cl[OTf]$, **1**.
(e) $[C_4C_1pyrr][NTf_2][Me_2PO_4]$, **6**.
(f) $[C_4C_1im][C_1C_1C_1pz][OTf]$, **9**.
(g) $[C_4C_1im][(HOC_2)_3C_1N][MeSO_4]$, **4**.
(h) $[C_4C_1im][MeSO_4][NTf_2]$, **5**.

Graphs were constructed using a logarithmic fitting, in the Origin Pro v. 8.5 package of programmes.

7. Walden relationship of viscosity and conductivity for ionic liquid mixtures

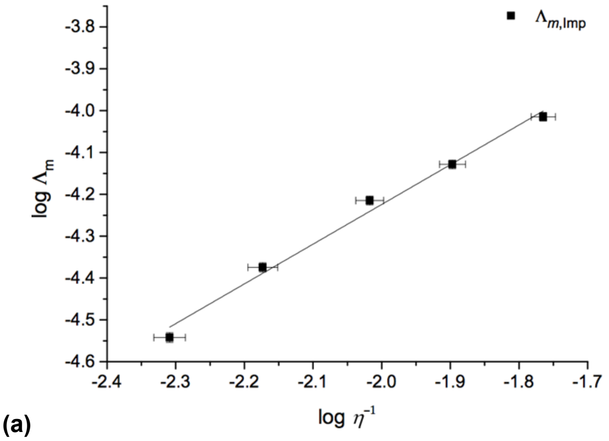

(a)

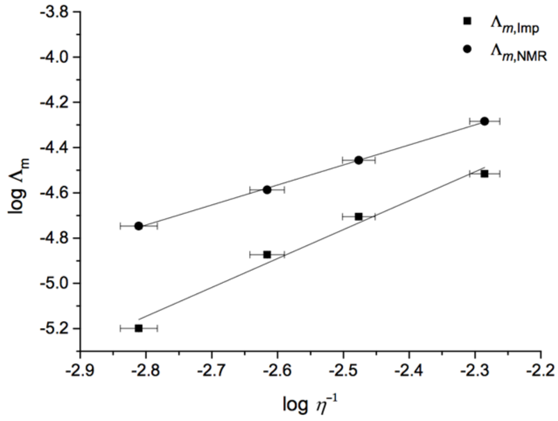

(b)

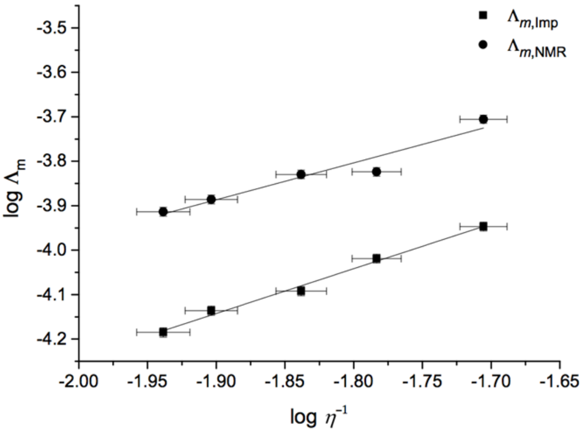

(c)

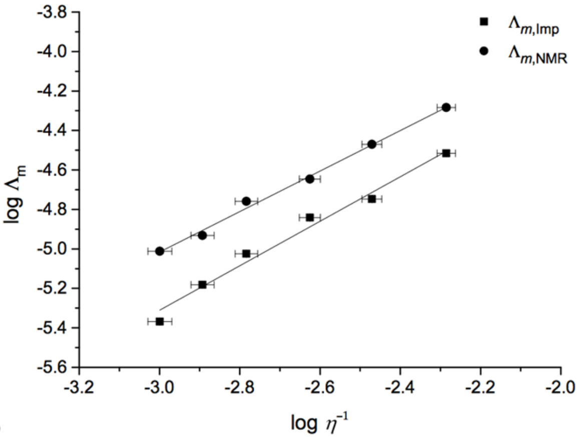

(d)

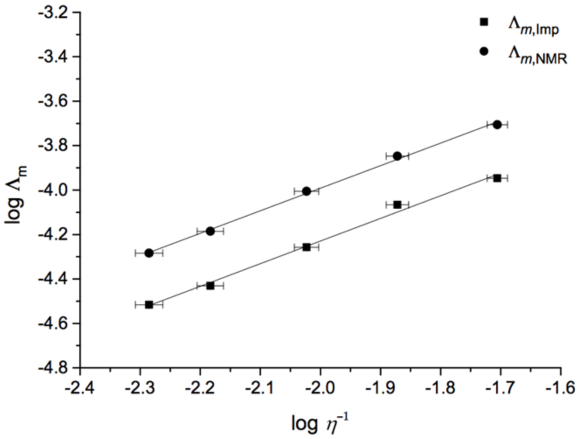

(e)

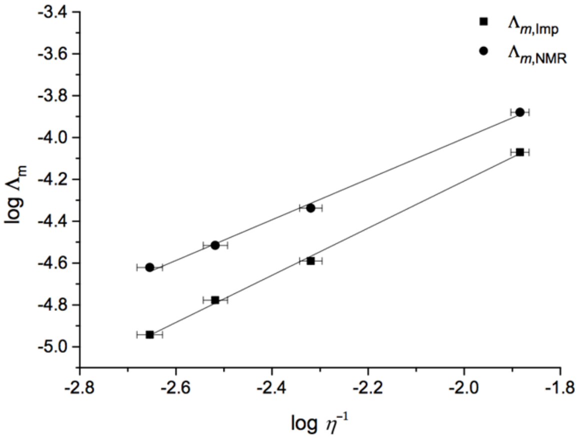

(f)

Fig. E7 Walden plots plotting log inverse viscosity (log (η^{-1}, mPa^{-1} s^{-1}), and the log conductivity, log (Λ_m, Sm^2 mol^{-1}), for ionic liquid mixture series':

(a) $[C_4C_1im]Cl[OTf]$, **1**.
(b) $[C_4C_1im][MeSO_4][Me_2PO_4]$, **2**.
(c) $[C_4C_1im][OTf][NTf_2]$, **3**.
(d) $[C_4C_1im][(HOC_2)_3C_1N][MeSO_4]$, **4**.
(e) $[C_4C_1im][MeSO_4][NTf_2]$, **5**.
(f) $[C_4C_1pyrr][NTf_2][Me_2PO_4]$, **6**.

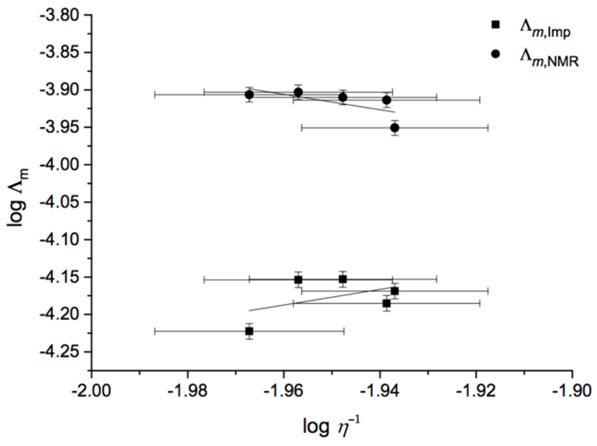

(g)

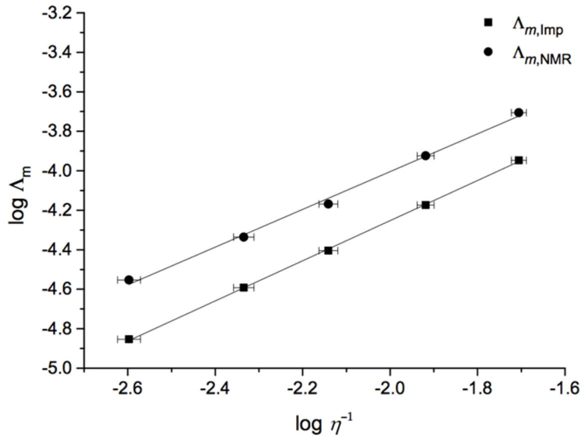

(h)

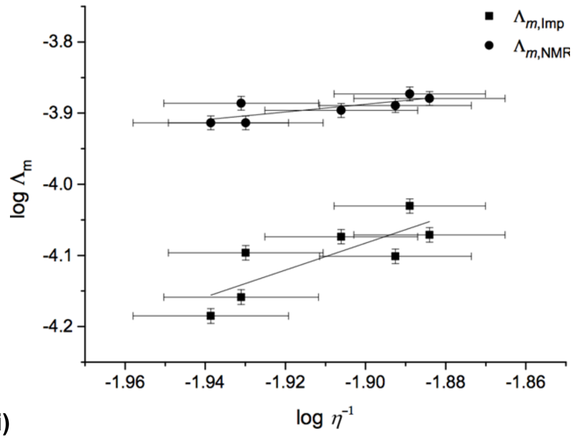

(i)

Fig. E7 continued Walden plots plotting log inverse viscosity (log (η^{-1}, mPa^{-1} s^{-1}), and the log conductivity, log (Λ_m, Sm^2 mol^{-1}), for ionic liquid mixture series':

(g) $[C_4C_1im][C_1C_1C_1pz][OTf]$, **9**.
(h) $[C_4C_1im][Me\text{-}DBU][MeSO_4][NTf_2]$, **10**
(i) $[C_4C_1im][C_4C_1pyrr][OTf][NTf_2]$, **11**.

Table E5 Viscosity and conductivity data for binary and reciprocal binary ionic liquid mixtures.

$[C_4C_1im]Cl[OTf]$, **1** (χ Cl^-)

χ	η mPa s	$\Lambda_{m,Imp}$ Sm^2/mol	$\Lambda_{m,NMR}$ Sm^2/mol
0	58.16	9.67E-05	N/A
0.106	78.87	7.44E-05	N/A
0.192	104.10	6.10E-05	N/A
0.303	149.00	4.22E-05	N/A
0.402	203.67	2.87E-05	N/A
f	-0.4866	2.58834	

$[C_4C_1im][MeSO_4][Me_2PO_4]$, **2** ($\chi$ $[MeSO_4]^-$)

χ	η mPa s	$\Lambda_{m,Imp}$ Sm^2/mol	$\Lambda_{m,NMR}$ Sm^2/mol
1	192.85	3.05E-05	5.20E-05
0	647.30	6.33E-06	1.79E-05
0.413	412.97	1.34E-05	2.59E-05
0.664	299.63	1.97E-05	3.50E-05
f	0.197994	0.4516	-0.22123

$[C_4C_1im][OTf][NTf_2]$, **3** (χ $[NTf_2]^-$)

χ	η mPa s	$\Lambda_{m,Imp}$ Sm^2/mol	$\Lambda_{m,NMR}$ Sm^2/mol
0	86.82	6.53E-05	1.22E-04
1	50.76	1.13E-04	1.97E-04
0.454	68.90	8.09E-05	1.48E-04
0.209	80.09	7.31E-05	1.30E-04
0.626	60.71	9.57E-05	1.50E-04
f	0.003694	0.03437	-0.25742

$[C_4C_1im][(HOC_2)_3C_1N][MeSO_4]$, **4** ($\chi$ $[(HOC_2)_3C_1N]^+$)

χ	η mPa s	$\Lambda_{m,Imp}$ Sm^2/mol	$\Lambda_{m,NMR}$ Sm^2/mol
0	192.85	3.05E-05	5.20E-05
1	997.77	4.28E-06	9.74E-06
0.391	422.05	1.44E-05	2.26E-05
0.630	607.35	9.46E-06	1.74E-05
0.811	781.10	6.59E-06	1.17E-05
0.201	295.55	1.79E-05	3.39E-05
f	0.575614	-0.49877	-0.65208

$[C_4C_1im][MeSO_4][NTf_2]$, **5** ($\chi$ $[NTf_2]^-$)

χ	η mPa s	$\Lambda_{m,Imp}$ Sm^2/mol	$\Lambda_{m,NMR}$ Sm^2/mol
0	192.85	3.05E-05	5.20E-05
1	50.76	1.13E-04	1.97E-04
0.239	152.50	3.71E-05	6.53E-05
0.517	105.45	5.52E-05	9.87E-05
0.746	74.45	8.58E-05	1.42E-04
f	0.369141	0.10605	-0.01906

$[C_4C_1pyrr][NTf_2][Me_2PO_4]$, **6** ($\chi$ $[NTf_2]^-$)

χ	η mPa s	$\Lambda_{m,Imp}$ Sm^2/mol	$\Lambda_{m,NMR}$ Sm^2/mol
0	718.20	N/A	N/A
1	76.57	8.49E-05	1.32E-04
0.285	451.27	1.14E-05	2.39E-05
0.445	329.57	1.67E-05	3.05E-05
0.600	208.57	2.57E-05	4.60E-05
f	0.745323	-0.58486	-0.80026

$[C_4C_1im][NTf_2][Me_2PO_4]$, **7** ($\chi$ $[NTf_2]^-$)

χ	η mPa s	$\Lambda_{m,Imp}$ Sm^2/mol	$\Lambda_{m,NMR}$ Sm^2/mol
1	50.76	1.13E-04	1.97E-04
0	647.30	6.33E-06	1.79E-05
0.387	327.63	1.68E-05	N/A
0.271	N/A	1.04E-05	2.46E-05
0.690	119.67	5.58E-05	8.43E-05
0.838	80.50	8.04E-05	1.27E-04
f	0.513739	2.16714	-0.20031

$[C_4C_1im][Me\text{-}DBU][MeSO_4]$, **8** ($\chi$ $[C_4C_1im]^+$)

χ	η mPa s	$\Lambda_{m,Imp}$ Sm^2/mol	$\Lambda_{m,NMR}$ Sm^2/mol
1	192.85	3.05E-05	5.20E-05
0	718.33	N/A	N/A
0.363	422.71	N/A	2.66E-05
0.641	269.73	2.60E-05	3.57E-05
f	-0.45092		

$[C_4C_1im][C_1C_1C_1pz][OTf]$, **9** ($\chi$ $[C_4C_1im]^+$)

χ	η mPa s	$\Lambda_{m,Imp}$ Sm^2/mol	$\Lambda_{m,NMR}$ Sm^2/mol
1	86.82	6.53E-05	1.22E-04
0	92.72	5.99E-05	1.24E-04
0.264	90.57	7.02E-05	1.25E-04
0.734	86.48	6.78E-05	1.12E-04
0.490	88.66	7.03E-05	1.23E-04
f	-0.06416	0.51506	-0.07801

$[C_4C_1im][Me\text{-}DBU][MeSO_4][NTf_2]$, **10** ($\chi$ $[Me\text{-}DBU][MeSO_4]$)

χ	η mPa s	$\Lambda_{m,Imp}$ Sm^2/mol	$\Lambda_{m,NMR}$ Sm^2/mol
0	50.76	1.13E-04	1.97E-04
1	718.33	N/A	N/A
0.814	395.65	1.40E-05	2.80E-05
0.602	216.10	2.56E-05	4.61E-05
0.418	138.35	3.94E-05	6.78E-05
0.207	82.95	6.70E-05	1.19E-04
f	-0.55309	0.03783	-0.1081

$[C_4C_1im][C_4C_1pyrr][OTf][NTf_2]$, **11** ($\chi$ $[C_4C_1im][OTf]$)

χ	η mPa s	$\Lambda_{m,Imp}$ Sm^2/mol	$\Lambda_{m,NMR}$ Sm^2/mol
1	86.82	6.53E-05	1.22E-04
0	76.57	8.49E-05	1.32E-04
0.377	78.08	7.92E-05	1.29E-04
0.787	85.32	6.94E-05	1.30E-04
0.881	85.09	8.01E-05	1.22E-04
0.602	80.55	8.44E-05	1.27E-04
0.233	77.44	9.32E-05	1.34E-04
f	-0.10109	0.38719	0.10478

8. Thermal stabilities of ionic liquid mixtures

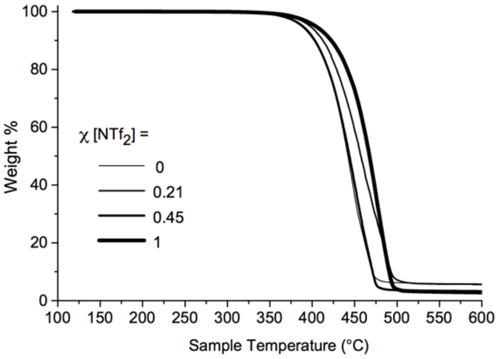

(a)

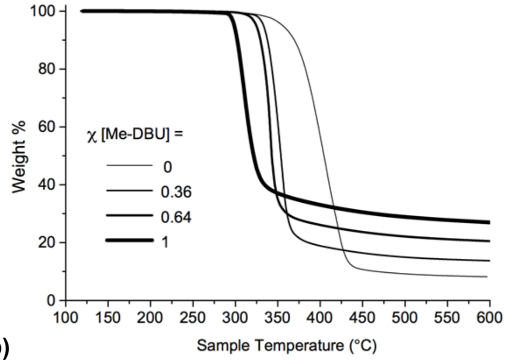

(b)

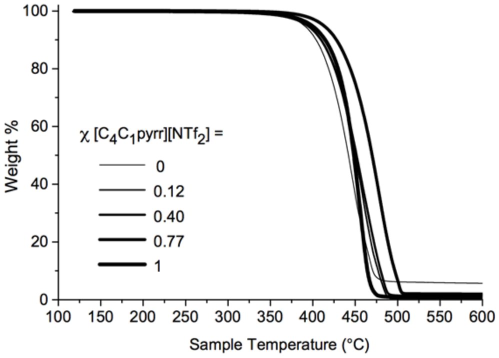

(c)

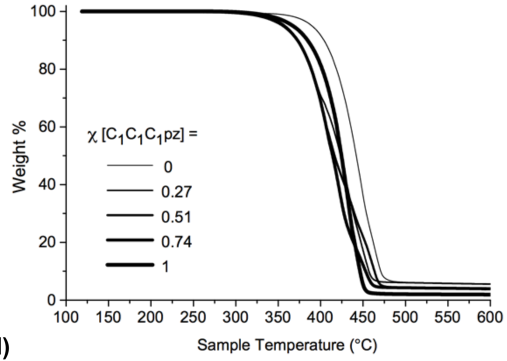

(d)

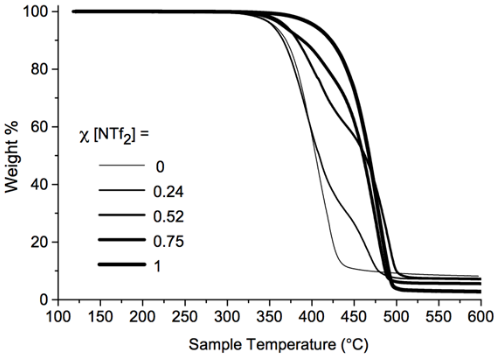

(e)

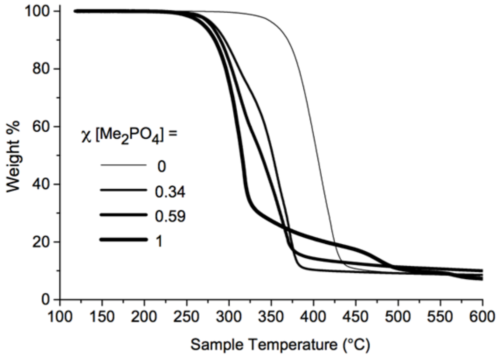

(f)

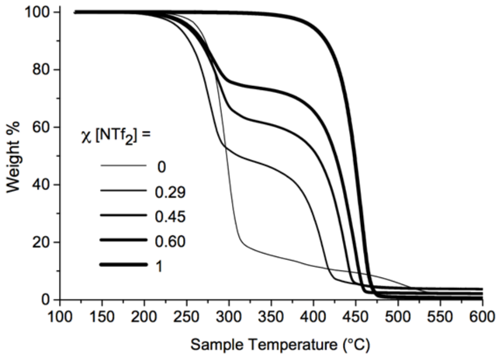

(g)

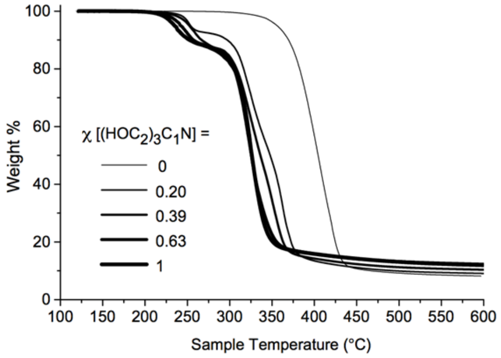

(h)

Fig. E8 TGA thermographs for binary and reciprocal binary ionic liquid mixtures:

(a) [C_4C_1im][OTf][NTf_2], **3**.
(b) [C_4C_1im][Me-DBU][$MeSO_4$], **8**.
(c) [C_4C_1im][C_4C_1pyrr][OTf][NTf_2], **11**.
(d) [C_4C_1im][$C_1C_1C_1$pz][OTf], **9**.
(e) [C_4C_1im][$MeSO_4$][NTf_2], **5**.
(f) [C_4C_1im][$MeSO_4$][Me_2PO_4], **2**.
(g) [C_4C_1pyrr][NTf_2][Me_2PO_4], **6**.
(h) [C_4C_1im][$(HOC_2)_3C_1N$][$MeSO_4$], **4**.

Table E6 T_{onset} values for neat ionic liquids and ionic liquid mixtures, measured using temperature-ramped TGA with a heating rate of 10 °C min^{-1} and nitrogen flow rate of 20 ml min^{-1}. For thermographs that exhibit two or more weight loss steps, the T_{onset} refers to the first step.

$[C_4C_1im]Cl[OTf]$, **1**

χ Cl^-	T_{onset} (°C)
0	410
0.106	253
0.192	256
0.303	257
0.402	258
1	255

$[C_4C_1im][MeSO_4][Me_2PO_4]$, **2**

χ $[Me_2PO_4]^-$	T_{onset} (°C)
0	371
0.336	278
0.587	282
1	282

$[C_4C_1im][OTf][NTf_2]$, **3**

χ $[NTf_2]^-$	T_{onset} (°C)
0	410
0.209	419
0.454	412
0.626	418
1	438

$[C_4C_1im][(HOC_2)_3C_1N][MeSO_4]$, **4**

χ $[(HOC_2)_3C_1N]^+$	T_{onset} (°C)
0	371
0.201	241
0.391	242
0.630	228
0.811	221
1	219

$[C_4C_1im][MeSO_4][NTf_2]$, **5**

χ $[NTf_2]^-$	T_{onset} (°C)
0	371
0.239	361
0.517	368
0.746	345
1	438

$[C_4C_1pyrr][NTf_2][Me_2PO_4]$, **6**

χ $[NTf_2]^-$	T_{onset} (°C)
0	275
0.285	250
0.445	256
0.600	249
1	428

$[C_4C_1im][NTf_2][Me_2PO_4]$, **7**

χ $[NTf_2]^-$	T_{onset} (°C)
0	282
0.150	279
0.271	266
0.387	282
0.500	280
0.690	292
0.838	274
1	438

$[C_4C_1im][Me\text{-}DBU][MeSO_4]$, **8**

χ $[Me\text{-}DBU]^+$	T_{onset} (°C)
0	371
0.359	336
0.637	332
1	297

$[C_4C_1im][C_1C_1C_1pz][OTf]$, **9**

χ $[C_1C_1C_1pz]^+$	T_{onset} (°C)
0	410
0.266	365
0.510	372
0.736	377
1	395

$[C_4C_1im][Me\text{-}DBU][MeSO_4][NTf_2]$, **10**

χ $[Me\text{-}DBU][MeSO_4]$	T_{onset} (°C)
0	438
0.207	355
0.418	358
0.602	355
0.814	333
1	297

$[C_4C_1im][C_4C_1pyrr][OTf][NTf_2]$, **11**

χ $[C_4C_1pyrr][NTf_2]$	T_{onset} (°C)
0	410
0.119	419
0.213	406
0.398	417
0.623	414
0.767	439
1	428

9. Computational procedures

DFT calculations were performed using the GAUSSIAN 09 suite of programs.[3] The B3LYP (Becke's three-parameter exchange[4] in combination with the Lee, Yang, Parr correlation[5]) functional was employed for all calculations together with the 6-311+G(d,p) basis set.

Convergence criteria were tightened above the default Gaussian values to 10^{-9} on the RMS density matrix and 10^{-7} on the energy. In addition, the numerical integration grid was enhanced from the default, with an optimized grid of 99 radial shells and 590 angular points per shell. The tighter convergence criteria were employed for all calculations (volume, charge arm). All optimized structures were confirmed as minima by frequency analysis. All calculations were performed under no symmetry constraints. Volume calculations were performed for all cations and anions, using the 'volume', 'iop(6/45=100)' and 'iop(6/66=20)' keywords. Volume calculations were performed 10 times for each ion conformation in order to obtain approximate error values, extreme outliers were omitted. Raman calculations were carried out using the 'freq=raman' keyword.

10. Molar volume calculations, charge arm calculations and Raman spectroscopy

Table E7 Calculated molar volumes, V_m, and charge arm lengths, R_{ca},[6] of the investigated ions. All calculations were performed at the B3LYP / 6-311+G(d,p) level of theory. Molar volumes were obtained from 10 repeats.

Ion	V_m (cm^3 mol^{-1})	R_{ca} (Å)
$[C_4C_1im]^+$	118 ± 7	1.20
$[C_4C_1pyrr]^+$	132 ± 6	0.63
$[C_1C_1C_1pz]^+$	94 ± 3	0.51
$[Me\text{-}DBU]^+$	142 ± 11	0.44
$[(HOC_2)_3C_1N]^+$ *a*	132 ± 7	1.56
$[(HOC_2)_3C_1N]^+$ *b*	131 ± 6	0.50

Ion	V_m (cm^3 mol^{-1})	R_{ca} (Å)
Cl^-	32 ± 5	0
$[MeSO_4]^-$	68 ± 6	1.04
$[OTf]^-$	70 ± 9	0.52
$[NTf_2]^-$ *cis*	130 ± 4	0.81
$[NTf_2]^-$ *trans*	129 ± 8	0.03
$[Me_2PO_4]^-$ *a*	87 ± 5	0.49
$[Me_2PO_4]^-$ *b*	88 ± 5	1.42
$[Me_2PO_4]^-$ *c*	90 ± 7	1.33

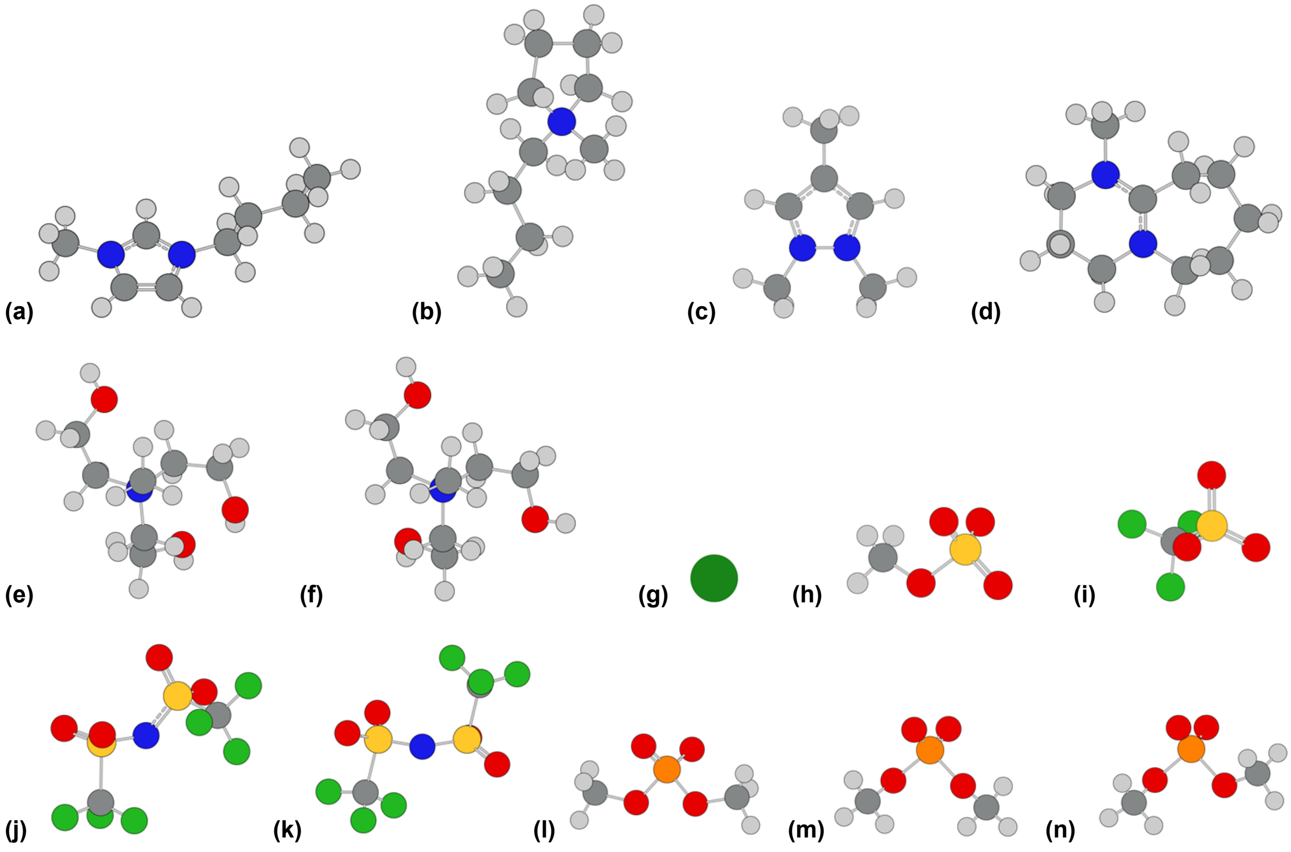

Fig. E9 Cations and anions, optimized at the B3LYP / 6-311+G(d,p) level of theory: (a) $[C_4C_1im]^+$; (b) $[C_4C_1pyrr]^+$; (c) $[C_1C_1C_1pz]^+$; (d) $[Me\text{-}DBU]^+$; (e) $[(HOC_2)_3C_1N]^+$ *a*; (f) $[(HOC_2)_3C_1N]^+$ *b*; (g) Cl^-; (h) $[MeSO_4]^-$; (i) $[OTf]^-$; (j) $[NTf_2]^-$ *cis*; (k) $[NTf_2]^-$ *trans*; (l) $[Me_2PO_4]^-$ *a*; (m) $[Me_2PO_4]^-$ *b*; (n) $[Me_2PO_4]^-$ *c*.

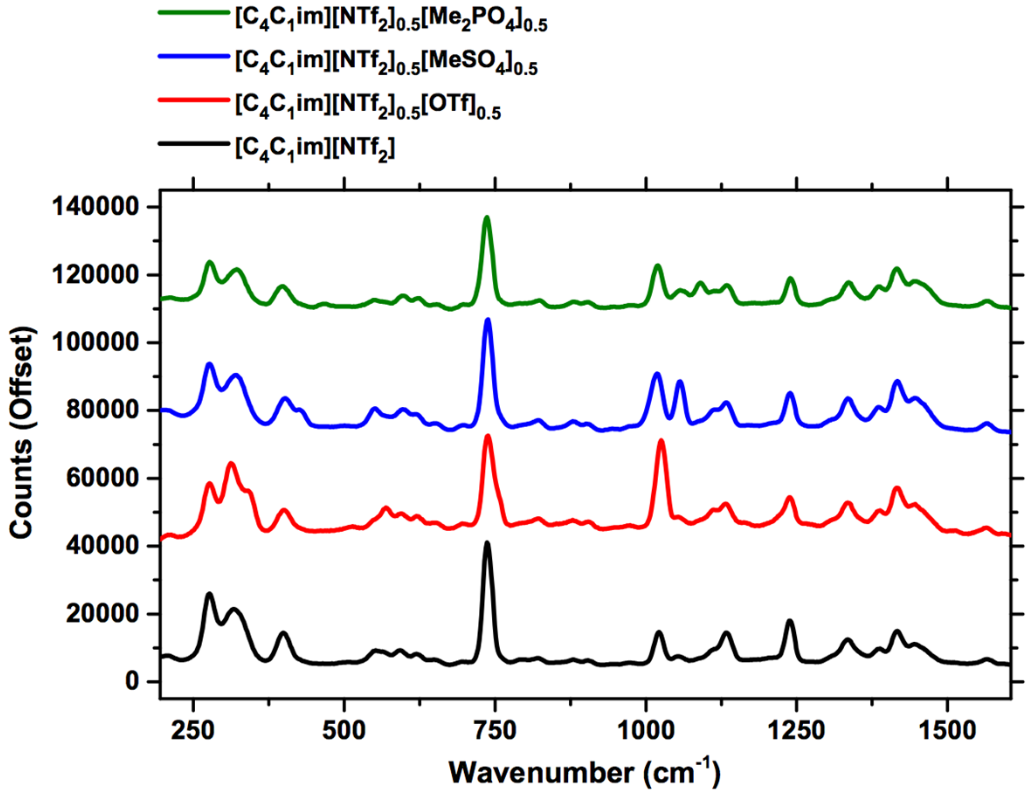

(a)

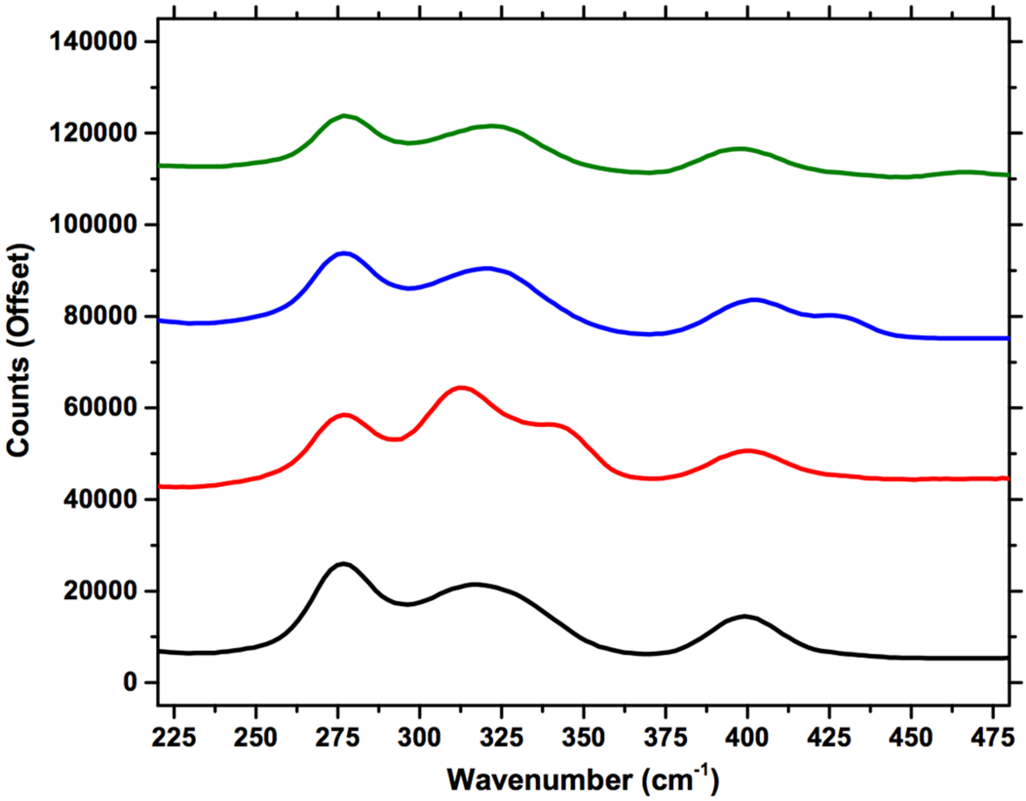

(b)

Fig. E10 Raman spectra for neat ionic liquid [C_4C_1im][NTf_2], and binary ionic liquid mixtures [C_4C_1im][NTf_2]$_{0.5}$[OTf]$_{0.5}$, **3**, [C_4C_1im][NTf_2]$_{0.5}$[$MeSO_4$]$_{0.5}$, **5**, and [C_4C_1im][NTf_2]$_{0.5}$[Me_2PO_4]$_{0.5}$, **7**: (a) Full spectra; (b) in the region 220 - 480 cm^{-1}. The exposure time for each spectrum was 2 seconds.

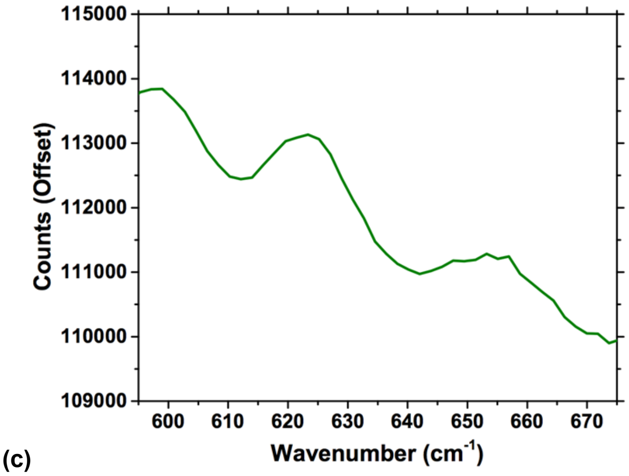

(c)

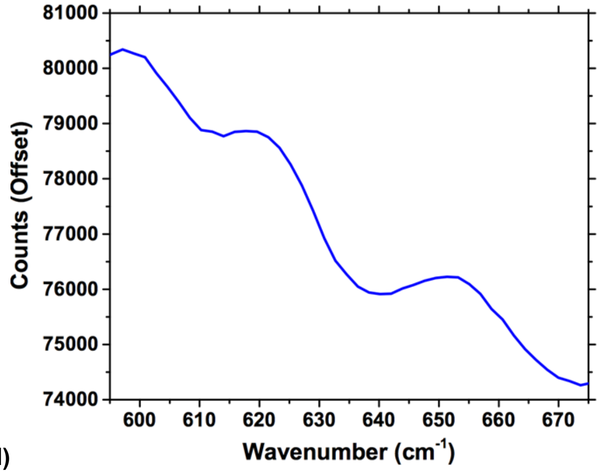

(d)

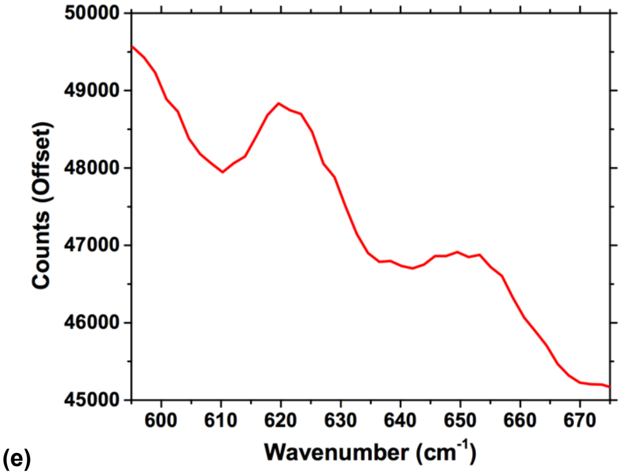

(e)

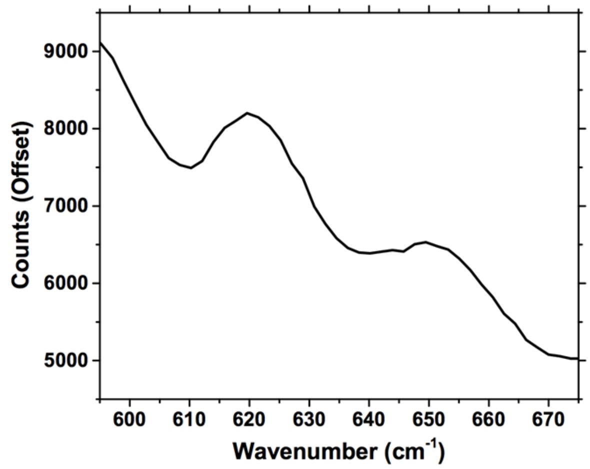

(f)

Fig. E10 continued Raman spectra in the region 595 - 675 cm^{-1}, for binary mixtures $[C_4C_1im][NTf_2]_{0.5}[Me_2PO_4]_{0.5}$, **7**, (c), $[C_4C_1im][NTf_2]_{0.5}[MeSO_4]_{0.5}$, **5**, (d), $[C_4C_1im][NTf_2]_{0.5}[OTf]_{0.5}$, **3,** (e), and neat ionic liquid $[C_4C_1im][NTf_2]$, (f). The exposure time for each spectrum was 2 seconds.

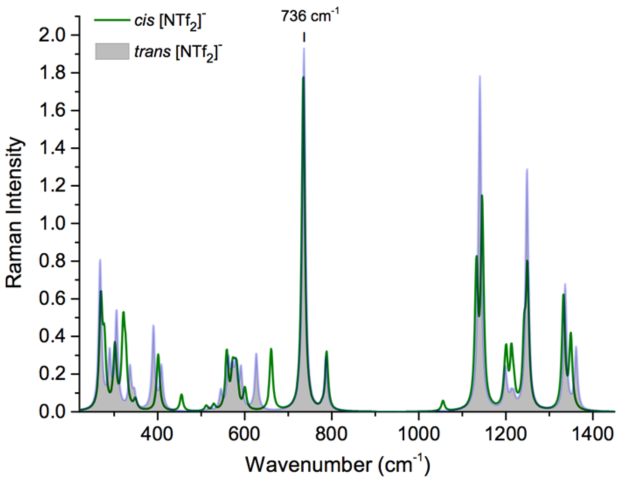

(g)

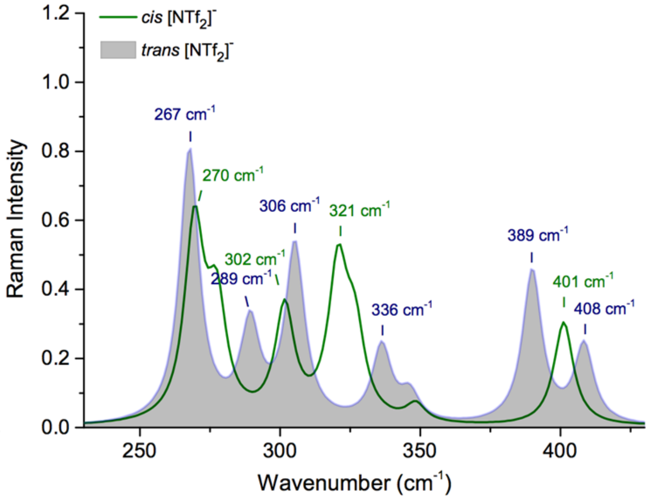

(h)

Fig. E10 continued DFT Raman spectra for the isolated *bis*(trifluoromethanesulfonyl)imide, $[NTf_2]^-$, anion, calculated at the B3LYP / 6-311+G(d,p) level of theory: (g) full spectra; (h) in the region 230 - 430 cm^{-1}.

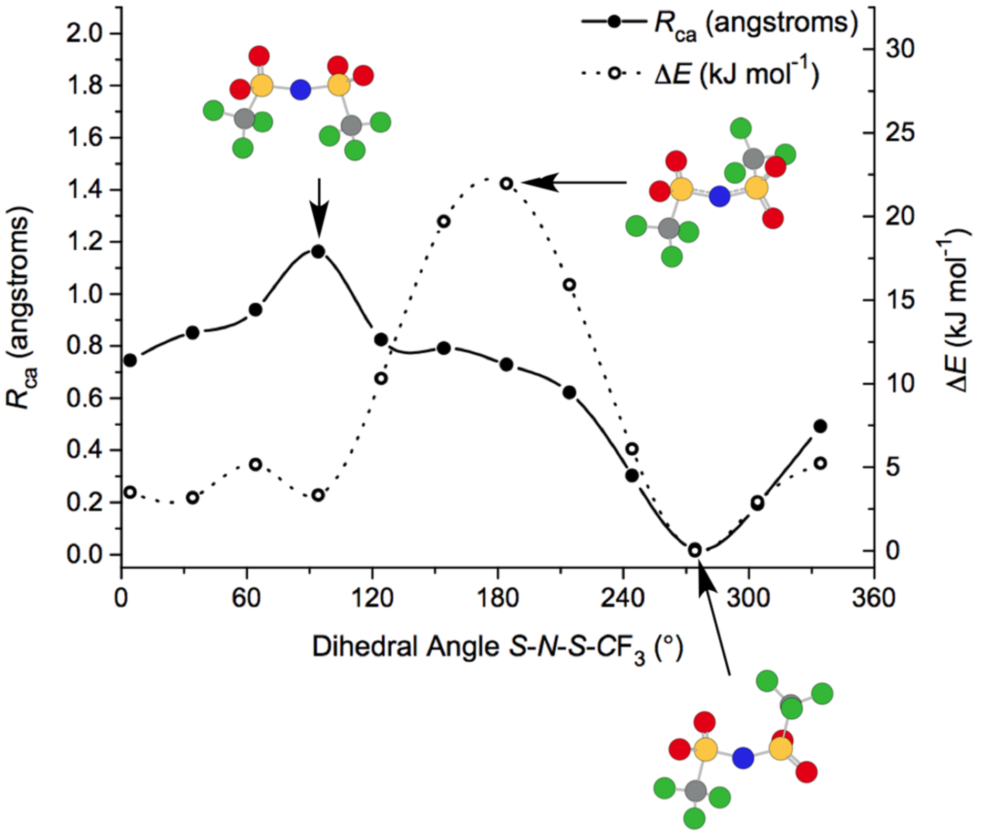

(a)

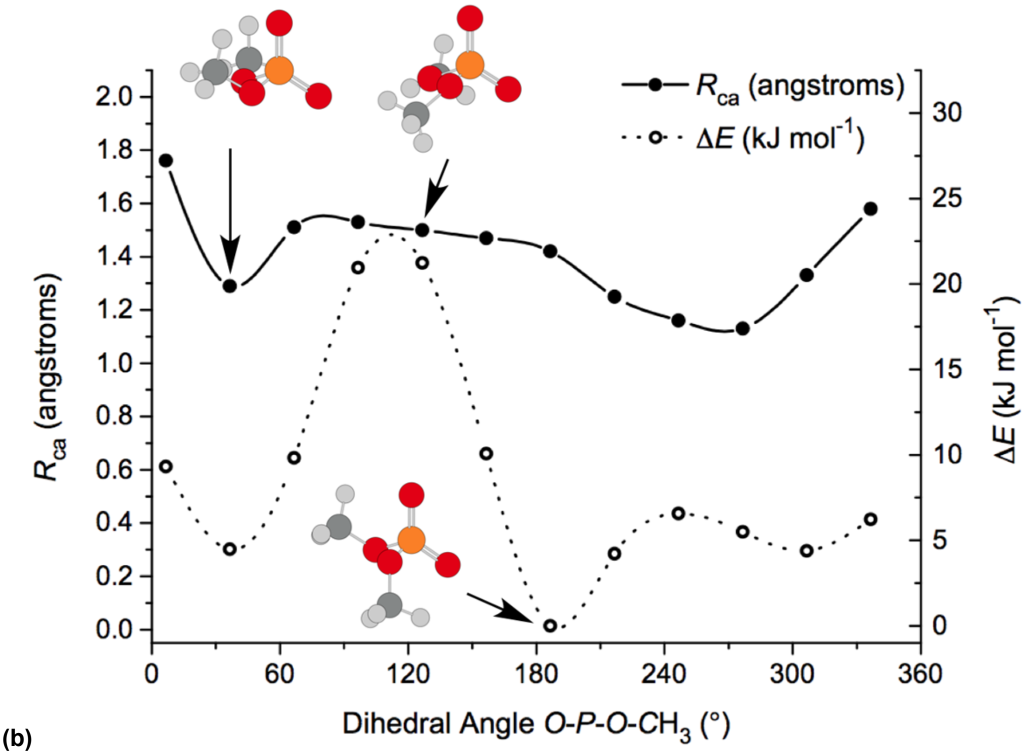

(b)

Fig. E11 Diagram demonstrating the influence of ion geometry on both the charge arm, R_{ca}, and the SCF energy, ΔE (kJ mol^{-1}) for anions: (a) *bis*(trifluoromethanesulfonyl)imide, $[NTf_2]^-$; (b) dimethyl phosphate, $[Me_2PO_4]^-$. ΔE energy values are uncorrected.

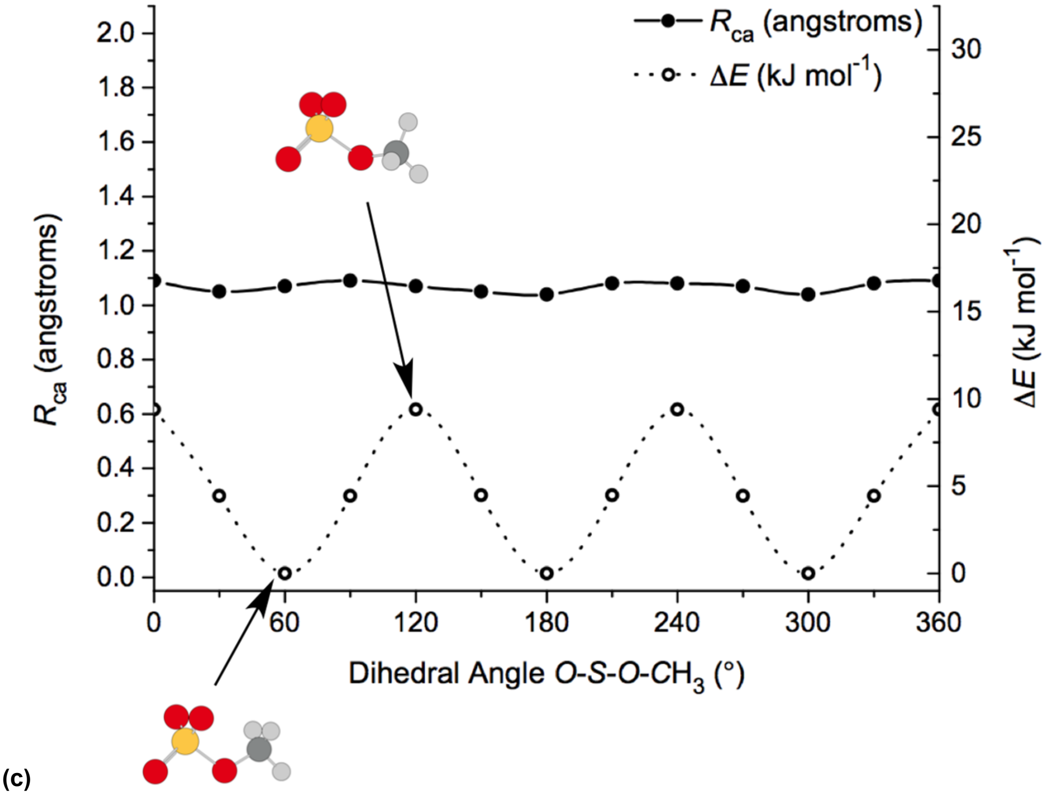

(c)

Fig. E11 continued Diagram demonstrating the influence of ion geometry on both the charge arm, R_{ca}, and the SCF energy, ΔE (kJ mol^{-1}) for anion: (c) methyl sulfate, $[MeSO_4]^-$. ΔE energy values are uncorrected.

Table E8 (a) Calculated charge arm lengths, R_{ca},[6] for $[C_nC_1im]^+$ (n = 1 - 10) cations, investigated previously by Canongia Lopes *et al* [7]; (b), (c), (d) Calculated charge arm lengths, R_{ca}, for $[C_n(C_1)_3N]^+$ (n = 1 - 14) cations and $[C_nCO_2]^-$, $[C_nSO_4]^-$ anions (n = 1 - 14). All calculations were performed at the B3LYP / 6-311+G(d,p) level of theory, using the NBO charges on atoms.

(a)

$[C_nC_1im]^+$ n =	R_{ca} (Å)
1	0.16
2	0.36
3	0.75
4	1.20
5	1.70
6	2.22
7	2.77
8	3.33
9	3.90
10	4.49

(b)

$[C_n(C_1)_3N]^+$ n =	R_{ca} (Å)
1	0.00
2	0.30
3	0.70
4	1.17
5	1.68
6	2.22
7	2.77
8	3.34
9	3.93
10	4.51
11	5.12
12	5.72
13	6.33
14	6.94

(c)

$[C_nCO_2]^-$ n =	R_{ca} (Å)
1	0.97
2	1.31
3	1.83
4	2.31
5	2.92
6	3.49
7	4.04
8	4.67
9	5.23
10	5.87
11	6.45
12	7.10
13	7.74
14	8.35

(d)

$[C_nSO_4]^-$ n =	R_{ca} (Å)
1	1.07
2	1.46
3	1.87
4	2.33
5	2.81
6	3.32
7	3.85
8	4.40
9	4.96
10	5.53
11	6.10
12	6.69
13	7.28
14	7.87

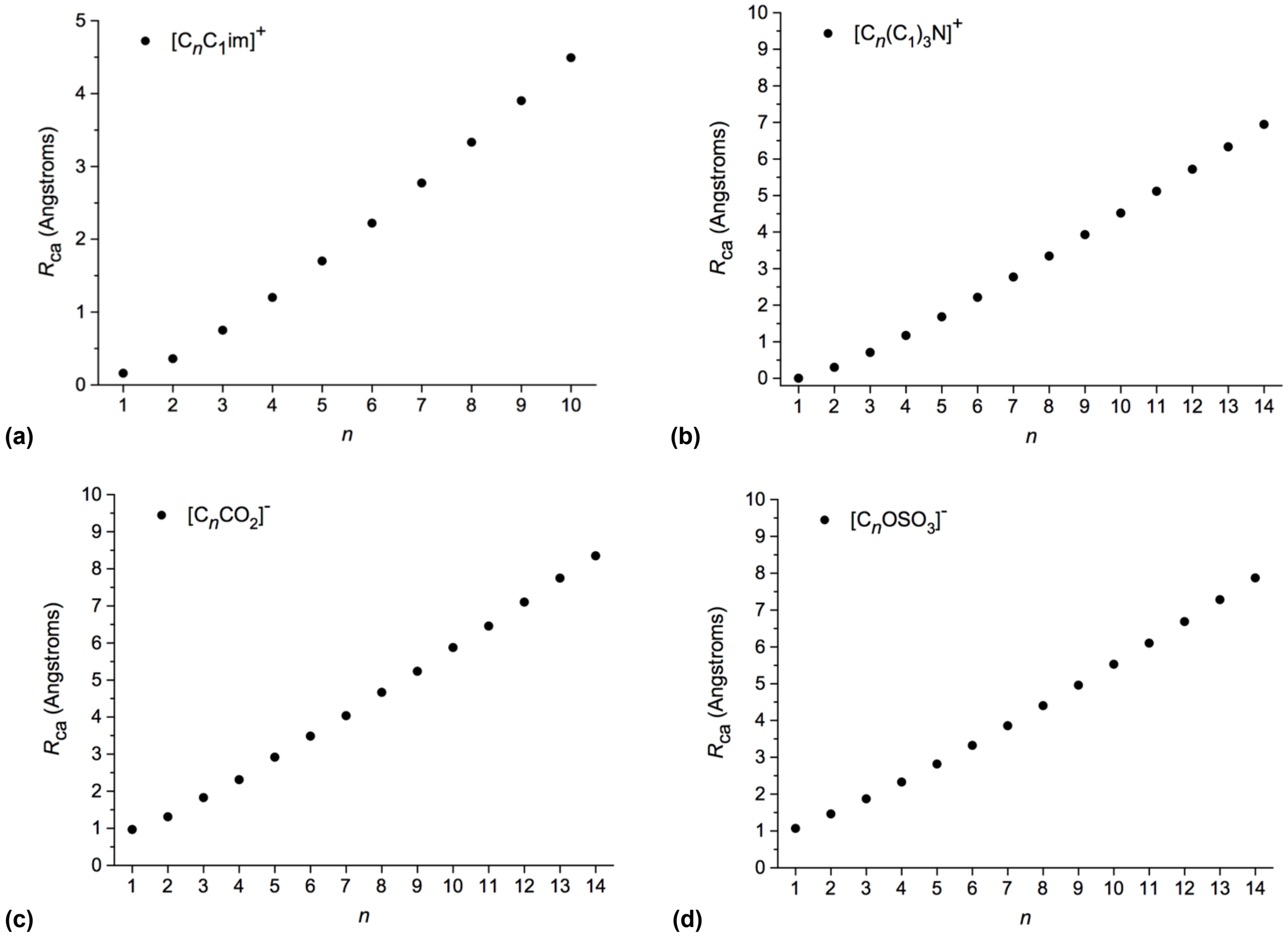

Fig. E12 (a) Graph representing the charge arm length, R_{ca}, for $[C_nC_1im]^+$ (n = 1 - 10) cations, investigated previously by Canongia Lopes *et al* [7]; (b), (c), (d) Graphs representing the charge arm lengths, R_{ca}, for $[C_n(C_1)_3N]^+$ (n = 1 - 14) cations and $[C_nCO_2]^-$, $[C_nSO_4]^-$ anions (n = 1 - 14). All calculations were performed at the B3LYP / 6-311+G(d,p) level of theory, using the NBO charges on atoms.

References

1. M. P. Cecchini, M. A. Stapountzi, D. W. McComb, T. Albrecht and J. B. Edel, *Anal. Chem.*, 2011, **83**, 1418.
2. M. P. Cecchini, J. Hong, C. Lim, J. Choo, T. Albrecht, A. J. Demello and J. B. Edel, *Anal. Chem.*, 2011, **83**, 3076.
3. Gaussian 09, Revision B.01, M. J. Frisch, G. W. Trucks, H. B. Schlegel, G. E. Scuseria, M. A. Robb, J. R. Cheeseman, G. Scalmani, V. Barone, B. Mennucci, G. A. Petersson, H. Nakatsuji, M. Caricato, X. Li, H. P. Hratchian, A. F. Izmaylov, J. Bloino, G. Zheng, J. L. Sonnenberg, M. Hada, M. Ehara, K. Toyota, R. Fukuda, J. Hasegawa, M. Ishida, T. Nakajima, Y. Honda, O. Kitao, H. Nakai, T. Vreven, J. A. Montgomery, Jr., J. E. Peralta, F. Ogliaro, M. Bearpark, J. J. Heyd, E. Brothers, K. N. Kudin, V. N. Staroverov, R. Kobayashi, J. Normand, K. Raghavachari, A. Rendell, J. C. Burant, S. S. Iyengar, J. Tomasi, M. Cossi, N. Rega, J. M. Millam, M. Klene, J. E. Knox, J. B. Cross, V. Bakken, C. Adamo, J. Jaramillo, R. Gomperts, R. E. Stratmann, O. Yazyev, A. J. Austin, R. Cammi, C. Pomelli, J. W. Ochterski, R. L. Martin, K. Morokuma, V. G. Zakrzewski, G. A. Voth, P. Salvador, J. J. Dannenberg, S. Dapprich, A. D. Daniels, Ö. Farkas, J. B. Foresman, J. V. Ortiz, J. Cioslowski, and D. J. Fox, Gaussian, Inc., Wallingford CT, 2010.
4. A. D. Becke, *J. Chem. Phys.*, 1993, **98**, 5648.
5. C. Lee, W. Yang and R. G. Parr, *Phys. Rev. B: Condens. Matter*, 1988, **37**, 785.
6. H. Niedermeyer, M. A. Ab Rani, P. D. Lickiss, J. P. Hallett, T. Welton, A. J. P. White and P. A. Hunt, *Physical Chemistry Chemical Physics*, 2010, **12**, 2018.
7. J. N. Canongia Lopes, T. C. Cordeiro, J. M. S. S. Esperança, H. J. R. Guedes, S. Huq, L. P. N. Rebelo and K. R. Seddon, *J. Phys. Chem. B*, 2005, **109**, 3519.
